# Supplementary material for: Ethnic differences in early onset multimorbidity and associations with health service use, long-term prescribing, years of life lost, and mortality: A cross-sectional study using clustering in the UK Clinical Practice Research Datalink
Source: PLoS Med. 2023 Oct 27;20(10):e1004300. doi: 10.1371/journal.pmed.1004300 (PMC10610074; doi:10.1371/journal.pmed.1004300)
Supplement: S1 Protocol — (PDF) [file pmed.1004300.s001.pdf]

Medicines & Healthcare products Regulatory Agency

CPRD

THIS DOCUMENT IS FOR REFERENCE ONLY. ALL APPLICATIONS SHOULD BE SUBMITTED VIA eRAP AT www.erap.cprd.com

PART 1: APPLICATION FORM

GENERAL INFORMATION ABOUT THE PROPOSED RESEARCH STUDY

1. Study Title (Max. 255 characters including spaces)

Characterisation of multimorbidity clusters and trajectories using data-driven approaches in a nationally-representative population

2. Research Area (place '**X**' in all boxes that apply)

Drug Safety		Economics	
Drug Utilisation	X	Pharmacoeconomics	
Drug Effectiveness		Pharmacoepidemiology	
Disease Epidemiology	X	Methodological	x
Health Services Delivery	X		

3. Does this protocol describe an observational study using purely CPRD data?

Yes	X	No	

4. Does this protocol involve requesting any additional information from GPs, or contact with patients?

Yes		No	X

If yes, provide the reference number:

5. Chief Investigator

Title:	Doctor
Full name:	Sarah Finer
Job title:	Clinical Senior Lecturer in Diabetes; Honorary Consultant in Diabetes
Affiliation/organisation:	Institute of Population Health Sciences, Queen Mary University of London
Email address:	s.finer@qmul.ac.uk
CV Number (if applicable):	
Will this person be analysing the data? (Y/N)	Yes

6. Corresponding Applicant

Title:	Doctor
Full name:	Fabiola Eto
Job title:	Research Fellow
Affiliation/organisation:	Institute of Population Health Sciences, Queen Mary University of London
Email address:	f.eto@qmul.ac.uk
CV Number (if applicable):	
Will this person be analysing the data? (Y/N)	Yes

Medicines & Healthcare products Regulatory Agency

CPRD

7. List of all investigators/collaborators

Title:	Professor
Full name:	Michael Barnes
Job title:	Professor Of Bioinformatics
Affiliation/organisation:	William Harvey Research Institute, Queen Mary University of London
Email address:	m.r.barnes@qmul.ac.uk
CV Number (if applicable):	
Will this person be analysing the data? (Y/N)	Yes

Title:	Dr
Full name:	Rohini Mathur
Job title:	Assistant Professor
Affiliation/organisation:	Faculty of Epidemiology and Population Health, London School of Hygiene and Tropical Medicine
Email address:	rohini.mathur@lshtm.ac.uk
CV Number (if applicable):	316_15CESL
Will this person be analysing the data? (Y/N)	No

Title:	Dr
Full name:	Sally Hull
Job title:	Clinical Reader
Affiliation/organisation:	Institute of Population Health Sciences, Queen Mary University of London
Email address:	s.a.hull@qmul.ac.uk
CV Number (if applicable):	
Will this person be analysing the data? (Y/N)	No

Title:	Professor
Full name:	Steph Taylor
Job title:	Professor Of Public Health & Primary Care
Affiliation/organisation:	Institute of Population Health Sciences, Queen Mary University of London
Email address:	s.j.c.taylor@qmul.ac.uk
CV Number (if applicable):	
Will this person be analysing the data? (Y/N)	No

Title:	Professor
Full name:	Nick Reynolds
Job title:	Professor
Affiliation/organisation:	Institute of Cellular Medicine/Newcastle University
Email address:	nick.reynolds@ncl.ac.uk
CV Number (if applicable):	
Will this person be analysing the data? (Y/N)	No

Title:	Professor
Full name:	Deborah Swinglehurst
Job title:	Professor Of Primary Care
Affiliation/organisation:	Institute of Population Health Sciences, Queen Mary University of London
Email address:	d.swinglehurst@qmul.ac.uk
CV Number (if applicable):	
Will this person be analysing the data? (Y/N)	No

Title:	Dr
Full name:	Miriam Samuel
Job title:	Academic Clinical Fellow in Primary Care
Affiliation/organisation:	Institute of Population Health Sciences, Queen Mary University of London
Email address:	miriam.samuel@nhs.net
CV Number (if applicable):	
Will this person be analysing the data? (Y/N)	Yes

Title:	Mrs
Full name:	Tahania Ahmad
Job title:	PhD Researcher
Affiliation/organisation:	Institute of Population Health Sciences, Queen Mary University of London
Email address:	t.a.ahmad@qmul.ac.uk
CV Number (if applicable):	
Will this person be analysing the data? (Y/N)	Yes

Title:	Dr
Full name:	Rafael Henkin
Job title:	Data Engineer
Affiliation/organisation:	William Harvey Research Institute, Queen Mary University of London
Email address:	r.henkin@qmul.ac.uk
CV Number (if applicable):	
Will this person be analysing the data? (Y/N)	Yes

Title:	Miss
Full name:	Alisha Angdembe
Job title:	Data Scientist
Affiliation/organisation:	William Harvey Research Institute, Queen Mary University of London
Email address:	a.angdembe@qmul.ac.uk
CV Number (if applicable):	
Will this person be analysing the data? (Y/N)	Yes

ACCESS TO THE DATA

8. Sponsor of the study

Institution/Organisation:	Queen Mary University of London
Address:	Mile End Rd, Bethnal Green, London E1 4NS

9. Funding source for the study

Same as Sponsor?	Yes		No	X
Institution/Organisation:	Medical Research Council			
Address:	2nd Floor David Phillips Building, Polaris House, North Star Avenue, Swindon, SN2 1ET			

Same as Sponsor?	Yes		No	X
Institution/Organisation:	National Institute for Health Research			
Address:	NIHR CCF, Grange House, 15 Church Street, Twickenham, TW1 3NL			

10. Institution conducting the research

Institute of Population Health Sciences, Queen Mary University of London
Yvonne Carter Building. 58 Turner Street. London E1 2AB

Medicines & Healthcare products Regulatory Agency

CPRD

Same as Sponsor?	Yes		No	
Institution/Organisation:				
Address:				

11. Data Access Arrangements

Indicate with an '**X**' the method that will be used to access the data for this study:

Study-specific Dataset Agreement	X

Institutional Multi-study Licence

Institution Name	
Institution Address	

Will the dataset be extracted by CPRD?

Yes	X	No		

If yes, provide the reference number: 00070707

12. Data Processor(s):

Processing X	
Accessing X	
Storing X	
Processing area (UK/EEA/Worldwide)	UK
Organisation name	Queen Mary University of London
Organisation address	Mile End Rd, Bethnal Green, London E1 4NS

Processing		
Accessing		
Storing		
Processing area (UK/EEA/Worldwide)		
Organisation name	Queen Mary University of London	
Organisation address	Mile End Rd, Bethnal Green, London E1 4NS	

[Add more processors as necessary by copy and pasting a new table for each processor]

INFORMATION ON DATA

13. Primary care data (place '**X**' in all boxes that apply)

CPRD GOLD	X	CPRD Aurum	X

Reference number (if applicable): 00068753

14. Please select any linked data or data products being requested

Patient Level Data (place '**X**' in all boxes that apply)

ONS Death Registration Data	X		
HES Admitted Patient Care	X		
HES Outpatient			
HES Accident and Emergency		NCRAS Cancer Registration Data	
HES Diagnostic Imaging Dataset		NCRAS Cancer Patient Experience Survey (CPES) data	
HES PROMS (Patient Reported Outcomes Measure)		NCRAS Systemic Anti-Cancer Treatment (SACT) data	
CPRD Mother Baby Link		NCRAS National Radiotherapy Dataset (RTDS) data	
Pregnancy Register	X	NCRAS Quality of Life Cancer Survivors Pilot (QOLP)	
Mental Health Data Set (MHDS)		NCRAS Quality of Life Colorectal Cancer Survivors (QOLC)	

Area Level Data (place '**X**' in one Practice / Patient level box that may apply)

**Practice level (UK)**		**Patient level (England only)**	
Practice Level Index of Multiple Deprivation	X	Patient Level Index of Multiple Deprivation	X
Practice Level Index of Multiple Deprivation (index other than the most recent)		Patient Level Index of Multiple Deprivation Domains	
Practice Level Index of Multiple Deprivation Domains		Patient Level Carstairs Index for 2011 Census	

Practice Level Carstairs Index for 2011 Census (Excluding Northern Ireland)		Patient Level Townsend Score	
2011 Rural-Urban Classification at LSOA level		2011 Rural-Urban Classification at LSOA level	

Reference / Protocol number (where applicable):

15. Are you requesting linkage to a dataset not listed above?

Yes		No	X

If yes, provide the Non-Standard Linkage reference number:

16. Does any person named in this application already have access to any of these data in a patient identifiable form, or associated with an identifiable patient index?

Yes		No	X

If yes, provide further details:

Medicines & Healthcare products Regulatory Agency

CPRD

PART 2: PROTOCOL INFORMATION

Applicants must complete all sections
**A. Lay Summary (Max. 250 words)**<br><br>Multimorbidity describes the situation where an individual is affected by 2 or more health conditions. Studies have shown that multimorbidity is becoming more common, and the National Health Service does not currently have services designed to tackle it well. People living with multimorbidity often need to take multiple medications and require regular contact with health services. Recent research has shown that multimorbidity exists in 'clusters', with groups of common conditions (e.g. type 2 diabetes, chronic pain, and depression) often co-existing.<br><br>Using the health records of several million people held by CRPD, we will explore unanswered questions relating to multimorbidity. Our research will focus on using novel statistical and computer science techniques to look for clusters and patterns in this health record data. We will examine these clusters and patterns of multimorbidity to better understand how they affect particular people (e.g. people prescribed specific or multiple medications, or people living with specific conditions such as cancers and human immunodeficiency virus (HIV)) and populations (e.g. people from different ethnic groups). Our approach, in combination with using historic data available from CPRD, will also allow us to uncover how and why multimorbidity develops over time, and if it is associated with early death.<br><br>We expect that the impact of our research will be wide-ranging, including supporting improvements in health and social care for multimorbidity and perhaps more efficient use of limited NHS funds.
**B. Technical Summary (Max. 300 words)**<br><br>Our proposal seeks to deliver new knowledge on multimorbidity through the application of hypothesis-generating, data-driven analytical tools to a large-scale electronic health record dataset from a nationally-representative population. We seek to expand on previous multimorbidity research (which focuses predominantly on 30-40 common chronic conditions) to define clusters and trajectories of multimorbidity across ~200 long-term conditions. This large set of conditions have been defined through a rigorous consensus-building process and extensive review of existing validated code sets (including previously-published CPRD studies undertaken by CALIBER and the Cambridge Multimorbidity group (1–3)).<br><br>We will initiate our analyses using a cross-sectional design, applying data-driven clustering (unsupervised machine learning) to identify clusters of multimorbidity in England population across our extensively-curated ~ 200 long-term conditions obtained from primary care data and linked Hospital Episode Statistics (HES). We will stratify our analysis by ethnicity to identify how multimorbidity clusters across ethnic groups (including White, Black African and Caribbean, and south Asian).<br><br>Subsequent to this hypothesis-generating clustering, we will use an observational cohort design to investigate how multimorbidity clusters develop from index conditions and how they change over time, including to death (using linked Office for National Statistics mortality data). At this point, we will focus on a limited number of multimorbidity clusters (up to 8) selected to ensure we generate new knowledge, e.g. clusters which include less-studied conditions such as cancer or human immunodeficiency virus, as well as those where there is variation by ethnicity. We will then use epidemiological analyses, including Cox model, adjusted by socioeconomic characteristics (age, sex, ethnicity, Index of Multiple Deprivation), and time-dependent variables (e.g. risk factors, clinical measurements) to characterise these trajectories. Additionally, we will apply artificial intelligence techniques (including supervised and unsupervised machine learning) for focused analyses to examine the interrelationship between specific multimorbidity clusters under study and prescribing/polypharmacy patterns.
**C. Outcomes to be Measured**<br><br>**Primary outcome(s):**

(a) The clusters of multimorbidity*; and,
(b) The multimorbidity cluster trajectories throughout the follow up period.

*Multimorbidity will be defined as the presence of two or more long-term conditions (out of the clinical consensus-derived 220 conditions described in appendix 1) within the same individual. Unsupervised machine learning algorithms applied to the dataset will then derive and define clusters of multimorbidity, by grouping similar entities together (using an understanding of difference in distance between data points). A sample of code sets used to select conditions are shown in Appendix 2.

Secondary outcomes:
(a) Index condition to multimorbidity cluster; and,
(b) Multimorbidity cluster to death

D. Objectives, Specific Aims and Rationale

Our strategic objective is to understand multimorbidity clusters and their trajectories through the application of advanced data analysis. We seek to expand on existing multimorbidity studies by incorporating a broad set of health conditions, selected by a rigorous clinical consensus exercise (see Appendix 1). We will meet our objectives through the use of a large, high quality electronic health record (EHR) dataset representative of the UK population.

Our specific research aims are as follows:

A) To characterise multimorbidity clusters using data-driven methodology that expands current definitions and broadens relevance to ethnic minorities;
B) To characterise the trajectory of multimorbidity clusters across the life course to death, and to describe their determinants;
C) To describe the relationship between the patterns of multimorbidity identified in A) and B) with prescribing/polypharmacy.

Rationale: The need to better understand and describe patterns of multimorbidity has been acknowledged by the recent Academy of Medical Sciences report (4). This report highlights the need to build on existing studies of multimorbidity (which mostly use a limited set of ~40 single conditions, based on the original work by Barnett et al (5)), and expand the definition of multimorbidity to incorporate a wider set of chronic conditions. There is also a lack of knowledge of how multimorbidity varies according to ethnicity, and early studies suggest significant differences, e.g in east London, British south Asians are twice as likely to have cardiovascular multimorbidity than White people (6). Black Africans and Black Caribbeans are reported to have similarly high rates of multimorbidity including diabetes and depression in south London (7). Most existing studies of multimorbidity use cross-sectional data to estimate prevalence and there is limited knowledge on the longitudinal trajectories from single ‘index’ conditions to multimorbidity, what their determinants are, and what their wider impact is on prescribing and healthcare utilisation.

Combining powerful, data-driven analysis methods with high-quality, linked electronic health record data from a nationally-representative dataset, offers an opportunity to build on these knowledge gaps and develop a new understanding of multimorbidity.

E. Study Background

Multimorbidity is increasingly recognised as an important research priority, due to its increasing prevalence and its significant burden on health, health systems, and society in the UK and globally. However, the recent Academy of Medical Sciences report (4) identifies the significant gaps in our knowledge of multimorbidity, and highlights the need to define broader clusters of multimorbid conditions, study their development during the life course, and understand their association with ethnicity and social deprivation.

Most existing multimorbidity research includes a maximum of ~40 single conditions using diagnoses whose recording in EHR is incentivised through the Quality and Outcomes Framework for NHS primary care (3,5,8).

However, these definitions fail to include numerous conditions known to closely cluster with other conditions and with high impact to the individual and health services. Examples of such conditions that are understudied in existing multimorbidity research include human immunodeficiency virus (HIV) and cancer, and these may underlie some ethnic variation in multimorbidity (9). High quality, large-scale electronic health record data, e.g. from CPRD, offers an exciting opportunity to build on existing definitions of multimorbidity and expand their scope using advanced data analysis. We propose doing so by incorporating a larger set of chronic conditions (n~200) for inclusion, defined through a rigorous consensus-building exercise (see Appendix 1), and through the application of data clustering techniques that can support high-throughput and data-driven analyses. We will undertake entirely data-driven, unbiased clustering of all ~200 conditions. We anticipate identifying multimorbidity clusters based around common chronic diseases such as diabetes and hypertension but will additionally examine the performance of our multimorbidity clustering around a limited number of specific low- and medium-prevalence conditions (HIV and specific cancers, respectively), selected due to their high health impact, disproportionate impact on minority ethnic groups and increasing relevance to multimorbidity research (primarily due to increasing survival rates).

Currently, most studies of multimorbidity use cross-sectional data sources and rarely characterise its emergence or trajectory across the life course. In the limited number of studies where these longitudinal analyses have been performed, important insights into the risk factors and potential causation of multimorbidity have been derived. For example, in a modest sample from Whitehall II cohort study data (n=8.270) with low rates of multimorbidity (6.6%) at age 50 years, an association between socioeconomic status and progression to multimorbidity was identified, but with unclear influence from clinical risk factors (10). Knowledge is needed from larger population-based longitudinal studies to better understand the trajectories to the development of multimorbidity clusters from risk factors and single index conditions. Electronic health records, particularly where linked data are available, also offer the ability to study trajectories across important life course events such as pregnancy, and to death. It is also important to consider the wider impact of multimorbidity on individuals experiencing it and health systems. Recent work using cross-sectional CPRD data identifies a close relationship of multimorbidity with number of prescriptions and healthcare use (11). Investigating these wider interrelationships further, e.g. by more detailed analysis of prescription data and using longitudinal data, offers the potential to elucidate causal relationships (e.g. between multimorbidity and the use of specific drugs) (12) and to identify opportunities for early detection and intervention.

Little is known about variation in multimorbidity between ethnic groups across the UK, but early studies suggest an important impact on the conditions included in multimorbidity clusters, as well as the trajectories to these clusters during the life course. In east London, British south Asians are twice as likely to have cardiovascular multimorbidity than White people (6). Black Africans and Black Caribbeans are reported to have similarly high rates of multimorbidity including diabetes and depression in south London (7). In both studies, ethnicity and socioeconomic deprivation were strongly co-correlated, highlighting the importance of looking across wider populations that are representative within the UK and relevant to wider global populations. Furthermore, ethnic minority groups are disproportionately impacted by conditions such as HIV and some cancers that may independently influence risk of multimorbidity. Our work will embed analyses to ensure that ethnic variation in multimorbidity is adequately assessed and understood.

Our analyses will utilise a combination of standard epidemiological analyses and machine learning techniques to describe prevalence patterns of multimorbidity conditions and their longitudinal progression. Our research will also build on increasingly used data-driven clustering techniques that support unbiased characterisation of structures/relationships between objects (in this case clinical conditions). By applying these techniques to a more extensive, and consensus-built list of clinical conditions that could contribute to multimorbidity, we expect to elucidate novel clusters unbiased by a priori knowledge or clinical selection. The multimorbidity clusters that we identify will be characterised further through analysis of their trajectories across the lifecourse, their determinants, and their associations with prescribing/polypharmacy and healthcare utilisation.

This discrete project will link to two interrelated and synergistic research projects already underway, funded through the recent strategic UKRI multimorbidity funding streams. First, an MRC grant awarded to the lead applicant (with R. Mathur as Co-Investigator) studying “Multimorbidity clusters, trajectories and genetic risk in British south Asians”, and second, an NIHR AIM Development award (led by N. Reynolds, with S. Finer and M. Barnes as Co-Investigators), which aims to characterise the dynamic inter-relationships between multimorbidity and polypharmacy using artificial intelligence.

More distally, our work will build and methodological expertise relevant to a growing body of research on multimorbidity and polypharmacy, and other health data science projects that will build capacity and support future research in this area.This project is therefore likely to support, indirectly, related funded programmes elucidating

genetic causes of multimorbidity: the Wellcome Trust-funded Genes & Health study, and collaborative work with the LIfespaN multimorbidity research Collaborative, currently funded by an MRC Development award.

F. Study Type

This is a hypothesis-generating descriptive study that uses both cross-sectional and cohort designs.

G. Study Design

Research Aim A) We will use a cross-sectional design. For this analysis, we will include data from all active 'acceptable patients' (≥16 years of age) who were permanently registered to English General Practices between 1 January 2010 and 31st December 2020. The practices included will be those contributing with 'Up to standard' data during the study timeframe.

Research Aims B and C) We will use an observational cohort design. Considering that this is a hypothesis-generating study, our main aim is not to compare exposed and unexposed groups of patients, instead, we will evaluate the development of multimorbidity clusters to generate hypotheses on their trajectory and to help identify homogeneous groups of patients who may share similar prognosis and health care needs. We will assess these trajectories throughout the follow-up period and look at the potential factors that affect them, including where specific life course events occur, and in the context of specific prescribing patterns (particularly polypharmacy). The outcomes of this analysis will be the development of multimorbidity clusters of diseases through the years, polypharmacy and mortality. We will censor the patients who have transferred out of their practice during the follow-up period.

H. Feasibility counts

We will include all people registered with a GP included in CPRD who meet inclusion criteria (see Definition of the Study Population) irrespective of whether they have multimorbidity or not. This will allow us to define multimorbidity using new criteria, and to determine its onset longitudinally. We have determined feasibility of our approach using a recently published study on multimorbidity by Cassel et al., 27.2% of the eligible CPRD population (n = 403,985) were identified as having two or more conditions on 1 January 2012. Our proposal will adopt similar criteria to select our study's population, i.e. in defining 'acceptable patients' and 'up to standard' data. In a cohort size of patients eligible for linkage using CPRD GOLD (n = 9,209,834) and considering a multimorbidity prevalence of 27.2% we anticipate identifying at least 2,505,075 patients with two or more conditions. Given the importance of understanding ethnic variation in multimorbidity, we have used Office for National Statistics ethnicity data to predict that of the 9,209,834 people in CPRD GOLD (assuming a representative population), approximately 700,000 will be people of Asian/Asian British ethnicity, and 300,000 will be people of Black/African/Caribbean/Black British ethnicities.

For CPRD Aurum data, in the estimated cohort size of 35,443,993 patients eligible for linkage, we expect to identify around 9,640,766patients with two or more conditions considering the same prevalence of multimorbidity identified by Cassel et al. (8) (27.2%). Assuming the representativity of CPRD Aurum data, and applying the prevalence of 7.8% and 3.5% of Asian/Asian British and Black/African/Caribbean/Black British ethnicities, we would have approximately 2,700,00 and 1,200,000 people of those respective ethnicities. However, we will select only the population of East London from Aurum dataset (please see section K. Definition of the Study Population) as these will form a validation set from an ethnically-diverse population.

It is important to highlight that the Cassel et al study population size provides a rough estimate of the expected number of patients available in CRPD with linked data set. However, by comparison to Cassel et al, we will include a longer timeframe (2010 to 2020), younger population (from 16 years old, rather than 18 years old) and a broader set of long-term conditions (220 rather than 36), thereby increasing our population size.

I. Sample size considerations

To estimate the minimum sample size necessary to meet our research aims, we have based our parameters in the prevalence of multimorbidity (≥ 2 conditions) of 27.2% found in the study of Cassel and colleagues (8). As this is an observational study, the sample size calculation was obtained using the following formula (13):

$$n = \frac{Z^2 P(1-P)}{d^2}$$

In this formula, n is the sample size and Z is the test statistic corresponding to 95% confidence level (Z = 1.96). The significance level was set at 5% (p=0.05). P is the expected prevalence of multimorbidity which we set to 27.2%. d is the precision of the estimate, corresponding to the margin of error in the prevalence of multimorbidity given by its confidence intervals from the Cassel study. We set the d to be 0.20%.

$$n = \frac{1.96^2\ 0.272\ (1-0.272)}{0.002^2}$$

$$n = 190{,}174$$

Z = 1.96
P = 27.2% = 0.272
1-P = 0.728
d = 0.2% = 0.002

Using the numbers given above, to identify the prevalence of multimorbidity among our population, minimum 190,174 subjects are required. Considering there is a possibility of 10% missing data, 209,191 subjects would be required.

Nevertheless, it is important to consider that Cassel' study included a list of only 36 conditions, and presented a higher prevalence of multimorbidity compared to previous studies on multimorbidity (5,14). If we were planning to replicate known common comorbidities, then the given sample size would be sufficient. However, we aim to generate new clusters using a data driven approach (which has a better performance with large amount of data) that incorporates lower prevalence diseases and more than 200 conditions. Therefore, assuming the inclusion of lower prevalence conditions, such as HIV (prevalence = 0.09%), we expect to need a larger sample size to build robust clusters that include these and that also allow us to investigate variation by ethnicity.

We are strongly guided by recent work undertaken by the CALIBER team (University College) who have undertaken a rigorous study of age-based trajectories of disease clusters (using nearly 300 conditions) using CPRD data (2,15). In this study, it was feasible to build data-driven disease clusters across 3 million people included in CPRD.

According to the release note of CPRD GOLD and Aurum database published in February 2021, there are a total of 9,209,834 and 35,443,993 patients eligible for linkage in those respective databases. Considering our study population definition (e.g. 'up to standard' data and 'acceptable patients') and design, we expect that our study will be sufficiently powered using the data of approximately 3 million acceptable patients from GOLD. We propose using GOLD as our primary dataset. Using the same study population definitions, we anticipate there being 11 million acceptable patients in the Aurum database. However, we will use Aurum as a secondary validation dataset limited to the ethnically-diverse East London population, which includes seven Clinical Commissioning Groups (Tower Hamlets, City and Hackney, Newham, Waltham Forest, Barking and Dagenham, Redbridge and Havering), being a smaller dataset required, according to the CPRD collected-data from those practices.

J. Planned use of linked data (if applicable):

This study will use individual level anonymised data within linked primary and secondary care records. The use of linked data from the NHS Hospital Episode Statistics (HES) for admitted-patient care will allow us to identify hospital-

coded conditions that contribute to multimorbidity, to characterise pregnancies, and to add information on health service utilisation, such as number of hospital admissions per year, and to obtain a higher completeness of ethnicity data (a core variable to our study). Moreover, the use of HES data will enable us to undertake validation of diagnosis across primary and secondary care (e.g. HIV and cancer). The linkage to the Office for National Statistics Death registries will allow us to know the cause of death and therefore understand the most common cause of death according to the patterns of diseases' clusters.

Linked area-based measures of deprivation will include the Indices of Multiple Deprivation (IMD) for both patient and practice level. The IMD is an estimate of socioeconomic status that play an important role to multimorbidity. We will use patient level IMD where available. For patients without linked patient level IMD, we will use practice level IMD as a proxy.

We expect that the identification of specific multimorbidity clusters and their different trajectories across ethnic groups will help identify groups of patients who may share similar prognosis and health care needs. Our study findings will support clinicians in the stratification of clinical care and will contribute to preventive strategies across multimorbid conditions. We expect that our study outputs may help improve patient care either directly, e.g. by recognising a common cluster of multimorbidity linked to a specific disease, or indirectly providing scientific support to the inclusion of multimorbidity in clinical practice guidelines and public health policies.

K. Definition of the Study population

Our study population comprises all acceptable patients permanently registered in any English General Practice that meet 'up to standard' CPRD GOLD data quality criteria between January 1, 2010 and December 31, 2020 and who ever had a recorded multimorbidity condition during this time frame. Patients are labelled as 'acceptable' if they meet acceptable data quality criteria for use in research.

With regards to the observational cohort design, our cohort will include all individuals aged 16 and over contributing data between January 1, 2010 and January 1, 2021. Each patient will have their Index Date, when the presence of multimorbidity will be defined. All included participants will be required to have 1 year of registration before the Index Date to maximise the record of prevalent cases across at least 12 months of registration. The patients will be followed from their Index Date until the earliest of dying, leaving the CPRD database, or the end of the follow-up period (1st Jan 2021).

We will use the CPRD Aurum as a validation data set, aiming to replicate our methods and validate our findings using the East London population, which comprises seven Clinical Commissioning Groups (Tower Hamlets, City and Hackney, Newham, Waltham Forest, Barking and Dagenham, Redbridge and Havering). The population specifications for Aurum will follow the same specifications as GOLD, i.e. in defining 'acceptable patients'. Considering that there is duplicate historical data for practices that have contributed data for both CPRD GOLD and Aurum, we expect CPRD to provide a bridging file to identify the overlapping practices and dates.

Our study population will be limited to those who are eligible for linkage.

L. Selection of comparison group(s) or controls

In our observational cohort analysis, we will consider as case any patient who have multimorbidity (two or more long-term conditions listed in Appendix A). However, we will not have a comparison group since we aim to compare results across different levels of exposure rather than an unexposed versus exposed comparison.

M. Exposures, Outcomes and Covariates

Research Aim A)

This analysis does not have a specific exposure since it is a descriptive analysis of clusters of multimorbidity. Nevertheless, the clusters will be closely investigated according to covariables such as sex, age group, ethnicity (White, Black African and Caribbean, and south Asian) and socioeconomic deprivation levels (using the Index of Multiple Deprivation). The outcome of this analysis are the clusters of multimorbidity, defined using a list of 220 conditions (Appendix 1).

Research Aims B and C)

Our research aims B and C will assess the trajectories of multimorbidity clusters of diseases and their relationship with prescribing. The main outcome of this analysis is the change of cluster the patients belong to over time. We will also consider mortality (date and cause of death) as a secondary outcome of the observational cohort. We will evaluate whether the progression of multimorbidity trajectories is predicted by known risk factors (e.g. high socioeconomic deprivation, smoking status, body mass index and clinical measures), and how it varies across different ethnic groups (White, Black African and Caribbean, and south Asian). Additionally, we have selected some indicators of health-seeking/risk behaviours, health care utilisation and life course variables. These variables are as follows:

1. Primary exposures:

(a) Socioeconomic variables

- Index of Multiple Deprivation (individual and practice level)
- Ethnicity

(b) Health seeking/risk behaviours

- Smoking status
- Alcohol consumption
- Influenza vaccination

(c) Prescribing

- Continuous prescriptions: drugs within the same class prescribed 3 times or more per year: name, class and date of prescription.
- Polypharmacy: we will construct a derived variable, and expect to define this as the simultaneous use of five or more continuous prescriptions.

(d) Clinical measures

- LDL-C (Low-density lipoprotein-Cholesterol)
- Total Cholesterol
- Triglycerides
- HDL-C (High-density lipoprotein-Cholesterol)
- HbA1c (Glycosylated haemoglobin, type A1C)
- Serum creatinine
- Estimated Glomerular filtration rate (eGFR)
- Urine albumin to creatinine ratio (ACR)
- Height
- Weight
- Blood pressure

2. Secondary exposures:

(a) Life events

- Pregnancy (defined by 3rd trimester delivery – live birth or stillbirth)
- Hypertensive disorders of pregnancy
- Gestational diabetes
- Menopause
- Lives in a care home
- Date and cause of death

(b) Health service utilisation

- Number of hospital admissions per year
- Number of primary care consultations per year

A code set has been built for all multimorbidity conditions by comparing published code sets from previous studies (2,3) and those built by CPRD for use with their GOLD data. All code sets are being clinically revised using CPRD GOLD code browser.

Considering that CPRD GOLD and Aurum have different structures, in order to use the same codes we plan to map the code sets by matching the Read codes and dm +d codes across CPRD GOLD and Aurum medical and product dictionaries to identify the same set of conditions and prescribing, respectively.

N. Data/ Statistical Analysis

Research Aim A)
We will estimate the prevalence of each condition included in our consensus-built list of 220 conditions across sex, age groups and ethnic groups (White, Black African and Caribbean, and south Asian). We will also describe the presence of multimorbidity (≥ 2 conditions) across different sociodemographic characteristics (age, sex, ethnic groups and deprivation level).

We intend to apply unsupervised machine learning techniques to identify clusters of multimorbidity across an unselected population, and within specific ethnic groups. Recent research suggests that unsupervised learning techniques have potential to identify novel patterns and relationships from electronic health records and reveal latent disease clusters and patient subgroups (16). We will potentially use the overlapping technique, which allows each disease to belong to two or more clusters that present separate degrees of membership. The advantage of using this type of clustering is that the diseases on the boundaries between several clusters are not forced to fully belong to a single cluster, but rather are assigned membership degrees between 0 and 1 indicating their partial membership (17,18). We may apply a prevalence-based revision of the conditions included in our multimorbidity clusters (as used by Kuan and colleagues (2) who set a prevalence cut off of 0.01% in their data-driven analysis) if there is concern that very low prevalence conditions are subject to reporting bias or poor data quality.

We will describe the patterns of multimorbidity by calculating the frequencies and percentages of conditions within each cluster, and diseases will be assigned to the cluster in which they present the highest membership value. The membership value will be estimated using the ratio between the disease prevalence within the cluster by the disease prevalence in the overall population (19). We will examine how clustering varies in the context of more prevalent conditions (e.g. diabetes, hypertension, depression) compared to less prevalent conditions (e.g. HIV and cancer).

We will use the CPRD Aurum as a validation data set, aiming to replicate our methods and validate our findings in an ethnically-diverse East London population.

Research Aims B)
To characterise the trajectory of multimorbidity clusters across the life course, we will identify clusters of multimorbidity and trace their evolution from single index conditions, and their trajectories across the lifecourse, including to death, over ten years. We anticipate focusing this analysis on up to 8 multimorbidity clusters, selected to ensure we generate new knowledge, e.g. clusters which include less-studied conditions such as cancer or HIV, as well as those where there is variation by ethnicity. We will follow the individuals within the selected clusters to identify the acquisition of new conditions and potentially changes in the cluster they belong to over time. Our analysis will consider how these trajectories to multimorbidity vary according to different 'index conditions', e.g. diabetes, HIV and cancer.

The assessment of the sequence of multimorbidity clusters an individual belongs to over the years will allow us to understand their disease trajectory over the period they were under observation and at different points in time. The multimorbidity cluster trajectories assessment will take into account the individuals' socioeconomic characteristics, potential risk factors and time dependent variables (e.g. clinical measurements, health seeking behaviours, health care utilisation, and life events).

We will identify individuals who share similar patterns of multimorbidity using clustering algorithms at different points in time. We aim to assess at least three points in time, with a ~five year interval between them (e.g. Jan 2011, Jan 2016 and Dec 2020). This time window will be subject to revision according to the available follow-up time in years of our study population. To evaluate the multimorbidity cluster trajectories, each individual will be assigned to the cluster with the highest membership degree in each point in time. We will be able to identify how the multimorbidity cluster changes in individuals from different age groups, sex and ethnicity.

We will estimate the association between the multimorbidity clusters and the risk to mortality in each follow-up period using Cox models adjusted by socioeconomic characteristics (age, sex, IMD) and risk factors (clinical measurements, health seeking/risk behaviours, health care utilisation, and life events) some of which are time dependent variables (e.g. BMI, blood pressure, lipids, HbA1c).

Research Aim C)

We will study prescribing and its relationship with multimorbidity clusters, and we anticipate focusing on the clusters under study in Research Aim B). We will investigate continuous prescriptions, defined as a prescription from within the same class repeated up to 3 times per year (allowing for drug switching between classes, and stockpiling between prescriptions). This will allow us to define polypharmacy, using the commonly used definition (the simultaneous, continuous prescription of 5 or more medications) but also to develop our own characterisation of the association between prescribing patterns and multimorbidity, e.g. the role of specific drugs. We will use several methodologies spanning machine learning and artificial intelligence which have not yet been used commonly in this field. The methods we expect to use overlap (in part) with our clustering approaches, and include: Association Rule Mining (ARM), or market-basket analysis; Latent Class Analysis (LCA), traditional iterative clustering methods (k-means, Hierarchical Cluster Analysis), as well as matrix factorisation methods. We will initially focus on ARM and factorisation methods, which are complementary and have proved successful in recent multimorbidity studies.

O. Plan for addressing confounding

To address confounding we selected a priori confounders that according to the literature are associated with an increased risk for multimorbidity. The main strategy to address confounding will be the analysis stratification according to sex, age group and ethnic groups (White, Black African and Caribbean, and south Asian).

P. Plans for addressing missing data

We will assume that the individuals who do not have any recorded code do not have the disease (for further discussion, please also see Section T. Limitations on the study design). We will include patients who have complete data for the stratification variables (e.g. sex, age and ethnic groups) and who have linked measures of deprivation. Given the high levels of missingness for some covariates, i.e. body mass index, total cholesterol, and alcohol intake specific statistical techniques will be applied to handle missing-not-at-random data, e.g. sensitivity analysis and multiple imputation techniques where appropriate.

The design of the study assumes that patients who do not meet the criteria according to the coding algorithm do not have the disease. This may introduce biases due to under-reporting or misreporting. Please see section O for further details.

In the main analyses, where patients will be stratified by age and sex, any individuals with missing data for either will be excluded. In addition, patients without data for ethnicity and deprivation will be excluded from sensitivity analyses.

Q. Patient or user group involvement

The proposed research is supported by the Public Patient Involvement (PPI) strategy that underpins the MRC and NIHR awards funding it. Across these studies, we have gained support of existing PPI groups in the prioritisation of our research and they have supported the need an approach being taken. It is worth noting that most PPI groups are predominantly single disease-focused and therefore multimorbidity is a new area for them. Our ongoing PPI strategy

across both of these grants will engage the public and patients throughout the research pathway, now from the conduct to dissemination of our findings. We will also receive support from the North Thames Applied Research Collaborative (ARC) with PPI activities.

R. Plans for disseminating and communicating study results

We will be guided by the MRC Communication and Engagement Strategy and NIHR INVOLVE guidance in all our communications activities, targeting the following groups.

Academic community: We will communicate our findings to a wider scientific community via presentations at national and international conferences and professional meetings across a range of audiences including genomics, clinical and health data communities. We will publish several high impact publications from this work, using preprint servers and open access journals in preference. We anticipate 'telling the story' of our research via our institutional websites, blogs (e.g. MRC Insight and The Conversation) and NIHR networks. The programme will be linked within the North East and North Cumbria and North London Applied Research Consortia (ARCs) (particularly the multimorbidity, ageing and frailty theme). The lead applicant is a member of a Diabetes UK strategic "Clinical Studies Group" shaping future research and policy in type 2 diabetes. She will use this position to influence future multimorbidity research and translation to clinical care related to diabetes. This research may identify areas for future research prioritisation, and if so, the research team would engage with research prioritisation processes, e.g. James Lind Alliance prioritisation, and submission of topic sources to NIHR.

Health and social care professionals, organisations and policymakers: We anticipate our findings to have translational impact in the future. We will engage with health and social care professionals using national and local networks and professional interest groups to discuss our results. As there is no unifying clinical body leading on multimorbidity, we will use a broad range of speciality- and discipline-specific organisations, e.g. Diabetes UK, British Heart Foundation, Royal Colleges. We will also engage with health and social care commissioners and policy makers who are increasingly involved in delivering and commissioning new models of care for multimorbidity.

S. Conflict of interest statement

The authors do not have any conflict of interest.

T. Limitations of the study design, data sources, and analytic methods

There are a few limitations to our study design, analytic methods and use of data sources:

- Adding only ‘acceptable patients’ could potentially introduce a selection bias to our study. Acceptable patients might underrepresent a group of patients that are difficult to engage, e.g. those who may often change GP practice but who are also multimorbid, and who might be from minority ethnic groups. Similarly, including only ‘up to standard’ data will assure high quality data, but could introduce selection bias by including general practices with better quality of, and access to, healthcare and access to primary healthcare. However, including only ‘up to standard’ data from acceptable patients will ensure high quality data, an essential aspect to work both with longitudinal analysis. This limitation will be addressed in our study discussion.
- The cluster analysis is dependent on the measures (e.g. variables/conditions) used to characterise the objects (e.g. individuals). Despite our comprehensive list of conditions (we will expand on the existing ~40 conditions commonly used in most of the UK’s multimorbidity studies due to its data quality (mostly incentivised via QOF) to include a larger set of clinically-relevant conditions (n = 220)) our clustering solution might not be generalised as it is dependent upon the included conditions (e.g. the addition or exclusion of variables/conditions influences the clustering solution we get from the analysis). However, it will cover a broader set of conditions, compared to various studies on multimorbidity. We are aware that recording of non-incentivised conditions will be less comprehensive and therefore this may introduce bias from coding frequency/accuracy.
- We have included a few sexually transmitted conditions in our study (e.g. HIV, chronic hepatitis) that might be under-reported in the CPRD linked dataset for reasons of confidentiality, also given that in the UK, most consultations involving sexually transmitted infections are diagnosed and treated at sexual health service centres (2).
- Our study might be subject to misclassification due to unrecorded, miscoded and undiagnosed diseases. Likewise, a patient with no disease diagnoses record will be considered as disease free, even though the lack

of diagnosis code might as well include patients with morbidity, but missing diagnosis record. However, our study is limited by the quality of data recording.

- We are aware of the limitations of using primary care prescribing data in our analyses. In CPRD we will know when prescriptions are issued but not whether they are dispensed– this may make it difficult to explore questions around adherence (though we can assume to a certain extent that people who receive repeat prescriptions are taking their medications regularly). Additionally, we will not receive records of over-the-counter medication or in-hospital prescribing, however this is more typically short-term and therefore less likely to contribute polypharmacy.

U. References

1. Denaxas S, Gonzalez-Izquierdo A, Direk K, Fitzpatrick NK, Fatemifar G, Banerjee A, et al. UK phenomics platform for developing and validating electronic health record phenotypes: CALIBER. J Am Med Inform Assoc. 2019 Jul 22;26(12):1545–59.

2. Kuan V, Denaxas S, Gonzalez-Izquierdo A, Direk K, Bhatti O, Husain S, et al. A chronological map of 308 physical and mental health conditions from 4 million individuals in the English National Health Service. The Lancet Digital Health. 2019 Jun 1;1(2):e63–77.

3. Payne RA, Mendonca SC, Elliott MN, Saunders CL, Edwards DA, Marshall M, et al. Development and validation of the Cambridge Multimorbidity Score. CMAJ. 2020 Feb 3;192(5):E107–14.

4. Multimorbidity: a priority for global health research | The Academy of Medical Sciences [Internet]. [cited 2020 Jun 10]. Available from: https://acmedsci.ac.uk/policy/policy-projects/multimorbidity

5. Barnett K, Mercer SW, Norbury M, Watt G, Wyke S, Guthrie B. Epidemiology of multimorbidity and implications for health care, research, and medical education: a cross-sectional study. Lancet. 2012 Jul 7;380(9836):37–43.

6. Mathur R, Hull SA, Badrick E, Robson J. Cardiovascular multimorbidity: the effect of ethnicity on prevalence and risk factor management. Br J Gen Pract. 2011 May 1;61(586):e262–70.

7. Ashworth M, Durbaba S, Whitney D, Crompton J, Wright M, Dodhia H. Journey to multimorbidity: longitudinal analysis exploring cardiovascular risk factors and sociodemographic determinants in an urban setting. BMJ Open. 2019 Dec 1;9(12):e031649.

8. Cassell A, Edwards D, Harshfield A, Rhodes K, Brimicombe J, Payne R, et al. The epidemiology of multimorbidity in primary care: a retrospective cohort study. Br J Gen Pract. 2018;68(669):e245–51.

9. De Francesco D, Verboeket SO, Underwood J, Bagkeris E, Wit FW, Mallon PWG, et al. Patterns of Co-occurring Comorbidities in People Living With HIV. Open Forum Infectious Diseases [Internet]. 2018 Nov 1 [cited 2021 Feb 22];5(ofy272). Available from: https://doi.org/10.1093/ofid/ofy272

10. Singh-Manoux A, Fayosse A, Sabia S, Tabak A, Shipley M, Dugravot A, et al. Clinical, socioeconomic, and behavioural factors at age 50 years and risk of cardiometabolic multimorbidity and mortality: A cohort study. PLOS Medicine. 2018 May 21;15(5):e1002571.

11. Zhu Y, Edwards D, Mant J, Payne RA, Kiddle S. Characteristics, service use and mortality of clusters of multimorbid patients in England: a population-based study. BMC Med [Internet]. 2020 Apr 10 [cited 2020 Jun 7];18. Available from: https://www.ncbi.nlm.nih.gov/pmc/articles/PMC7147068/

12. Menditto E, Gimeno Miguel A, Moreno Juste A, Poblador Plou B, Aza Pascual-Salcedo M, Orlando V, et al. Patterns of multimorbidity and polypharmacy in young and adult population: Systematic associations among chronic diseases and drugs using factor analysis. PLoS One. 2019;14(2):e0210701.

13. Pourhoseingholi MA, Vahedi M, Rahimzadeh M. Sample size calculation in medical studies. Gastroenterol Hepatol Bed Bench. 2013;6(1):14–7.

14. Salisbury C, Johnson L, Purdy S, Valderas JM, Montgomery AA. Epidemiology and impact of multimorbidity in

primary care: a retrospective cohort study. Br J Gen Pract. 2011 Jan 1;61(582):e12–21.

15. Kuan V, Fraser HC, Hingorani M, Denaxas S, Gonzalez-Izquierdo A, Direk K, et al. Data-driven identification of ageing-related diseases from electronic health records. Scientific Reports. 2021 Feb 3;11(1):2938.

16. Wang Y, Zhao Y, Therneau TM, Atkinson EJ, Tafti AP, Zhang N, et al. Unsupervised machine learning for the discovery of latent disease clusters and patient subgroups using electronic health records. Journal of Biomedical Informatics. 2020 Feb 1;102:103364.

17. Bezdek JC, Ehrlich R, Full W. FCM: The fuzzy c-means clustering algorithm. Computers & Geosciences. 1984 Jan 1;10(2):191–203.

18. Bora D, Gupta D. A Comparative study Between Fuzzy Clustering Algorithm and Hard Clustering Algorithm. International Journal of Computer Trends and Technology. 2014 Apr 24;10:108–13.

19. Violán C, Foguet-Boreu Q, Fernández-Bertolín S, Guisado-Clavero M, Cabrera-Bean M, Formiga F, et al. Soft clustering using real-world data for the identification of multimorbidity patterns in an elderly population: cross-sectional study in a Mediterranean population. BMJ Open [Internet]. 2019 Aug 30 [cited 2020 Jun 7];9(8). Available from: https://www.ncbi.nlm.nih.gov/pmc/articles/PMC6719769/

List of Appendices

Appendix 1 – List of long-term conditions
Appendix 2 – Sample Code sets for selecting comorbidities

Grant ID *(optional)*

NIHR202635
MR/S027297/1

Amendment – 27 August 2021

J. Planned use of linked data (if applicable):

Our study will use individual-level anonymised data from the Pregnancy Register database. The use of this database will allow us to identify the pregnancy episodes as well their start and end dates among our cohort of patients, considering that pregnancy is an important life-course event to our analysis of trajectories of multimorbidity.
