# Supplementary material for: Ethnic differences in early onset multimorbidity and associations with health service use, long-term prescribing, years of life lost, and mortality: A cross-sectional study using clustering in the UK Clinical Practice Research Datalink
Source: PLoS Med. 2023 Oct 27;20(10):e1004300. doi: 10.1371/journal.pmed.1004300 (PMC10610074; doi:10.1371/journal.pmed.1004300)
Supplement: S1 Text — (DOCX) [file pmed.1004300.s003.docx]

**S1 Text**

# **Selection of the Long-Term Conditions**

Following a two-round consensus exercise composed of ten clinicians from diverse specialities, 204 LTCs were included in our operational definition of multimorbidity from a base list of 311 LTCs, derived from existing high-quality studies and codelist resources studies [1–5]. The codelist revision, development and curation consisted of the following approaches:

(a) Comparison between existing codelists followed by clinical curation,

(b) Development of new codelists through relevant medical terms and,

(c) Cross-mapping of different code systems using revised codelists as a baseline.

We revised 263 codelists built on Read v2, 214 codelists built on ICD-10 (International Classification of Diseases, Tenth Revision), 13 codelists built on OPCS-4 (Classification of Interventions and Procedures version 4), and 2 codelists built on gemscript codes (prescribing codes).

Using the consensus exercise outlined, we extensively revised and selected codelists for 204 LTCs defined by 11,053 Read v2 codes, 2,594 ICD-10 codes, 747 OPCS-4 codes, and 3,829 gemscript codes. Detailed information on the codelist curation and development and all codelists used in our study can be found in the MULTIPLY-Initiative online repository. [6]

# **References**

1. Ashworth M, Durbaba S, Whitney D, Crompton J, Wright M, Dodhia H. Journey to multimorbidity: longitudinal analysis exploring cardiovascular risk factors and sociodemographic determinants in an urban setting. BMJ Open. 2019;9: e031649. doi:10.1136/bmjopen-2019-031649

2. Barnett K, Mercer SW, Norbury M, Watt G, Wyke S, Guthrie B. Epidemiology of multimorbidity and implications for health care, research, and medical education: a cross-sectional study. The Lancet. 2012;380: 37–43. doi:10.1016/S0140-6736(12)60240-2

3. Calderón-Larrañaga A, Vetrano DL, Onder G, Gimeno-Feliu LA, Coscollar-Santaliestra C, Carfí A, et al. Assessing and Measuring Chronic Multimorbidity in the Older Population: A Proposal for Its Operationalization. J Gerontol A Biol Sci Med Sci. 2017;72: 1417–1423. doi:10.1093/gerona/glw233

4. Kuan V, Denaxas S, Gonzalez-Izquierdo A, Direk K, Bhatti O, Husain S, et al. A chronological map of 308 physical and mental health conditions from 4 million individuals in the English National Health Service. Lancet Digit Health. 2019;1: e63–e77. doi:10.1016/S2589-7500(19)30012-3

5. Payne RA, Mendonca SC, Elliott MN, Saunders CL, Edwards DA, Marshall M, et al. Development and validation of the Cambridge Multimorbidity Score. CMAJ. 2020;192: E107–E114. doi:10.1503/cmaj.190757

6. Eto F, Samuel M, Finer S. MULTIPLY-Initiative. 2023. Available: https://github.com/Fabiola-Eto/MULTIPLY-Initiative
