# Supplementary material for: Ethnic differences in early onset multimorbidity and associations with health service use, long-term prescribing, years of life lost, and mortality: A cross-sectional study using clustering in the UK Clinical Practice Research Datalink
Source: PLoS Med. 2023 Oct 27;20(10):e1004300. doi: 10.1371/journal.pmed.1004300 (PMC10610074; doi:10.1371/journal.pmed.1004300)
Supplement: S2 Text — (DOCX) [file pmed.1004300.s004.docx]

**S2 Text**

# **Fit statistics for the model selection**

We obtained and compared the fit statistics for each k-class model, which along with clinical judgement – on the clusters that besides being clinically meaningful also captured heterogeneous LTCs – supported the selection of the optimal number of latent classes. We obtained the bootstrapped likelihood ratio test (BLRT) which indicates if a model with k classes is statistically better than a model with k–1 class upon a provided p-value [1]. We evaluated the Bayesian Information Criterion (BIC), the Sample-size Adjusted Bayesian Information Criterion (SABIC), and the likelihood ratio test (LRT) to compare competing models. They inform how much a model is improved by an additional class. Lower BIC, SABIC and LRT values indicate a better-fit model. The entropy was assessed to verify the model quality since it indicates how accurately the model defines classes. In general, a value closer to 1 is desirable, although there is no agreed-upon cutoff criterion for entropy [2].

To select the models with the best class solution we verified the fit statistics (BIC, SABIC, likelihood ratio and entropy) for each model along with the clinical judgement of the most meaningful clusters among a couple of candidates for the best solution.

The BIC is considered the most reliable indicator of model fit [1]. Although, in practice, it is common that the BIC and SABIC (BIC adjusted for the sample size) decrease as you add classes to the model. Therefore, we opt for the most parsimonious model when evaluating these indexes. The likelihood ratio shows the difference in likelihood between the fitted model (e.g. *k* model being tested) and the *k-1* model, where *k* is the number of classes. Lower values indicate a more parsimonious model. The entropy assessed how accurately the classes classify individuals. In general, a value closer to 1 is desirable, although there is no agreed-upon cut-off criterion for entropy [2]. In practice, we inspected the plots for an “elbow” or a point where we do not see much improvement in the model fit (e.g. small decreases in the fit statistics for each additional class).

A 4-class model for the White population (Fig AA, Table A) and a 3-class model for both the South Asian and Black African/Caribbean (Figure AB and Fig AC, respectively, Table A) were selected as the best solution given that only small improvements in the fit indexes for additional classes can be seen from upper classes. Additionally, the respective classes show an improvement in the model classification (entropy) compared to their neighbour models.

**(A) White**

**
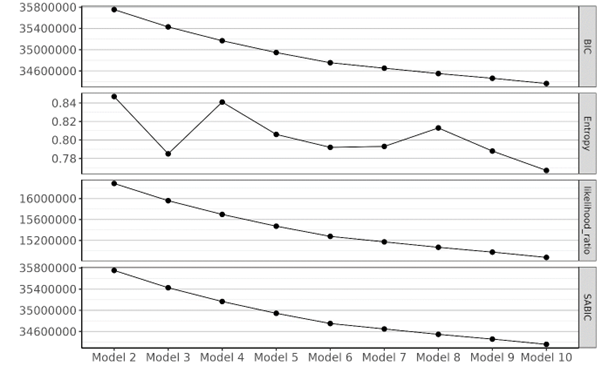
**

**(B) South Asian**

**
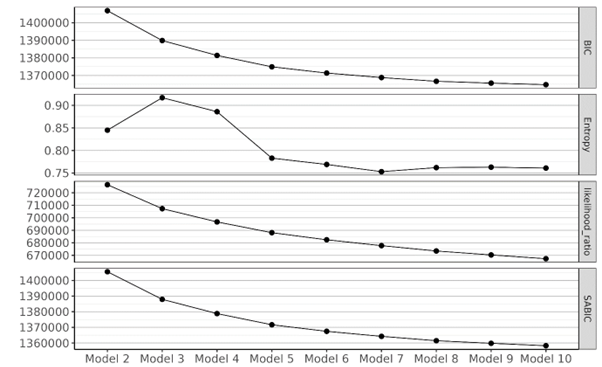
**

(Fig A continues on next page)

**(C) Black African/Caribbean**


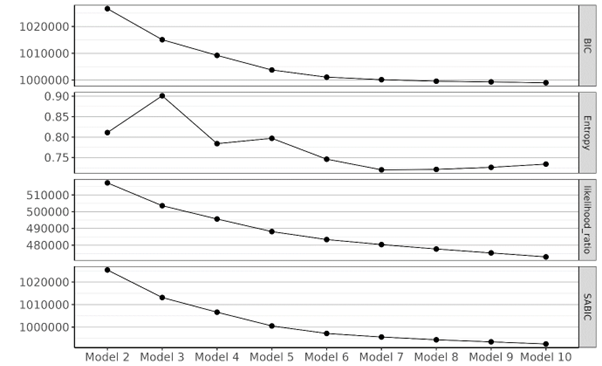


**Fig A.** **Fit statistics for the latent class models for the three ethnic groups.**

**Table A.** Fit statistics for the latent class models according to ethnic groups.

| **Model** | **log_likelihood** | **resid.df** | **BIC** | **AIC** | **Chisq** | **SABIC** | **likelihood_ratio** | **error_prior** | **error_post** | **Entropy** | **Group** |
| --- | --- | --- | --- | --- | --- | --- | --- | --- | --- | --- | --- |
| Model 2 | -701317.3213 | 33508 | 1406880.309 | 1403448.643 | 2.92E+45 | 1405587 | 726451.9576 | 0.487175668 | 0.075752801 | 0.845 | asian |
| Model 3 | -691750.5748 | 33304 | 1389874.865 | 1384723.15 | 6.47E+44 | 1387933 | 707318.4647 | 0.796990011 | 0.066392919 | 0.917 | asian |
| Model 4 | -686450.0011 | 33100 | 1381401.766 | 1374530.002 | 3.15E+42 | 1378812 | 696717.3172 | 0.970368649 | 0.110215816 | 0.886 | asian |
| Model 5 | -682140.9822 | 32896 | 1374911.778 | 1366319.964 | 2.08E+40 | 1371673 | 688099.2795 | 1.37247251 | 0.297731198 | 0.783 | asian |
| Model 6 | -679304.0053 | 32692 | 1371365.873 | 1361054.011 | 1.26E+40 | 1367479 | 682425.3257 | 1.592144656 | 0.368201414 | 0.769 | asian |
| Model 7 | -676955.3779 | 32488 | 1368796.667 | 1356764.756 | 1.37E+37 | 1364262 | 677728.0708 | 1.76007319 | 0.43419922 | 0.753 | asian |
| Model 8 | -674825.1655 | 32284 | 1366664.291 | 1352912.331 | 5.87E+36 | 1361481 | 673467.6461 | 1.884758204 | 0.447879329 | 0.762 | asian |
| Model 9 | -673244.9008 | 32080 | 1365631.811 | 1350159.802 | 4.78E+37 | 1359800 | 670307.1164 | 1.950600018 | 0.462931579 | 0.763 | asian |
| Model 10 | -671739.8294 | 31876 | 1364749.717 | 1347557.659 | 1.64E+36 | 1358270 | 667296.9738 | 2.042375455 | 0.488299208 | 0.761 | asian |
| Model 2 | -511263.327 | 25641 | 1026664.906 | 1023340.654 | 5.51E+47 | 1025371 | 517243.0573 | 0.455722844 | 0.08632568 | 0.811 | black |
| Model 3 | -504431.1784 | 25437 | 1015074.819 | 1010084.357 | 8.43E+46 | 1013133 | 503578.7601 | 0.742674141 | 0.073690574 | 0.901 | black |
| Model 4 | -500461.1686 | 25233 | 1009209.01 | 1002552.337 | 3.14E+46 | 1006619 | 495638.7405 | 1.147301567 | 0.247630766 | 0.784 | black |
| Model 5 | -496697.1553 | 25029 | 1003755.193 | 995432.3105 | 1.43E+45 | 1000517 | 488110.7137 | 1.266181127 | 0.256851823 | 0.797 | black |
| Model 6 | -494317.4525 | 24825 | 1001069.997 | 991080.9049 | 1.10E+44 | 997183.3 | 483351.3082 | 1.585033643 | 0.402742583 | 0.746 | black |
| Model 7 | -492812.2738 | 24621 | 1000133.85 | 988478.5475 | 8.31E+42 | 995598.9 | 480340.9507 | 1.803276012 | 0.50546652 | 0.72 | black |
| Model 8 | -491486.4095 | 24417 | 999556.3315 | 986234.8189 | 6.63E+44 | 994373.1 | 477689.2221 | 1.927614117 | 0.538056189 | 0.721 | black |
| Model 9 | -490320.697 | 24213 | 999299.1167 | 984311.394 | 2.41E+40 | 993467.5 | 475357.7972 | 1.952536796 | 0.535075245 | 0.726 | black |
| Model 10 | -489129.1085 | 24009 | 998990.1498 | 982336.2171 | 3.13E+40 | 992510.3 | 472974.6203 | 2.066843641 | 0.550229807 | 0.734 | black |
| Model 2 | -17875002.03 | 777497 | 35755551.88 | 35750822.06 | 2.15E+154 | 35754252 | 16289294.86 | 0.537737544 | 0.082073425 | 0.847 | white |
| Model 3 | -17710483.25 | 777292 | 35429295.02 | 35422194.51 | 8.42E+146 | 35427344 | 15960257.31 | 0.835281984 | 0.179459958 | 0.785 | white |
| Model 4 | -17578966.11 | 777087 | 35169041.43 | 35159570.22 | 3.54E+146 | 35166439 | 15697223.03 | 1.011667444 | 0.161118418 | 0.841 | white |
| Model 5 | -17466348.58 | 776882 | 34946587.06 | 34934745.16 | 4.70E+144 | 34943333 | 15471987.96 | 1.268809142 | 0.245637368 | 0.806 | white |
| Model 6 | -17368956.37 | 776677 | 34754583.34 | 34740370.74 | 5.02E+136 | 34750678 | 15277203.55 | 1.481724293 | 0.308854985 | 0.792 | white |
| Model 7 | -17316274.92 | 776472 | 34652001.14 | 34635417.85 | 4.07E+137 | 34647444 | 15171840.65 | 1.579234059 | 0.326786088 | 0.793 | white |
| Model 8 | -17264261.5 | 776267 | 34550755 | 34531801.01 | 4.33E+135 | 34545546 | 15067813.81 | 1.651448573 | 0.309527659 | 0.813 | white |
| Model 9 | -17218389.28 | 776062 | 34461791.24 | 34440466.56 | 7.58E+135 | 34455931 | 14976069.37 | 1.817802696 | 0.384675542 | 0.788 | white |
| Model 10 | -17167755.81 | 775857 | 34363305 | 34339609.63 | 3.84E+134 | 34356793 | 14874802.43 | 2.014486824 | 0.469591847 | 0.767 | white |

# **References**

1. Nylund KL, Asparouhov T, Muthén BO. Deciding on the Number of Classes in Latent Class Analysis and Growth Mixture Modeling: A Monte Carlo Simulation Study. Struct Equ Model Multidiscip J. 2007;14: 535–569. doi:10.1080/10705510701575396

2. Muthén B. What is a good value of entropy? 2008 [cited 12 Dec 2022]. Available: http://www.statmodel.com/discussion/messages/13/2562.html?1487458497
