# Supplementary material for: Ethnic differences in early onset multimorbidity and associations with health service use, long-term prescribing, years of life lost, and mortality: A cross-sectional study using clustering in the UK Clinical Practice Research Datalink
Source: PLoS Med. 2023 Oct 27;20(10):e1004300. doi: 10.1371/journal.pmed.1004300 (PMC10610074; doi:10.1371/journal.pmed.1004300)
Supplement: S1 Table — (DOCX) [file pmed.1004300.s005.docx]

| **S1 Table. Prevalence of the 204 long-term conditions per 100 according to clusters within each ethnic group (White n = 777,906; South Asian n = 33,915; and Black African/Caribbean n = 26,048). Population with early onset of multimorbidity (16 to 39 years old).** | | | | | | | | | | | |  |
| --- | --- | --- | --- | --- | --- | --- | --- | --- | --- | --- | --- | --- |
| **Long-term condition** | **White** | | | | **South Asian** | | | | **Black African/Caribbean** | | |  |
|  | **Cluster 1** | **Cluster 2** | **Cluster 3** | **Cluster 4** | **Cluster 1** | **Cluster 2** | **Cluster 3** | **Cluster 1** | | **Cluster 2** | **Cluster 3** | |
| ADHD and hyperkinetic disorders | 0.2 | 0.7 | 0.3 | 0.3 | 0 | 0.1 | 0.1 | 0 | | 0.2 | 0.3 | |
| Adrenal insufficiency and Addison's disease | 0.1 | 0.1 | 0.3 | 0.9 | 0 | 0.1 | 0.5 | 0.1 | | 0.1 | 0.7 | |
| Alcohol dependence and related disease | 3 | 7.2 | 6.2 | 18.2 | 1.2 | 3.1 | 4.4 | 1.2 | | 3.7 | 6.8 | |
| Allergic and chronic rhinitis | 17.9 | 18.9 | 30.9 | 20.7 | 20.4 | 25.7 | 39.2 | 24.1 | | 27.3 | 37.8 | |
| Alopecia areata and scarring alopecia | 0.5 | 0.6 | 0.8 | 0.8 | 1.1 | 1.6 | 2.1 | 1 | | 0.8 | 1.4 | |
| Ankylosing spondylitis | 0.2 | 0.3 | 0.8 | 1.2 | 0.1 | 0.2 | 0.7 | 0 | | 0.1 | 0.2 | |
| Anxiety and phobia | 23.9 | 28 | 53.2 | 41.4 | 10.5 | 14 | 32.1 | 10.3 | | 14.1 | 28.6 | |
| Aortic aneurysm | 0 | 0 | 0.1 | 2.6 | 0 | 0 | 0.6 | 0 | | 0 | 0.4 | |
| Aplastic anaemias | 0.1 | 0.1 | 0.1 | 1.5 | 0.1 | 0.1 | 0.9 | 0.1 | | 0.2 | 1.2 | |
| Asbestosis | 0 | 0 | 0 | 0.6 | 0 | 0 | 0 | 0 | | 0 | 0 | |
| Asthma | 18.1 | 22.1 | 32.1 | 31.5 | 12 | 17.7 | 32.4 | 12.6 | | 16.7 | 29.8 | |
| Atrial fibrillation and flutter | 0.6 | 0.7 | 1.4 | 21.1 | 0.1 | 0.2 | 4.6 | 0.1 | | 0.4 | 5.7 | |
| Autism and Asperger's syndrome | 0.1 | 0.5 | 0.3 | 0.5 | 0 | 0.2 | 0.2 | 0 | | 0.4 | 0.5 | |
| Autoimmune liver disease | 0 | 0 | 0.1 | 0.7 | 0 | 0.1 | 0.6 | 0 | | 0.1 | 0.4 | |
| Barrett's oesophagus | 0.2 | 0.2 | 1.7 | 3.2 | 0 | 0.1 | 0.9 | 0 | | 0 | 0.5 | |
| Bipolar affective disorder and mania | 0.6 | 1.5 | 3 | 4.1 | 0.4 | 1 | 2.4 | 0.5 | | 2 | 3.7 | |
| Blistering autoimmune skin conditions | 0 | 0.1 | 0.1 | 0.4 | 0 | 0.1 | 0.3 | 0.1 | | 0.1 | 0.2 | |
| Bronchiectasis | 0.3 | 0.2 | 1 | 4.3 | 0.2 | 0.2 | 2 | 0.1 | | 0.2 | 1.7 | |
| Cardiac conduction defects | 0.4 | 0.5 | 0.8 | 8.9 | 0.2 | 0.3 | 3.8 | 0.1 | | 0.4 | 3.4 | |
| Cardiomyopathy other | 0.1 | 0.1 | 0.1 | 3.8 | 0.1 | 0.1 | 1.6 | 0.1 | | 0.2 | 3.5 | |
| Cerebral Palsy | 0 | 0.2 | 0.2 | 0.5 | 0 | 0.1 | 0.1 | 0 | | 0.1 | 0.4 | |
| Cerebrovascular disease | 0.7 | 0.6 | 2.3 | 14.7 | 0.2 | 0.4 | 6.5 | 0.4 | | 0.5 | 7.3 | |
| Cervical carcinoma in situ | 5.3 | 4.4 | 5.8 | 1.9 | 0.9 | 1 | 1.4 | 3.2 | | 3.7 | 3.5 | |
| Cholelithiasis | 5.7 | 4.1 | 14.8 | 14.2 | 3.9 | 3.6 | 12.4 | 4 | | 3.5 | 11 | |
| Chronic fatigue | 1.1 | 1.1 | 5.3 | 2 | 0.3 | 0.3 | 2.3 | 0.1 | | 0.3 | 1.6 | |
| Chronic Kidney Disease | 1.2 | 0.6 | 3.7 | 23.3 | 0.8 | 0.9 | 12.6 | 2.6 | | 1.5 | 18.9 | |
| Chronic obstructive pulmonary disease | 0.9 | 0.9 | 5.7 | 22.8 | 0.2 | 0.3 | 5.3 | 0.2 | | 0.4 | 4.9 | |
| Chronic sinusitis | 9.2 | 8.3 | 25.2 | 15.7 | 4.5 | 6.6 | 18.3 | 4.6 | | 4.7 | 14 | |
| Chronic ulcer of the skin | 2.2 | 2.5 | 5 | 20.1 | 0.7 | 1.7 | 8 | 0.8 | | 1.3 | 9 | |
| Chronic viral hepatitis | 0.5 | 0.8 | 0.6 | 3.1 | 0.9 | 1 | 2.2 | 2.1 | | 2.7 | 3.6 | |
| Coeliac disease | 0.5 | 0.6 | 1.4 | 1.1 | 0.6 | 0.8 | 1.3 | 0.1 | | 0.1 | 0.2 | |
| Collapsed vertebra | 0.1 | 0.1 | 0.3 | 2.1 | 0 | 0 | 0.6 | 0.1 | | 0 | 0.4 | |
| Congenital cardiac disease | 0.6 | 0.8 | 0.7 | 3.2 | 0.5 | 0.6 | 1.6 | 0.2 | | 0.4 | 2 | |
| Constipation | 13.5 | 10.5 | 33.6 | 37.3 | 19.6 | 18.4 | 43.2 | 18.4 | | 17 | 39 | |
| Coronary heart disease | 1.2 | 1.2 | 5.5 | 36 | 1.1 | 1.5 | 23.6 | 0.7 | | 0.7 | 14.1 | |
| Crohn's disease | 0.7 | 1 | 1.9 | 2 | 0.6 | 0.9 | 1.9 | 0.2 | | 0.4 | 1.1 | |
| Cystic Fibrosis | 0.1 | 0 | 0.1 | 0.2 | 0 | 0 | 0.1 | 0 | | 0 | 0.2 | |
| Cystic renal disease | 0.1 | 0.1 | 0.1 | 0.8 | 0 | 0.1 | 0.5 | 0.1 | | 0.1 | 0.6 | |
| Dementia | 0.1 | 0.1 | 0.4 | 7.1 | 0 | 0 | 1.7 | 0.1 | | 0.1 | 1.6 | |
| Depression | 35.8 | 40.9 | 72 | 58.8 | 17.1 | 21.1 | 48.9 | 19.1 | | 25.5 | 49.2 | |
| Dermatitis (atopic/contact/other/unspecified) | 27.3 | 26.1 | 40 | 34.1 | 28.4 | 30.8 | 48.2 | 21.3 | | 23.3 | 35.4 | |
| Diabetic eye disease | 0.6 | 0.7 | 1.2 | 12.9 | 0.8 | 1 | 14.2 | 1.2 | | 0.6 | 11.2 | |
| Diabetic neurological complications | 0 | 0 | 0.1 | 5.5 | 0 | 0 | 3 | 0.1 | | 0 | 2.6 | |
| Disorders of autonomic nervous system | 0.2 | 0.3 | 1.3 | 2.7 | 0.1 | 0.2 | 1.9 | 0.1 | | 0.1 | 1.7 | |
| Diverticular disease of intestine | 2.6 | 1.7 | 11.5 | 20.5 | 0.5 | 0.6 | 5.9 | 1.2 | | 0.7 | 8.2 | |
| Down's syndrome | 0.1 | 0.1 | 0.1 | 0.2 | 0.1 | 0 | 0.2 | 0 | | 0 | 0.2 | |
| Dysmenorrhoea | 10.8 | 5.8 | 17 | 2.8 | 9.9 | 6.2 | 12.4 | 14.9 | | 8.1 | 14.2 | |
| Eating disorders | 1.8 | 2 | 3.8 | 3.6 | 0.7 | 1.1 | 2.4 | 0.7 | | 1.1 | 2.7 | |
| End stage renal disease | 0 | 0.1 | 0 | 4 | 0 | 0.1 | 3.7 | 0.2 | | 0.1 | 6.1 | |
| Endometriosis | 11.2 | 2.7 | 9.7 | 1.6 | 7.8 | 2.1 | 5.7 | 9.7 | | 2.7 | 6.4 | |
| Enteropathic arthropathy | 0 | 0 | 0 | 0.1 | 0 | 0 | 0 | 0 | | 0 | 0 | |
| Enthesopathies and synovial disorders | 19.9 | 16.9 | 46.7 | 41.9 | 14.5 | 14.4 | 47.9 | 16.5 | | 12 | 37.1 | |
| Epilepsy | 1.6 | 3 | 3.9 | 10.4 | 1 | 1.7 | 4.2 | 0.8 | | 1.9 | 6.2 | |
| Erectile dysfunction | 1.2 | 2.8 | 3 | 17 | 1.6 | 3.2 | 12.9 | 1.4 | | 2.8 | 8.7 | |
| Female genital prolapse | 3.3 | 1.5 | 10.9 | 6.6 | 1.5 | 1.2 | 7.7 | 1.4 | | 1.3 | 6.1 | |
| Female infertility | 99.9 | 0 | 4.9 | 4.1 | 99.9 | 0 | 9.5 | 99.9 | | 0 | 8.1 | |
| Fibromyalgia | 0.8 | 0.5 | 8.8 | 2.8 | 1.1 | 0.5 | 7.5 | 0.2 | | 0.3 | 4.8 | |
| Folate deficiency, with and without anaemia | 0.5 | 0.4 | 1.7 | 2.9 | 1.4 | 1.3 | 3.4 | 0.7 | | 0.8 | 3.1 | |
| Fracture of hip | 0.7 | 1.3 | 0.8 | 6.1 | 0.3 | 0.4 | 1 | 0.3 | | 0.5 | 0.9 | |
| Gastritis and duodenitis | 6 | 6.5 | 23.9 | 29.8 | 6.3 | 8.4 | 33.7 | 6.7 | | 7.7 | 25.9 | |
| Gastrointestinal angiodysplasia | 0 | 0 | 0.2 | 0.6 | 0 | 0 | 0.3 | 0 | | 0 | 0.2 | |
| Gastro-oesophageal reflux disease | 9.7 | 9.2 | 36.5 | 33.3 | 11.5 | 12.2 | 42.2 | 10 | | 10 | 30.2 | |
| Giant Cell arteritis | 0 | 0 | 0.2 | 0.8 | 0 | 0 | 0.7 | 0 | | 0 | 0.2 | |
| Glaucoma | 0.6 | 0.4 | 1.5 | 5.6 | 0.5 | 0.4 | 3.7 | 1.1 | | 0.8 | 5.6 | |
| Glomerulonephritis and other nephritides | 0.3 | 0.4 | 0.8 | 5.7 | 0.2 | 0.4 | 4.7 | 0.2 | | 0.4 | 6.8 | |
| Gout | 0.8 | 1.4 | 2.3 | 11.6 | 0.4 | 1 | 5.9 | 0.6 | | 0.6 | 5.3 | |
| Hearing loss | 7.7 | 7.6 | 15.5 | 22.1 | 4.5 | 5 | 15.6 | 3 | | 3.4 | 9.9 | |
| Heart failure | 0.2 | 0.1 | 0.2 | 19.1 | 0.2 | 0.1 | 7.2 | 0.3 | | 0.2 | 9.5 | |
| Heart valve disease non-rheumatic | 0.7 | 0.6 | 1.1 | 14.3 | 0.4 | 0.4 | 6.3 | 0.5 | | 0.6 | 8.5 | |
| Hidradenitis suppurativa | 0.7 | 0.7 | 1.6 | 0.9 | 0.5 | 0.7 | 1.2 | 0.7 | | 1.1 | 2.1 | |
| HIV | 0.1 | 0.2 | 0.1 | 0.2 | 0 | 0.1 | 0.1 | 3 | | 3 | 1.3 | |
| Hodgkin Lymphoma | 0.1 | 0.2 | 0.1 | 0.6 | 0 | 0.1 | 0.3 | 0 | | 0.1 | 0.2 | |
| Hyperparathyroidism | 0.2 | 0.1 | 0.5 | 1.8 | 0.2 | 0.2 | 2.2 | 0.4 | | 0.2 | 2.9 | |
| Hyperplasia of prostate | 0.2 | 0.5 | 1.2 | 10 | 0.1 | 0.2 | 4.4 | 0.1 | | 0.3 | 3.1 | |
| Hyperprolactinaemia and prolactinoma | 0.8 | 0.2 | 0.6 | 0.4 | 1.2 | 0.4 | 0.8 | 1.9 | | 0.6 | 1.8 | |
| Hypertension | 9 | 7.3 | 28.2 | 70.3 | 7.1 | 7.5 | 54.6 | 18.6 | | 13.3 | 59.7 | |
| Hypertrophic Cardiomyopathy | 0 | 0 | 0 | 0.6 | 0 | 0.1 | 0.3 | 0 | | 0.1 | 0.9 | |
| Hypertrophy of nasal turbinates | 1.7 | 2.9 | 3.9 | 2.7 | 1.9 | 4.2 | 4.6 | 1 | | 1.4 | 2 | |
| Hypopituitarism | 0.2 | 0.1 | 0.3 | 0.8 | 0.4 | 0.2 | 0.7 | 0.2 | | 0.1 | 0.9 | |
| Hyposplenism | 0.1 | 0.2 | 0.2 | 1.5 | 0.1 | 0.1 | 0.4 | 0.1 | | 0.1 | 1.1 | |
| Immunodeficiencies | 0 | 0 | 0.1 | 0.5 | 0 | 0 | 0.2 | 0 | | 0 | 0.3 | |
| Infection of bones and joints | 0.2 | 0.4 | 0.7 | 4.4 | 0.3 | 0.4 | 1.9 | 0.3 | | 0.5 | 3 | |
| Intervertebral disc disorders | 3.7 | 3.3 | 15.7 | 14.5 | 1.6 | 2.5 | 13.2 | 1.6 | | 1.4 | 10.1 | |
| Intracerebral haemorrhage | 0.1 | 0.1 | 0.2 | 1.9 | 0 | 0.1 | 1.2 | 0.2 | | 0.2 | 1.2 | |
| Intracranial hypertension | 0.2 | 0.2 | 0.4 | 0.2 | 0.1 | 0.1 | 0.3 | 0.1 | | 0.3 | 1.1 | |
| Iron deficiency with and without anaemia | 5.9 | 4.1 | 12.7 | 17.9 | 17 | 15.8 | 37.7 | 16 | | 12.6 | 27.5 | |
| Irritable bowel syndrome | 12.8 | 10.6 | 32.3 | 14.1 | 7.4 | 7.1 | 19.2 | 5.6 | | 5.9 | 14.4 | |
| Juvenile arthritis | 0.1 | 0.1 | 0.2 | 0.2 | 0 | 0 | 0.1 | 0 | | 0 | 0.2 | |
| Learning disability | 0.3 | 1.2 | 1.1 | 3.1 | 0.2 | 0.7 | 1.4 | 0 | | 1.2 | 2.4 | |
| Leukaemia | 0.2 | 0.1 | 0.2 | 1.2 | 0 | 0.1 | 0.7 | 0 | | 0.1 | 0.5 | |
| Lichen planus | 0.5 | 0.4 | 1.3 | 1.6 | 1.3 | 1.2 | 4.1 | 0.5 | | 0.6 | 1.8 | |
| Liver failure and transplant | 0.1 | 0.1 | 0.1 | 3.8 | 0 | 0.1 | 1.2 | 0.1 | | 0.1 | 1.5 | |
| Liver fibrosis, sclerosis and cirrhosis | 0.1 | 0.2 | 0.3 | 6.3 | 0.1 | 0.2 | 2.3 | 0.1 | | 0.2 | 2.3 | |
| Lupus erythematosus (local and systemic) | 0.2 | 0.2 | 0.8 | 1 | 0.3 | 0.4 | 1.9 | 0.4 | | 0.5 | 3.9 | |
| Macular degeneration | 0.2 | 0.1 | 0.5 | 4.1 | 0 | 0.1 | 1.5 | 0.2 | | 0.1 | 1.5 | |
| Male infertility | 87.6 | 0.2 | 3.2 | 4 | 88.1 | 0.2 | 8.1 | 85.7 | | 0.1 | 6 | |
| Meniere disease | 0.2 | 0.2 | 1 | 1.1 | 0 | 0.1 | 0.7 | 0.1 | | 0 | 0.5 | |
| Menorrhagia and polymenorrhoea | 21.2 | 11.5 | 40 | 10.9 | 19.8 | 13.3 | 31.3 | 29 | | 16.8 | 34.6 | |
| Migraine | 13.9 | 12.9 | 30.1 | 14.5 | 11.9 | 11.7 | 24.2 | 10 | | 11.6 | 19.3 | |
| Motor neuron disease | 0 | 0 | 0.1 | 0.2 | 0 | 0 | 0.2 | 0 | | 0 | 0.1 | |
| Multiple myeloma and malignant plasma cell neoplasms | 0 | 0 | 0.1 | 0.6 | 0 | 0 | 0.2 | 0 | | 0 | 0.7 | |
| Multiple sclerosis | 0.5 | 0.4 | 1.3 | 1.2 | 0.3 | 0.3 | 0.5 | 0.1 | | 0.2 | 1.3 | |
| Myasthenia gravis | 0 | 0 | 0.1 | 0.2 | 0 | 0 | 0.2 | 0.1 | | 0.1 | 0.3 | |
| Myelodysplastic syndromes | 0 | 0 | 0 | 0.5 | 0 | 0 | 0.4 | 0.1 | | 0.1 | 0.4 | |
| Nasal polyp | 1 | 1.3 | 2.7 | 3.2 | 1 | 2 | 3.1 | 0.8 | | 1.1 | 2.2 | |
| Neuromuscular dysfunction of bladder | 0.7 | 0.5 | 3.7 | 3.7 | 0.4 | 0.6 | 4.6 | 0.9 | | 0.5 | 4.2 | |
| Non-acute cystitis | 0.4 | 0.2 | 1.2 | 1.4 | 0.3 | 0.2 | 1 | 0.3 | | 0.1 | 1.1 | |
| Non-alcoholic fatty liver disease and steatohepatitis | 1 | 0.9 | 4.3 | 8.6 | 1.5 | 1.9 | 9.7 | 1.2 | | 0.8 | 5.8 | |
| Non-diabetic peripheral neuropathies (excluding cranial nerves and carpal tunnel syndrome) | 1.7 | 1.5 | 6 | 12.9 | 0.8 | 1.1 | 8.1 | 1.1 | | 1 | 8.1 | |
| Non-Hodgkin Lymphoma | 0.2 | 0.2 | 0.3 | 1.6 | 0.1 | 0.1 | 0.7 | 0.1 | | 0.2 | 0.7 | |
| Non-malignant tumour of brain, central nervous system and pituitary | 0.4 | 0.3 | 0.8 | 1.2 | 0.3 | 0.3 | 1.2 | 0.7 | | 0.4 | 1.8 | |
| Obesity | 12.8 | 12 | 29.5 | 32.3 | 14.4 | 12.4 | 31.2 | 22.4 | | 18.7 | 37.9 | |
| Obsessive-compulsive disorder | 0.9 | 1.4 | 2.9 | 2 | 0.5 | 0.7 | 1.2 | 0.3 | | 0.4 | 0.7 | |
| Obstructive and reflux uropathy | 0.9 | 1.1 | 1.7 | 4.9 | 0.8 | 1.4 | 3.3 | 1 | | 0.8 | 3.2 | |
| Oesophagitis and oesophageal ulcer | 4 | 4.3 | 21.4 | 23.8 | 4.2 | 4.7 | 23.8 | 3.6 | | 3.4 | 15.6 | |
| Osteoarthritis | 6.5 | 4.2 | 29.9 | 41.4 | 2.6 | 2.5 | 31.4 | 4.3 | | 3.2 | 28.2 | |
| Osteoporosis | 1.2 | 0.4 | 4.4 | 13.9 | 0.7 | 0.4 | 7.3 | 0.7 | | 0.2 | 4.2 | |
| Other anaemias | 7.6 | 5.4 | 14.9 | 26 | 19.4 | 17.6 | 41.3 | 21.4 | | 17.2 | 37.3 | |
| Other haemolytic anaemias | 0.1 | 0.1 | 0.1 | 0.5 | 0.6 | 0.6 | 0.9 | 0.7 | | 0.9 | 2.5 | |
| Other interstitial pulmonary diseases with fibrosis | 0 | 0 | 0.2 | 2.3 | 0 | 0.1 | 1.4 | 0.1 | | 0.1 | 1.7 | |
| Other psychoactive substance misuse | 2.3 | 6.7 | 6.4 | 11.3 | 0.5 | 2.4 | 3.4 | 1.2 | | 4.4 | 5.7 | |
| Painful conditions | 10 | 9 | 50.1 | 56.8 | 11.6 | 11.9 | 62.3 | 13.6 | | 10.8 | 51.5 | |
| Pancreatitis | 0.6 | 0.8 | 1.7 | 5.5 | 0.4 | 0.7 | 3.1 | 0.3 | | 0.7 | 3 | |
| Parkinson's disease | 0.1 | 0 | 0.2 | 1.7 | 0 | 0 | 0.6 | 0 | | 0 | 0.4 | |
| Peptic ulcer disease | 1 | 1.4 | 7 | 17.1 | 0.8 | 1.4 | 11.3 | 1.5 | | 1.9 | 8.3 | |
| Peripheral arterial disease | 0.2 | 0.2 | 0.7 | 11.6 | 0.1 | 0.1 | 3.5 | 0.1 | | 0.1 | 3.6 | |
| Peripheral venous and lymphatic disease | 6.7 | 5.7 | 12.6 | 21.7 | 3.9 | 4.5 | 14.6 | 2.3 | | 2.9 | 10.2 | |
| Personality disorder | 0.7 | 2.2 | 4.7 | 6.6 | 0.3 | 0.6 | 1.6 | 0.2 | | 1.3 | 3.3 | |
| Polycystic ovarian syndrome | 7.8 | 2.1 | 2.7 | 0.6 | 13.1 | 3.8 | 2.8 | 7.3 | | 2.5 | 2.3 | |
| Polycythaemia vera | 0 | 0.1 | 0.1 | 0.9 | 0.1 | 0 | 0.3 | 0 | | 0 | 0.2 | |
| Polymyalgia Rheumatica | 0.1 | 0 | 1 | 2.7 | 0 | 0 | 1.3 | 0.1 | | 0 | 0.7 | |
| Portal hypertension and oesophageal varices | 0.1 | 0.1 | 0.1 | 4.4 | 0 | 0.1 | 1.4 | 0.1 | | 0.1 | 1 | |
| Post-traumatic stress and stress-related disorders | 19.5 | 16.9 | 40.9 | 21.4 | 12.4 | 13.5 | 27.1 | 17.8 | | 15.8 | 30.7 | |
| Primary malignancy biliary tract | 0 | 0 | 0 | 0.4 | 0 | 0 | 0.1 | 0 | | 0 | 0.1 | |
| Primary malignancy bladder | 0.2 | 0.1 | 0.2 | 3.2 | 0 | 0.1 | 1 | 0.1 | | 0 | 1 | |
| Primary malignancy bone and articular cartilage | 0.1 | 0.1 | 0.1 | 0.4 | 0.1 | 0.1 | 0.3 | 0 | | 0.1 | 0.4 | |
| Primary malignancy brain, other CNS and intracranial | 0.1 | 0.1 | 0.2 | 0.9 | 0 | 0.1 | 0.3 | 0 | | 0.1 | 0.5 | |
| Primary malignancy breast | 2.4 | 0.9 | 4.1 | 4.1 | 1.1 | 0.5 | 2.8 | 1.2 | | 0.8 | 3.6 | |
| Primary malignancy cervical | 1.4 | 1.4 | 1.8 | 1 | 0.2 | 0.2 | 0.4 | 0.3 | | 0.7 | 0.7 | |
| Primary malignancy colorectal and anus | 0.3 | 0.2 | 0.6 | 3.5 | 0 | 0.1 | 1.1 | 0 | | 0.1 | 1.3 | |
| Primary malignancy kidney and ureter | 0.2 | 0.1 | 0.2 | 3.2 | 0 | 0.1 | 0.9 | 0.1 | | 0 | 1.2 | |
| Primary malignancy liver | 0 | 0 | 0 | 0.5 | 0 | 0 | 0.2 | 0 | | 0 | 0.2 | |
| Primary malignancy lung and trachea | 0.1 | 0.1 | 0.4 | 3.5 | 0 | 0 | 0.6 | 0 | | 0.1 | 1.1 | |
| Primary malignancy malignant melanoma | 0.8 | 0.5 | 0.9 | 1.7 | 0.2 | 0 | 0.1 | 0.1 | | 0 | 0.2 | |
| Primary malignancy mesothelioma | 0 | 0 | 0 | 0.1 | NA | NA | NA | NA | | NA | NA | |
| Primary malignancy oesophageal | 0 | 0 | 0 | 1 | 0 | 0 | 0.1 | 0.1 | | 0 | 0.2 | |
| Primary malignancy oro-pharyngeal | 0.1 | 0.1 | 0.2 | 1.3 | 0 | 0.1 | 0.4 | 0 | | 0.1 | 0.5 | |
| Primary malignancy other skin and subcutaneous tissue | 1.8 | 1 | 2.7 | 8.1 | 0.1 | 0.1 | 0.4 | 0 | | 0 | 0.5 | |
| Primary malignancy others | 1.7 | 1 | 3.2 | 15.4 | 0.6 | 0.5 | 4.5 | 0.5 | | 0.5 | 6.2 | |
| Primary malignancy ovarian | 0.3 | 0.1 | 0.4 | 0.8 | 0.2 | 0.1 | 0.5 | 0.1 | | 0 | 0.4 | |
| Primary malignancy pancreatic | 0 | 0 | 0.1 | 0.8 | 0 | 0 | 0.2 | 0 | | 0 | 0.6 | |
| Primary malignancy prostate | 0 | 0.1 | 0.1 | 3 | 0 | 0 | 0.6 | 0.3 | | 0.1 | 1.1 | |
| Primary malignancy stomach | 0 | 0 | 0.1 | 1.2 | 0 | 0 | 0.4 | 0.2 | | 0 | 0.4 | |
| Primary malignancy testicular | 0.1 | 0.2 | 0 | 0.5 | 0 | 0.1 | 0.1 | 0 | | 0 | 0.1 | |
| Primary malignancy thyroid | 0.8 | 0.5 | 1.4 | 6.8 | 0.3 | 0.4 | 1.7 | 0.2 | | 0.2 | 2.2 | |
| Primary malignancy uterine | 0.2 | 0 | 0.4 | 0.7 | 0.2 | 0 | 0.8 | 0.1 | | 0 | 0.6 | |
| Psoriasis | 4.8 | 5 | 7.7 | 9.1 | 2.5 | 3.4 | 6.7 | 0.8 | | 1.2 | 2.3 | |
| Psoriatic arthropathy | 0.3 | 0.3 | 1.3 | 1.4 | 0.2 | 0.3 | 1.3 | 0 | | 0 | 0.4 | |
| Ptosis of eyelid | 0.3 | 0.3 | 0.8 | 1.4 | 0.1 | 0.3 | 1.1 | 0.1 | | 0.3 | 1.5 | |
| Pulmonary hypertension | 0 | 0 | 0 | 2.6 | 0 | 0 | 1.3 | 0.1 | | 0.1 | 3.6 | |
| Respiratory failure | 0.2 | 0.3 | 0.7 | 9.7 | 0.1 | 0.2 | 3.4 | 0.1 | | 0.3 | 5.4 | |
| Retinal detachments and breaks | 0.4 | 0.5 | 0.6 | 3.1 | 0.2 | 0.4 | 1.6 | 0.3 | | 0.4 | 2.6 | |
| Retinal vascular occlusions | 0.1 | 0.1 | 0.2 | 1.4 | 0.1 | 0.1 | 0.8 | 0 | | 0 | 0.9 | |
| Rheumatic valve disease | 0 | 0 | 0 | 0.3 | 0 | 0 | 0.4 | 0 | | 0 | 0.6 | |
| Rheumatoid Arthritis | 1 | 0.6 | 4.3 | 6.5 | 1.3 | 1 | 6.8 | 0.8 | | 0.7 | 5.8 | |
| Rosacea | 4.2 | 3.3 | 6.7 | 4.3 | 2.6 | 2.5 | 3.7 | 0.9 | | 1.2 | 1.8 | |
| Sarcoidosis | 0.2 | 0.2 | 0.5 | 1.1 | 0.1 | 0.3 | 1.4 | 0.5 | | 0.5 | 2.4 | |
| SARS-CoV-2 | 0.8 | 0.8 | 0.9 | 1.2 | 1.3 | 1.4 | 2.3 | 1.1 | | 0.8 | 1.8 | |
| Schizophrenia and non-organic psychosis | 0.5 | 2.4 | 2.5 | 6.5 | 0.8 | 2.3 | 4.5 | 1.2 | | 5.8 | 8.6 | |
| Scoliosis | 0.9 | 1 | 2.1 | 3.3 | 0.5 | 0.4 | 1.9 | 0.3 | | 0.7 | 2.3 | |
| Seborrheic dermatitis | 5.1 | 4.9 | 9.3 | 8 | 5.8 | 7.2 | 11.7 | 3.8 | | 4.1 | 7.2 | |
| Secondary malignancy and metastasis | 0.8 | 0.3 | 1.2 | 8.3 | 0.2 | 0.2 | 2.4 | 0.3 | | 0.3 | 4.3 | |
| Secondary polycythaemia | 0.1 | 0.1 | 0.1 | 1.1 | 0.1 | 0.1 | 0.5 | 0 | | 0 | 0.3 | |
| Sick sinus syndrome | 0 | 0 | 0.1 | 0.7 | 0 | 0 | 0.1 | 0 | | 0 | 0.3 | |
| Sickle-cell anaemia | 0 | 0 | 0 | 0 | 0.2 | 0.1 | 0.3 | 2.1 | | 2.2 | 5.7 | |
| Sjogren's disease | 0.1 | 0 | 0.6 | 0.6 | 0.1 | 0.1 | 1.4 | 0.1 | | 0.1 | 1.3 | |
| Sleep apnoea | 0.8 | 1.1 | 3.9 | 8.2 | 0.7 | 1.2 | 6.2 | 1.1 | | 1.6 | 7.4 | |
| Somatoform and dissociative disorders | 4.6 | 4 | 14.8 | 7.4 | 5.4 | 5.1 | 15.9 | 5.3 | | 4.7 | 11.7 | |
| Spina bifida | 0.2 | 0.2 | 0.6 | 0.7 | 0 | 0.1 | 0.3 | 0.1 | | 0.1 | 0.4 | |
| Spinal stenosis | 0.3 | 0.2 | 3.2 | 4.8 | 0.3 | 0.3 | 4.1 | 0.1 | | 0.2 | 3.6 | |
| Spondylolisthesis | 0.3 | 0.2 | 1.6 | 2.1 | 0.1 | 0.1 | 1.3 | 0.1 | | 0.1 | 1 | |
| Spondylosis | 1.9 | 1 | 14 | 18.5 | 1.1 | 1.2 | 15.1 | 0.9 | | 0.7 | 11.1 | |
| Subarachnoid haemorrhage | 0.2 | 0.3 | 0.4 | 1.9 | 0.1 | 0.2 | 0.8 | 0.2 | | 0.2 | 0.9 | |
| Subdural haematoma | 0.1 | 0.2 | 0.1 | 1.7 | 0 | 0.1 | 0.7 | 0.1 | | 0.2 | 0.9 | |
| Supraventricular tachycardia | 0.7 | 0.8 | 1.5 | 4.5 | 0.3 | 0.6 | 2.2 | 0.4 | | 0.4 | 2.5 | |
| Systemic sclerosis | 0.1 | 0 | 0.1 | 0.3 | 0 | 0 | 0.4 | 0 | | 0 | 0.5 | |
| Thalassaemia | 0.1 | 0.1 | 0.1 | 0.1 | 1.4 | 1.5 | 2.4 | 1.2 | | 1.1 | 2.4 | |
| Thrombocytopenia primary, secondary and other | 0.7 | 0.7 | 0.7 | 4.7 | 0.6 | 0.8 | 2.6 | 0.9 | | 1.6 | 3.8 | |
| Thrombophilia | 1.4 | 0.7 | 1.2 | 1 | 1 | 0.4 | 1 | 0.8 | | 0.4 | 1.6 | |
| Thyroid disease | 8 | 5 | 14.8 | 16 | 12.5 | 9.7 | 17.8 | 5.3 | | 4.1 | 12.3 | |
| Tinnitus | 3.1 | 2.6 | 7.5 | 7.5 | 2.4 | 2.4 | 8.5 | 2.9 | | 1.8 | 6.4 | |
| Trigeminal neuralgia | 0.5 | 0.3 | 2.3 | 1.6 | 0.3 | 0.3 | 1.6 | 0.2 | | 0.2 | 1.4 | |
| Tuberculosis | 0.2 | 0.2 | 0.6 | 3.3 | 3.5 | 3.6 | 9.6 | 3.1 | | 3.5 | 3.8 | |
| Type 1 Diabetes | 0.3 | 0.8 | 0.2 | 1.4 | 0.1 | 0.3 | 0.1 | 0.3 | | 0.4 | 0.6 | |
| Type 2 Diabetes | 2.1 | 1.6 | 8.3 | 25.5 | 6.3 | 5.6 | 36.3 | 5.6 | | 3.9 | 24.7 | |
| Ulcerative colitis | 1 | 1.3 | 1.9 | 2.5 | 0.7 | 1.5 | 2.7 | 0.2 | | 0.6 | 1.3 | |
| Unspecified or Rare Diabetes | 0.4 | 0.4 | 0.7 | 7.3 | 1 | 0.8 | 6.2 | 0.9 | | 1 | 6 | |
| Urinary Incontinence | 4.8 | 2.8 | 17.2 | 15.8 | 3.6 | 3.7 | 18.1 | 3.1 | | 3 | 16 | |
| Urolithiasis | 2.6 | 3.3 | 6.7 | 11 | 2.6 | 4 | 10.1 | 1.7 | | 2.1 | 6.5 | |
| Urticaria | 6.2 | 5.6 | 11.7 | 6.8 | 7.6 | 7.8 | 15.6 | 5.2 | | 5.3 | 9.4 | |
| Venous thromboembolism | 1.6 | 1.9 | 4.6 | 15.9 | 0.7 | 1.1 | 6.2 | 1.3 | | 1.6 | 10.9 | |
| Visual impairment and blindness | 0.9 | 1.2 | 2.6 | 7.3 | 0.9 | 1.2 | 4.9 | 0.6 | | 1.4 | 7 | |
| Vitamin B12 deficiency with and without anaemia | 2.3 | 1.9 | 6.7 | 8.5 | 5.9 | 5.7 | 17.4 | 1.4 | | 1 | 4.5 | |
| Vitiligo | 0.4 | 0.4 | 0.7 | 0.5 | 1 | 1.2 | 2.2 | 0.9 | | 0.6 | 1.5 | |
