# Supplementary material for: Ethnic differences in early onset multimorbidity and associations with health service use, long-term prescribing, years of life lost, and mortality: A cross-sectional study using clustering in the UK Clinical Practice Research Datalink
Source: PLoS Med. 2023 Oct 27;20(10):e1004300. doi: 10.1371/journal.pmed.1004300 (PMC10610074; doi:10.1371/journal.pmed.1004300)
Supplement: S2 Table — (DOCX) [file pmed.1004300.s006.docx]

| **S2 Table. Prevalence of the 204 long-term conditions per 100 according to ethnic groups (White n = 777,906; South Asian n = 33,915; and Black or Black British n = 26,048). Population with early onset of multimorbidity (16 to 39 years old).** | | | | | | |
| --- | --- | --- | --- | --- | --- | --- |
| **Long-term condition** | **White** | | **South Asian** | | **Black African/Caribbean** | |
|  | **Count** | **Prevalence** | **Count** | **Prevalence** | **Count** | **Prevalence** |
| ADHD and hyperkinetic disorders | 4192 | 0.5 | 37 | 0.1 | 50 | 0.2 |
| Adrenal insufficiency and Addison's disease | 1631 | 0.2 | 57 | 0.2 | 47 | 0.2 |
| Alcohol dependence and related disease | 62518 | 8.0 | 1073 | 3.2 | 1020 | 3.9 |
| Allergic and chronic rhinitis | 166989 | 21.5 | 9384 | 27.7 | 7506 | 28.8 |
| Alopecia areata and scarring alopecia | 4962 | 0.6 | 555 | 1.6 | 233 | 0.9 |
| Ankylosing spondylitis | 3492 | 0.4 | 92 | 0.3 | 22 | 0.1 |
| Anxiety and phobia | 265640 | 34.1 | 5725 | 16.9 | 4194 | 16.1 |
| Aortic aneurysm | 2654 | 0.3 | 40 | 0.1 | 24 | 0.1 |
| Aplastic anaemias | 1742 | 0.2 | 78 | 0.2 | 84 | 0.3 |
| Asbestosis | 570 | 0.1 | 2 | 0.0 | 1 | 0.0 |
| Asthma | 194209 | 25.0 | 6718 | 19.8 | 4794 | 18.4 |
| Atrial fibrillation and flutter | 23830 | 3.1 | 329 | 1.0 | 314 | 1.2 |
| Autism and Asperger's syndrome | 3599 | 0.5 | 55 | 0.2 | 96 | 0.4 |
| Autoimmune liver disease | 985 | 0.1 | 50 | 0.1 | 29 | 0.1 |
| Barrett's oesophagus | 6409 | 0.8 | 88 | 0.3 | 30 | 0.1 |
| Bipolar affective disorder and mania | 15626 | 2.0 | 409 | 1.2 | 547 | 2.1 |
| Blistering autoimmune skin conditions | 867 | 0.1 | 40 | 0.1 | 22 | 0.1 |
| Bronchiectasis | 6093 | 0.8 | 190 | 0.6 | 116 | 0.4 |
| Cardiac conduction defects | 11377 | 1.5 | 303 | 0.9 | 205 | 0.8 |
| Cardiomyopathy other | 4038 | 0.5 | 121 | 0.4 | 184 | 0.7 |
| Cerebral palsy | 1623 | 0.2 | 33 | 0.1 | 42 | 0.2 |
| Cerebrovascular disease | 19273 | 2.5 | 488 | 1.4 | 401 | 1.5 |
| Cervical carcinoma in situ | 35038 | 4.5 | 358 | 1.1 | 957 | 3.7 |
| Cholelithiasis | 57340 | 7.4 | 1757 | 5.2 | 1231 | 4.7 |
| Chronic fatigue | 15450 | 2.0 | 229 | 0.7 | 135 | 0.5 |
| Chronic kidney disease | 28909 | 3.7 | 992 | 2.9 | 1119 | 4.3 |
| Chronic obstructive pulmonary disease | 32685 | 4.2 | 406 | 1.2 | 287 | 1.1 |
| Chronic sinusitis | 96459 | 12.4 | 2877 | 8.5 | 1599 | 6.1 |
| Chronic ulcer of the skin | 37983 | 4.9 | 921 | 2.7 | 630 | 2.4 |
| Chronic viral hepatitis | 8042 | 1.0 | 402 | 1.2 | 738 | 2.8 |
| Coeliac disease | 6331 | 0.8 | 290 | 0.9 | 37 | 0.1 |
| Collapsed vertebra | 2624 | 0.3 | 44 | 0.1 | 26 | 0.1 |
| Congenital cardiac disease | 7905 | 1.0 | 256 | 0.8 | 165 | 0.6 |
| Constipation | 140503 | 18.1 | 7834 | 23.1 | 5392 | 20.7 |
| Coronary heart disease | 45160 | 5.8 | 1808 | 5.3 | 714 | 2.7 |
| Crohn's disease | 9931 | 1.3 | 360 | 1.1 | 127 | 0.5 |
| Cystic fibrosis | 615 | 0.1 | 14 | 0.0 | 13 | 0.0 |
| Cystic renal disease | 1471 | 0.2 | 43 | 0.1 | 45 | 0.2 |
| Dementia | 7002 | 0.9 | 108 | 0.3 | 79 | 0.3 |
| Depression | 379238 | 48.8 | 8729 | 25.7 | 7466 | 28.7 |
| Dermatitis (atopic/contact/other/unspecified) | 232811 | 29.9 | 11477 | 33.8 | 6563 | 25.2 |
| Diabetic eye disease | 16503 | 2.1 | 1116 | 3.3 | 601 | 2.3 |
| Diabetic neurological complications | 4942 | 0.6 | 181 | 0.5 | 110 | 0.4 |
| Disorders of autonomic nervous system | 5642 | 0.7 | 157 | 0.5 | 87 | 0.3 |
| Diverticular disease of intestine | 44018 | 5.7 | 515 | 1.5 | 500 | 1.9 |
| Down's syndrome | 710 | 0.1 | 27 | 0.1 | 15 | 0.1 |
| Dysmenorrhoea | 61487 | 7.9 | 2642 | 7.8 | 2554 | 9.8 |
| Eating disorders | 19606 | 2.5 | 429 | 1.3 | 338 | 1.3 |
| End stage renal disease | 3828 | 0.5 | 232 | 0.7 | 258 | 1.0 |
| Endometriosis | 35014 | 4.5 | 1168 | 3.4 | 1048 | 4.0 |
| Enteropathic arthropathy | 163 | 0.0 | 3 | 0.0 | 2 | 0.0 |
| Enthesopathies and synovial disorders | 198472 | 25.5 | 6936 | 20.5 | 4288 | 16.5 |
| Epilepsy | 30703 | 3.9 | 710 | 2.1 | 640 | 2.5 |
| Erectile dysfunction | 33454 | 4.3 | 1602 | 4.7 | 939 | 3.6 |
| Female genital prolapse | 30768 | 4.0 | 818 | 2.4 | 526 | 2.0 |
| Female infertility | 60400 | 7.8 | 4516 | 13.3 | 3061 | 11.8 |
| Fibromyalgia | 18120 | 2.3 | 618 | 1.8 | 265 | 1.0 |
| Folate deficiency, with and without anaemia | 7073 | 0.9 | 574 | 1.7 | 299 | 1.1 |
| Fracture of hip | 13543 | 1.7 | 158 | 0.5 | 138 | 0.5 |
| Gastritis and duodenitis | 95997 | 12.3 | 4297 | 12.7 | 2714 | 10.4 |
| Gastrointestinal angiodysplasia | 857 | 0.1 | 21 | 0.1 | 10 | 0.0 |
| Gastro-oesophageal reflux disease | 132758 | 17.1 | 5943 | 17.5 | 3428 | 13.2 |
| Giant cell arteritis | 1080 | 0.1 | 43 | 0.1 | 10 | 0.0 |
| Glaucoma | 9071 | 1.2 | 346 | 1.0 | 403 | 1.5 |
| Glomerulonephritis and other nephritides | 8151 | 1.0 | 397 | 1.2 | 362 | 1.4 |
| Gout | 20843 | 2.7 | 616 | 1.8 | 348 | 1.3 |
| Hearing loss | 83457 | 10.7 | 2330 | 6.9 | 1132 | 4.3 |
| Heart failure | 17366 | 2.2 | 462 | 1.4 | 421 | 1.6 |
| Heart valve disease non-rheumatic | 16991 | 2.2 | 486 | 1.4 | 465 | 1.8 |
| Hidradenitis suppurativa | 6717 | 0.9 | 256 | 0.8 | 323 | 1.2 |
| HIV | 1254 | 0.2 | 28 | 0.1 | 714 | 2.7 |
| Hodgkin lymphoma | 1423 | 0.2 | 42 | 0.1 | 29 | 0.1 |
| Hyperparathyroidism | 2762 | 0.4 | 180 | 0.5 | 158 | 0.6 |
| Hyperplasia of prostate | 12666 | 1.6 | 315 | 0.9 | 183 | 0.7 |
| Hyperprolactinaemia and prolactinoma | 2880 | 0.4 | 199 | 0.6 | 238 | 0.9 |
| Hypertension | 141866 | 18.2 | 5344 | 15.8 | 5498 | 21.1 |
| Hypertrophic cardiomyopathy | 717 | 0.1 | 37 | 0.1 | 51 | 0.2 |
| Hypertrophy of nasal turbinates | 23189 | 3.0 | 1370 | 4.0 | 380 | 1.5 |
| Hypopituitarism | 1830 | 0.2 | 95 | 0.3 | 66 | 0.3 |
| Hyposplenism | 2423 | 0.3 | 51 | 0.2 | 71 | 0.3 |
| Immunodeficiencies | 958 | 0.1 | 27 | 0.1 | 20 | 0.1 |
| Infection of bones and joints | 7040 | 0.9 | 220 | 0.6 | 232 | 0.9 |
| Intervertebral disc disorders | 53495 | 6.9 | 1466 | 4.3 | 719 | 2.8 |
| Intracerebral haemorrhage | 2560 | 0.3 | 92 | 0.3 | 88 | 0.3 |
| Intracranial hypertension | 1823 | 0.2 | 36 | 0.1 | 100 | 0.4 |
| Iron deficiency with and without anaemia | 57563 | 7.4 | 6763 | 19.9 | 4007 | 15.4 |
| Irritable bowel syndrome | 118879 | 15.3 | 3171 | 9.3 | 1887 | 7.2 |
| Juvenile arthritis | 828 | 0.1 | 20 | 0.1 | 17 | 0.1 |
| Learning disability | 10705 | 1.4 | 250 | 0.7 | 328 | 1.3 |
| Leukaemia | 2059 | 0.3 | 68 | 0.2 | 41 | 0.2 |
| Lichen planus | 5526 | 0.7 | 578 | 1.7 | 199 | 0.8 |
| Liver failure and transplant | 3921 | 0.5 | 98 | 0.3 | 84 | 0.3 |
| Liver fibrosis, sclerosis and cirrhosis | 6573 | 0.8 | 183 | 0.5 | 138 | 0.5 |
| Lupus erythematosus (local and systemic) | 2952 | 0.4 | 219 | 0.6 | 263 | 1.0 |
| Macular degeneration | 4764 | 0.6 | 114 | 0.3 | 86 | 0.3 |
| Male infertility | 52944 | 6.8 | 4030 | 11.9 | 2632 | 10.1 |
| Meniere disease | 3415 | 0.4 | 66 | 0.2 | 34 | 0.1 |
| Menorrhagia and polymenorrhoea | 135705 | 17.4 | 5877 | 17.3 | 5470 | 21.0 |
| Migraine | 128206 | 16.5 | 4764 | 14.0 | 3313 | 12.7 |
| Motor neuron disease | 482 | 0.1 | 16 | 0.0 | 5 | 0.0 |
| Multiple myeloma and malignant plasma cell neoplasms | 694 | 0.1 | 20 | 0.1 | 36 | 0.1 |
| Multiple sclerosis | 5317 | 0.7 | 119 | 0.4 | 97 | 0.4 |
| Myasthenia gravis | 458 | 0.1 | 15 | 0.0 | 29 | 0.1 |
| Myelodysplastic syndromes | 587 | 0.1 | 25 | 0.1 | 29 | 0.1 |
| Nasal polyp | 13682 | 1.8 | 704 | 2.1 | 333 | 1.3 |
| Neuromuscular dysfunction of bladder | 11544 | 1.5 | 427 | 1.3 | 300 | 1.2 |
| Non-acute cystitis | 4387 | 0.6 | 118 | 0.3 | 76 | 0.3 |
| Non-alcoholic fatty liver disease and steatohepatitis | 18774 | 2.4 | 1105 | 3.3 | 420 | 1.6 |
| Non-diabetic peripheral neuropathies (excluding cranial nerves and carpal tunnel syndrome) | 28106 | 3.6 | 783 | 2.3 | 542 | 2.1 |
| Non-Hodgkin lymphoma | 2830 | 0.4 | 82 | 0.2 | 71 | 0.3 |
| Non-malignant tumour of brain, central nervous system and pituitary | 3816 | 0.5 | 156 | 0.5 | 164 | 0.6 |
| Obesity | 137033 | 17.6 | 5435 | 16.0 | 5783 | 22.2 |
| Obsessive-compulsive disorder | 13229 | 1.7 | 254 | 0.7 | 115 | 0.4 |
| Obstructive and reflux uropathy | 12634 | 1.6 | 576 | 1.7 | 304 | 1.2 |
| Oesophagitis and oesophageal ulcer | 75035 | 9.6 | 2710 | 8.0 | 1381 | 5.3 |
| Osteoarthritis | 103125 | 13.3 | 2585 | 7.6 | 1871 | 7.2 |
| Osteoporosis | 20988 | 2.7 | 551 | 1.6 | 231 | 0.9 |
| Other anaemias | 74490 | 9.6 | 7517 | 22.2 | 5441 | 20.9 |
| Other haemolytic anaemias | 1421 | 0.2 | 229 | 0.7 | 295 | 1.1 |
| Other interstitial pulmonary diseases with fibrosis | 2433 | 0.3 | 102 | 0.3 | 81 | 0.3 |
| Other psychoactive substance misuse | 54107 | 7.0 | 797 | 2.3 | 1125 | 4.3 |
| Painful conditions | 171282 | 22.0 | 7057 | 20.8 | 4538 | 17.4 |
| Pancreatitis | 11291 | 1.5 | 384 | 1.1 | 256 | 1.0 |
| Parkinson's disease | 1966 | 0.3 | 42 | 0.1 | 16 | 0.1 |
| Peptic ulcer disease | 32208 | 4.1 | 1051 | 3.1 | 749 | 2.9 |
| Peripheral arterial disease | 12016 | 1.5 | 247 | 0.7 | 167 | 0.6 |
| Peripheral venous and lymphatic disease | 68961 | 8.9 | 2123 | 6.3 | 1047 | 4.0 |
| Personality disorder | 23619 | 3.0 | 252 | 0.7 | 382 | 1.5 |
| Polycystic ovarian syndrome | 19069 | 2.5 | 1610 | 4.7 | 779 | 3.0 |
| Polycythaemia vera | 1188 | 0.2 | 34 | 0.1 | 15 | 0.1 |
| Polymyalgia rheumatica | 3943 | 0.5 | 84 | 0.2 | 34 | 0.1 |
| Portal hypertension and oesophageal varices | 4369 | 0.6 | 100 | 0.3 | 60 | 0.2 |
| Post-traumatic stress and stress-related disorders | 172831 | 22.2 | 5395 | 15.9 | 4810 | 18.5 |
| Primary malignancy biliary tract | 391 | 0.1 | 7 | 0.0 | 6 | 0.0 |
| Primary malignancy bladder | 3649 | 0.5 | 73 | 0.2 | 52 | 0.2 |
| Primary malignancy bone and articular cartilage | 939 | 0.1 | 36 | 0.1 | 30 | 0.1 |
| Primary malignancy brain, other CNS and intracranial | 1786 | 0.2 | 52 | 0.2 | 33 | 0.1 |
| Primary malignancy breast | 15231 | 2.0 | 328 | 1.0 | 331 | 1.3 |
| Primary malignancy cervical | 11509 | 1.5 | 69 | 0.2 | 169 | 0.6 |
| Primary malignancy colorectal and anus | 4883 | 0.6 | 82 | 0.2 | 69 | 0.3 |
| Primary malignancy kidney and ureter | 3667 | 0.5 | 67 | 0.2 | 57 | 0.2 |
| Primary malignancy liver | 519 | 0.1 | 12 | 0.0 | 16 | 0.1 |
| Primary malignancy lung and trachea | 3953 | 0.5 | 49 | 0.1 | 59 | 0.2 |
| Primary malignancy malignant melanoma | 5878 | 0.8 | 25 | 0.1 | 15 | 0.1 |
| Primary malignancy mesothelioma | 155 | 0.0 | 0 | 0.0 | 0 | 0.0 |
| Primary malignancy oesophageal | 1004 | 0.1 | 10 | 0.0 | 12 | 0.0 |
| Primary malignancy oro-pharyngeal | 1951 | 0.3 | 53 | 0.2 | 33 | 0.1 |
| Primary malignancy other skin and subcutaneous tissue | 16570 | 2.1 | 46 | 0.1 | 31 | 0.1 |
| Primary malignancy others | 23950 | 3.1 | 420 | 1.2 | 356 | 1.4 |
| Primary malignancy ovarian | 1728 | 0.2 | 55 | 0.2 | 30 | 0.1 |
| Primary malignancy pancreatic | 879 | 0.1 | 14 | 0.0 | 25 | 0.1 |
| Primary malignancy prostate | 3244 | 0.4 | 35 | 0.1 | 71 | 0.3 |
| Primary malignancy stomach | 1177 | 0.2 | 25 | 0.1 | 27 | 0.1 |
| Primary malignancy testicular | 1741 | 0.2 | 33 | 0.1 | 16 | 0.1 |
| Primary malignancy thyroid | 10795 | 1.4 | 198 | 0.6 | 124 | 0.5 |
| Primary malignancy uterine | 1426 | 0.2 | 61 | 0.2 | 30 | 0.1 |
| Psoriasis | 46855 | 6.0 | 1314 | 3.9 | 352 | 1.4 |
| Psoriatic arthropathy | 4858 | 0.6 | 145 | 0.4 | 21 | 0.1 |
| Ptosis of eyelid | 3896 | 0.5 | 136 | 0.4 | 121 | 0.5 |
| Pulmonary hypertension | 2349 | 0.3 | 84 | 0.2 | 165 | 0.6 |
| Respiratory failure | 10680 | 1.4 | 263 | 0.8 | 279 | 1.1 |
| Retinal detachments and breaks | 6028 | 0.8 | 196 | 0.6 | 189 | 0.7 |
| Retinal vascular occlusions | 1855 | 0.2 | 65 | 0.2 | 48 | 0.2 |
| Rheumatic valve disease | 319 | 0.0 | 27 | 0.1 | 28 | 0.1 |
| Rheumatoid arthritis | 15148 | 1.9 | 693 | 2.0 | 386 | 1.5 |
| Rosacea | 32097 | 4.1 | 934 | 2.8 | 334 | 1.3 |
| Sarcoidosis | 2670 | 0.3 | 162 | 0.5 | 206 | 0.8 |
| SARS-CoV-2 | 6606 | 0.8 | 520 | 1.5 | 252 | 1.0 |
| Schizophrenia and non-organic psychosis | 21319 | 2.7 | 853 | 2.5 | 1505 | 5.8 |
| Scoliosis | 11214 | 1.4 | 234 | 0.7 | 242 | 0.9 |
| Seborrheic dermatitis | 47623 | 6.1 | 2674 | 7.9 | 1186 | 4.6 |
| Secondary malignancy and metastasis | 10726 | 1.4 | 198 | 0.6 | 228 | 0.9 |
| Secondary polycythaemia | 1564 | 0.2 | 45 | 0.1 | 21 | 0.1 |
| Sick sinus syndrome | 829 | 0.1 | 15 | 0.0 | 20 | 0.1 |
| Sickle-cell anaemia | 162 | 0.0 | 56 | 0.2 | 718 | 2.8 |
| Sjogren's disease | 1601 | 0.2 | 110 | 0.3 | 66 | 0.3 |
| Sleep apnoea | 18558 | 2.4 | 691 | 2.0 | 640 | 2.5 |
| Somatoform and dissociative disorders | 50369 | 6.5 | 2398 | 7.1 | 1532 | 5.9 |
| Spina bifida | 2680 | 0.3 | 41 | 0.1 | 34 | 0.1 |
| Spinal stenosis | 9994 | 1.3 | 321 | 0.9 | 188 | 0.7 |
| Spondylolisthesis | 5313 | 0.7 | 103 | 0.3 | 69 | 0.3 |
| Spondylosis | 42135 | 5.4 | 1237 | 3.6 | 608 | 2.3 |
| Subarachnoid haemorrhage | 3957 | 0.5 | 91 | 0.3 | 90 | 0.3 |
| Subdural haematoma | 2869 | 0.4 | 68 | 0.2 | 71 | 0.3 |
| Supraventricular tachycardia | 10342 | 1.3 | 275 | 0.8 | 193 | 0.7 |
| Systemic sclerosis | 572 | 0.1 | 28 | 0.1 | 25 | 0.1 |
| Thalassaemia | 822 | 0.1 | 572 | 1.7 | 349 | 1.3 |
| Thrombocytopenia primary, secondary and other | 8749 | 1.1 | 371 | 1.1 | 494 | 1.9 |
| Thrombophilia | 6674 | 0.9 | 207 | 0.6 | 164 | 0.6 |
| Thyroid disease | 64220 | 8.3 | 3925 | 11.6 | 1438 | 5.5 |
| Tinnitus | 32166 | 4.1 | 1180 | 3.5 | 695 | 2.7 |
| Trigeminal neuralgia | 6675 | 0.9 | 172 | 0.5 | 111 | 0.4 |
| Tuberculosis | 4954 | 0.6 | 1593 | 4.7 | 916 | 3.5 |
| Type 1 diabetes | 5453 | 0.7 | 95 | 0.3 | 108 | 0.4 |
| Type 2 diabetes | 42951 | 5.5 | 3761 | 11.1 | 1896 | 7.3 |
| Ulcerative colitis | 11884 | 1.5 | 547 | 1.6 | 164 | 0.6 |
| Unspecified or rare diabetes | 9446 | 1.2 | 598 | 1.8 | 452 | 1.7 |
| Urinary incontinence | 55033 | 7.1 | 2115 | 6.2 | 1307 | 5.0 |
| Urolithiasis | 37184 | 4.8 | 1673 | 4.9 | 719 | 2.8 |
| Urticaria | 54029 | 6.9 | 3122 | 9.2 | 1550 | 6.0 |
| Venous thromboembolism | 30667 | 3.9 | 660 | 1.9 | 793 | 3.0 |
| Visual impairment and blindness | 16462 | 2.1 | 611 | 1.8 | 572 | 2.2 |
| Vitamin B12 deficiency with and without anaemia | 27777 | 3.6 | 2649 | 7.8 | 407 | 1.6 |
| Vitiligo | 3786 | 0.5 | 463 | 1.4 | 208 | 0.8 |
| ADHD: attention deficit hyperactivity disorder, CNS: central nervous system, HIV: human immunodeficiency virus, SARS-CoV-2: severe acute respiratory syndrome coronavirus 2. | | | | | | |

| **S1 Table**. Prevalence of the 204 long-term conditions per 100 according to clusters within each ethnic group (White n = 777,906; South Asian n = 33,915; and Black or Black British n = 26,048). Population with early onset of multimorbidity (16 to 39 years old). | | | | | | | | | | | | | |
| --- | --- | --- | --- | --- | --- | --- | --- | --- | --- | --- | --- | --- | --- |
| **Long-term condition** | **White** | | | | **South Asian** | | | | **Black African/Caribbean** | | |  |  |
|  | **Cluster 1** | **Cluster 2** | **Cluster 3** | **Cluster 4** | **Cluster 1** | **Cluster 2** | **Cluster 3** | **Cluster 1** | | **Cluster 2** | **Cluster 3** |  |  |
| ADHD and hyperkinetic disorders | 0.7 | 0.3 | 0.3 | 0.2 | 0.1 | 0.1 | 0 | 0.2 | | 0.3 | 0 |  |  |
| Adrenal insufficiency and Addison's disease | 0.1 | 0.3 | 0.9 | 0.1 | 0.1 | 0.5 | 0 | 0.1 | | 0.7 | 0.1 |  |  |
| Alcohol dependence and related disease | 7.2 | 6.2 | 18.2 | 3 | 3.1 | 4.4 | 1.2 | 3.7 | | 6.8 | 1.2 |  |  |
| Allergic and chronic rhinitis | 18.9 | 30.9 | 20.7 | 17.9 | 25.7 | 39.2 | 20.4 | 27.3 | | 37.8 | 24.1 |  |  |
| Alopecia areata and scarring alopecia | 0.6 | 0.8 | 0.8 | 0.5 | 1.6 | 2.1 | 1.1 | 0.8 | | 1.4 | 1 |  |  |
| Ankylosing spondylitis | 0.3 | 0.8 | 1.2 | 0.2 | 0.2 | 0.7 | 0.1 | 0.1 | | 0.2 | 0 |  |  |
| Anxiety and phobia | 28 | 53.2 | 41.4 | 23.9 | 14 | 32.1 | 10.5 | 14.1 | | 28.6 | 10.3 |  |  |
| Aortic aneurysm | 0 | 0.1 | 2.6 | 0 | 0 | 0.6 | 0 | 0 | | 0.4 | 0 |  |  |
| Aplastic anaemias | 0.1 | 0.1 | 1.5 | 0.1 | 0.1 | 0.9 | 0.1 | 0.2 | | 1.2 | 0.1 |  |  |
| Asbestosis | 0 | 0 | 0.6 | 0 | 0 | 0 | 0 | 0 | | 0 | 0 |  |  |
| Asthma | 22.1 | 32.1 | 31.5 | 18.1 | 17.7 | 32.4 | 12 | 16.7 | | 29.8 | 12.6 |  |  |
| Atrial fibrillation and flutter | 0.7 | 1.4 | 21.1 | 0.6 | 0.2 | 4.6 | 0.1 | 0.4 | | 5.7 | 0.1 |  |  |
| Autism and Asperger's syndrome | 0.5 | 0.3 | 0.5 | 0.1 | 0.2 | 0.2 | 0 | 0.4 | | 0.5 | 0 |  |  |
| Autoimmune liver disease | 0 | 0.1 | 0.7 | 0 | 0.1 | 0.6 | 0 | 0.1 | | 0.4 | 0 |  |  |
| Barrett's oesophagus | 0.2 | 1.7 | 3.2 | 0.2 | 0.1 | 0.9 | 0 | 0 | | 0.5 | 0 |  |  |
| Bipolar affective disorder and mania | 1.5 | 3 | 4.1 | 0.6 | 1 | 2.4 | 0.4 | 2 | | 3.7 | 0.5 |  |  |
| Blistering autoimmune skin conditions | 0.1 | 0.1 | 0.4 | 0 | 0.1 | 0.3 | 0 | 0.1 | | 0.2 | 0.1 |  |  |
| Bronchiectasis | 0.2 | 1 | 4.3 | 0.3 | 0.2 | 2 | 0.2 | 0.2 | | 1.7 | 0.1 |  |  |
| Cardiac conduction defects | 0.5 | 0.8 | 8.9 | 0.4 | 0.3 | 3.8 | 0.2 | 0.4 | | 3.4 | 0.1 |  |  |
| Cardiomyopathy other | 0.1 | 0.1 | 3.8 | 0.1 | 0.1 | 1.6 | 0.1 | 0.2 | | 3.5 | 0.1 |  |  |
| Cerebral Palsy | 0.2 | 0.2 | 0.5 | 0 | 0.1 | 0.1 | 0 | 0.1 | | 0.4 | 0 |  |  |
| Cerebrovascular disease | 0.6 | 2.3 | 14.7 | 0.7 | 0.4 | 6.5 | 0.2 | 0.5 | | 7.3 | 0.4 |  |  |
| Cervical carcinoma in situ | 4.4 | 5.8 | 1.9 | 5.3 | 1 | 1.4 | 0.9 | 3.7 | | 3.5 | 3.2 |  |  |
| Cholelithiasis | 4.1 | 14.8 | 14.2 | 5.7 | 3.6 | 12.4 | 3.9 | 3.5 | | 11 | 4 |  |  |
| Chronic fatigue | 1.1 | 5.3 | 2 | 1.1 | 0.3 | 2.3 | 0.3 | 0.3 | | 1.6 | 0.1 |  |  |
| Chronic Kidney Disease | 0.6 | 3.7 | 23.3 | 1.2 | 0.9 | 12.6 | 0.8 | 1.5 | | 18.9 | 2.6 |  |  |
| Chronic obstructive pulmonary disease | 0.9 | 5.7 | 22.8 | 0.9 | 0.3 | 5.3 | 0.2 | 0.4 | | 4.9 | 0.2 |  |  |
| Chronic sinusitis | 8.3 | 25.2 | 15.7 | 9.2 | 6.6 | 18.3 | 4.5 | 4.7 | | 14 | 4.6 |  |  |
| Chronic ulcer of the skin | 2.5 | 5 | 20.1 | 2.2 | 1.7 | 8 | 0.7 | 1.3 | | 9 | 0.8 |  |  |
| Chronic viral hepatitis | 0.8 | 0.6 | 3.1 | 0.5 | 1 | 2.2 | 0.9 | 2.7 | | 3.6 | 2.1 |  |  |
| Coeliac disease | 0.6 | 1.4 | 1.1 | 0.5 | 0.8 | 1.3 | 0.6 | 0.1 | | 0.2 | 0.1 |  |  |
| Collapsed vertebra | 0.1 | 0.3 | 2.1 | 0.1 | 0 | 0.6 | 0 | 0 | | 0.4 | 0.1 |  |  |
| Congenital cardiac disease | 0.8 | 0.7 | 3.2 | 0.6 | 0.6 | 1.6 | 0.5 | 0.4 | | 2 | 0.2 |  |  |
| Constipation | 10.5 | 33.6 | 37.3 | 13.5 | 18.4 | 43.2 | 19.6 | 17 | | 39 | 18.4 |  |  |
| Coronary heart disease | 1.2 | 5.5 | 36 | 1.2 | 1.5 | 23.6 | 1.1 | 0.7 | | 14.1 | 0.7 |  |  |
| Crohn's disease | 1 | 1.9 | 2 | 0.7 | 0.9 | 1.9 | 0.6 | 0.4 | | 1.1 | 0.2 |  |  |
| Cystic Fibrosis | 0 | 0.1 | 0.2 | 0.1 | 0 | 0.1 | 0 | 0 | | 0.2 | 0 |  |  |
| Cystic renal disease | 0.1 | 0.1 | 0.8 | 0.1 | 0.1 | 0.5 | 0 | 0.1 | | 0.6 | 0.1 |  |  |
| Dementia | 0.1 | 0.4 | 7.1 | 0.1 | 0 | 1.7 | 0 | 0.1 | | 1.6 | 0.1 |  |  |
| Depression | 40.9 | 72 | 58.8 | 35.8 | 21.1 | 48.9 | 17.1 | 25.5 | | 49.2 | 19.1 |  |  |
| Dermatitis (atopic/contact/other/unspecified) | 26.1 | 40 | 34.1 | 27.3 | 30.8 | 48.2 | 28.4 | 23.3 | | 35.4 | 21.3 |  |  |
| Diabetic eye disease | 0.7 | 1.2 | 12.9 | 0.6 | 1 | 14.2 | 0.8 | 0.6 | | 11.2 | 1.2 |  |  |
| Diabetic neurological complications | 0 | 0.1 | 5.5 | 0 | 0 | 3 | 0 | 0 | | 2.6 | 0.1 |  |  |
| Disorders of autonomic nervous system | 0.3 | 1.3 | 2.7 | 0.2 | 0.2 | 1.9 | 0.1 | 0.1 | | 1.7 | 0.1 |  |  |
| Diverticular disease of intestine | 1.7 | 11.5 | 20.5 | 2.6 | 0.6 | 5.9 | 0.5 | 0.7 | | 8.2 | 1.2 |  |  |
| Down's syndrome | 0.1 | 0.1 | 0.2 | 0.1 | 0 | 0.2 | 0.1 | 0 | | 0.2 | 0 |  |  |
| Dysmenorrhoea | 5.8 | 17 | 2.8 | 10.8 | 6.2 | 12.4 | 9.9 | 8.1 | | 14.2 | 14.9 |  |  |
| Eating disorders | 2 | 3.8 | 3.6 | 1.8 | 1.1 | 2.4 | 0.7 | 1.1 | | 2.7 | 0.7 |  |  |
| End stage renal disease | 0.1 | 0 | 4 | 0 | 0.1 | 3.7 | 0 | 0.1 | | 6.1 | 0.2 |  |  |
| Endometriosis | 2.7 | 9.7 | 1.6 | 11.2 | 2.1 | 5.7 | 7.8 | 2.7 | | 6.4 | 9.7 |  |  |
| Enteropathic arthropathy | 0 | 0 | 0.1 | 0 | 0 | 0 | 0 | 0 | | 0 | 0 |  |  |
| Enthesopathies and synovial disorders | 16.9 | 46.7 | 41.9 | 19.9 | 14.4 | 47.9 | 14.5 | 12 | | 37.1 | 16.5 |  |  |
| Epilepsy | 3 | 3.9 | 10.4 | 1.6 | 1.7 | 4.2 | 1 | 1.9 | | 6.2 | 0.8 |  |  |
| Erectile dysfunction | 2.8 | 3 | 17 | 1.2 | 3.2 | 12.9 | 1.6 | 2.8 | | 8.7 | 1.4 |  |  |
| Female genital prolapse | 1.5 | 10.9 | 6.6 | 3.3 | 1.2 | 7.7 | 1.5 | 1.3 | | 6.1 | 1.4 |  |  |
| Female infertility | 0 | 4.9 | 4.1 | 99.9 | 0 | 9.5 | 99.9 | 0 | | 8.1 | 99.9 |  |  |
| Fibromyalgia | 0.5 | 8.8 | 2.8 | 0.8 | 0.5 | 7.5 | 1.1 | 0.3 | | 4.8 | 0.2 |  |  |
| Folate deficiency, with and without anaemia | 0.4 | 1.7 | 2.9 | 0.5 | 1.3 | 3.4 | 1.4 | 0.8 | | 3.1 | 0.7 |  |  |
| Fracture of hip | 1.3 | 0.8 | 6.1 | 0.7 | 0.4 | 1 | 0.3 | 0.5 | | 0.9 | 0.3 |  |  |
| Gastritis and duodenitis | 6.5 | 23.9 | 29.8 | 6 | 8.4 | 33.7 | 6.3 | 7.7 | | 25.9 | 6.7 |  |  |
| Gastrointestinal angiodysplasia | 0 | 0.2 | 0.6 | 0 | 0 | 0.3 | 0 | 0 | | 0.2 | 0 |  |  |
| Gastro-oesophageal reflux disease | 9.2 | 36.5 | 33.3 | 9.7 | 12.2 | 42.2 | 11.5 | 10 | | 30.2 | 10 |  |  |
| Giant Cell arteritis | 0 | 0.2 | 0.8 | 0 | 0 | 0.7 | 0 | 0 | | 0.2 | 0 |  |  |
| Glaucoma | 0.4 | 1.5 | 5.6 | 0.6 | 0.4 | 3.7 | 0.5 | 0.8 | | 5.6 | 1.1 |  |  |
| Glomerulonephritis and other nephritides | 0.4 | 0.8 | 5.7 | 0.3 | 0.4 | 4.7 | 0.2 | 0.4 | | 6.8 | 0.2 |  |  |
| Gout | 1.4 | 2.3 | 11.6 | 0.8 | 1 | 5.9 | 0.4 | 0.6 | | 5.3 | 0.6 |  |  |
| Hearing loss | 7.6 | 15.5 | 22.1 | 7.7 | 5 | 15.6 | 4.5 | 3.4 | | 9.9 | 3 |  |  |
| Heart failure | 0.1 | 0.2 | 19.1 | 0.2 | 0.1 | 7.2 | 0.2 | 0.2 | | 9.5 | 0.3 |  |  |
| Heart valve disease non-rheumatic | 0.6 | 1.1 | 14.3 | 0.7 | 0.4 | 6.3 | 0.4 | 0.6 | | 8.5 | 0.5 |  |  |
| Hidradenitis suppurativa | 0.7 | 1.6 | 0.9 | 0.7 | 0.7 | 1.2 | 0.5 | 1.1 | | 2.1 | 0.7 |  |  |
| HIV | 0.2 | 0.1 | 0.2 | 0.1 | 0.1 | 0.1 | 0 | 3 | | 1.3 | 3 |  |  |
| Hodgkin Lymphoma | 0.2 | 0.1 | 0.6 | 0.1 | 0.1 | 0.3 | 0 | 0.1 | | 0.2 | 0 |  |  |
| Hyperparathyroidism | 0.1 | 0.5 | 1.8 | 0.2 | 0.2 | 2.2 | 0.2 | 0.2 | | 2.9 | 0.4 |  |  |
| Hyperplasia of prostate | 0.5 | 1.2 | 10 | 0.2 | 0.2 | 4.4 | 0.1 | 0.3 | | 3.1 | 0.1 |  |  |
| Hyperprolactinaemia and prolactinoma | 0.2 | 0.6 | 0.4 | 0.8 | 0.4 | 0.8 | 1.2 | 0.6 | | 1.8 | 1.9 |  |  |
| Hypertension | 7.3 | 28.2 | 70.3 | 9 | 7.5 | 54.6 | 7.1 | 13.3 | | 59.7 | 18.6 |  |  |
| Hypertrophic Cardiomyopathy | 0 | 0 | 0.6 | 0 | 0.1 | 0.3 | 0 | 0.1 | | 0.9 | 0 |  |  |
| Hypertrophy of nasal turbinates | 2.9 | 3.9 | 2.7 | 1.7 | 4.2 | 4.6 | 1.9 | 1.4 | | 2 | 1 |  |  |
| Hypopituitarism | 0.1 | 0.3 | 0.8 | 0.2 | 0.2 | 0.7 | 0.4 | 0.1 | | 0.9 | 0.2 |  |  |
| Hyposplenism | 0.2 | 0.2 | 1.5 | 0.1 | 0.1 | 0.4 | 0.1 | 0.1 | | 1.1 | 0.1 |  |  |
| Immunodeficiencies | 0 | 0.1 | 0.5 | 0 | 0 | 0.2 | 0 | 0 | | 0.3 | 0 |  |  |
| Infection of bones and joints | 0.4 | 0.7 | 4.4 | 0.2 | 0.4 | 1.9 | 0.3 | 0.5 | | 3 | 0.3 |  |  |
| Intervertebral disc disorders | 3.3 | 15.7 | 14.5 | 3.7 | 2.5 | 13.2 | 1.6 | 1.4 | | 10.1 | 1.6 |  |  |
| Intracerebral haemorrhage | 0.1 | 0.2 | 1.9 | 0.1 | 0.1 | 1.2 | 0 | 0.2 | | 1.2 | 0.2 |  |  |
| Intracranial hypertension | 0.2 | 0.4 | 0.2 | 0.2 | 0.1 | 0.3 | 0.1 | 0.3 | | 1.1 | 0.1 |  |  |
| Iron deficiency with and without anaemia | 4.1 | 12.7 | 17.9 | 5.9 | 15.8 | 37.7 | 17 | 12.6 | | 27.5 | 16 |  |  |
| Irritable bowel syndrome | 10.6 | 32.3 | 14.1 | 12.8 | 7.1 | 19.2 | 7.4 | 5.9 | | 14.4 | 5.6 |  |  |
| Juvenile arthritis | 0.1 | 0.2 | 0.2 | 0.1 | 0 | 0.1 | 0 | 0 | | 0.2 | 0 |  |  |
| Learning disability | 1.2 | 1.1 | 3.1 | 0.3 | 0.7 | 1.4 | 0.2 | 1.2 | | 2.4 | 0 |  |  |
| Leukaemia | 0.1 | 0.2 | 1.2 | 0.2 | 0.1 | 0.7 | 0 | 0.1 | | 0.5 | 0 |  |  |
| Lichen planus | 0.4 | 1.3 | 1.6 | 0.5 | 1.2 | 4.1 | 1.3 | 0.6 | | 1.8 | 0.5 |  |  |
| Liver failure and transplant | 0.1 | 0.1 | 3.8 | 0.1 | 0.1 | 1.2 | 0 | 0.1 | | 1.5 | 0.1 |  |  |
| Liver fibrosis, sclerosis and cirrhosis | 0.2 | 0.3 | 6.3 | 0.1 | 0.2 | 2.3 | 0.1 | 0.2 | | 2.3 | 0.1 |  |  |
| Lupus erythematosus (local and systemic) | 0.2 | 0.8 | 1 | 0.2 | 0.4 | 1.9 | 0.3 | 0.5 | | 3.9 | 0.4 |  |  |
| Macular degeneration | 0.1 | 0.5 | 4.1 | 0.2 | 0.1 | 1.5 | 0 | 0.1 | | 1.5 | 0.2 |  |  |
| Male infertility | 0.2 | 3.2 | 4 | 87.6 | 0.2 | 8.1 | 88.1 | 0.1 | | 6 | 85.7 |  |  |
| Meniere disease | 0.2 | 1 | 1.1 | 0.2 | 0.1 | 0.7 | 0 | 0 | | 0.5 | 0.1 |  |  |
| Menorrhagia and polymenorrhoea | 11.5 | 40 | 10.9 | 21.2 | 13.3 | 31.3 | 19.8 | 16.8 | | 34.6 | 29 |  |  |
| Migraine | 12.9 | 30.1 | 14.5 | 13.9 | 11.7 | 24.2 | 11.9 | 11.6 | | 19.3 | 10 |  |  |
| Motor neuron disease | 0 | 0.1 | 0.2 | 0 | 0 | 0.2 | 0 | 0 | | 0.1 | 0 |  |  |
| Multiple myeloma and malignant plasma cell neoplasms | 0 | 0.1 | 0.6 | 0 | 0 | 0.2 | 0 | 0 | | 0.7 | 0 |  |  |
| Multiple sclerosis | 0.4 | 1.3 | 1.2 | 0.5 | 0.3 | 0.5 | 0.3 | 0.2 | | 1.3 | 0.1 |  |  |
| Myasthenia gravis | 0 | 0.1 | 0.2 | 0 | 0 | 0.2 | 0 | 0.1 | | 0.3 | 0.1 |  |  |
| Myelodysplastic syndromes | 0 | 0 | 0.5 | 0 | 0 | 0.4 | 0 | 0.1 | | 0.4 | 0.1 |  |  |
| Nasal polyp | 1.3 | 2.7 | 3.2 | 1 | 2 | 3.1 | 1 | 1.1 | | 2.2 | 0.8 |  |  |
| Neuromuscular dysfunction of bladder | 0.5 | 3.7 | 3.7 | 0.7 | 0.6 | 4.6 | 0.4 | 0.5 | | 4.2 | 0.9 |  |  |
| Non-acute cystitis | 0.2 | 1.2 | 1.4 | 0.4 | 0.2 | 1 | 0.3 | 0.1 | | 1.1 | 0.3 |  |  |
| Non-alcoholic fatty liver disease and steatohepatitis | 0.9 | 4.3 | 8.6 | 1 | 1.9 | 9.7 | 1.5 | 0.8 | | 5.8 | 1.2 |  |  |
| Non-diabetic peripheral neuropathies (excluding cranial nerves and carpal tunnel syndrome) | 1.5 | 6 | 12.9 | 1.7 | 1.1 | 8.1 | 0.8 | 1 | | 8.1 | 1.1 |  |  |
| Non-Hodgkin Lymphoma | 0.2 | 0.3 | 1.6 | 0.2 | 0.1 | 0.7 | 0.1 | 0.2 | | 0.7 | 0.1 |  |  |
| Non-malignant tumour of brain, central nervous system and pituitary | 0.3 | 0.8 | 1.2 | 0.4 | 0.3 | 1.2 | 0.3 | 0.4 | | 1.8 | 0.7 |  |  |
| Obesity | 12 | 29.5 | 32.3 | 12.8 | 12.4 | 31.2 | 14.4 | 18.7 | | 37.9 | 22.4 |  |  |
| Obsessive-compulsive disorder | 1.4 | 2.9 | 2 | 0.9 | 0.7 | 1.2 | 0.5 | 0.4 | | 0.7 | 0.3 |  |  |
| Obstructive and reflux uropathy | 1.1 | 1.7 | 4.9 | 0.9 | 1.4 | 3.3 | 0.8 | 0.8 | | 3.2 | 1 |  |  |
| Oesophagitis and oesophageal ulcer | 4.3 | 21.4 | 23.8 | 4 | 4.7 | 23.8 | 4.2 | 3.4 | | 15.6 | 3.6 |  |  |
| Osteoarthritis | 4.2 | 29.9 | 41.4 | 6.5 | 2.5 | 31.4 | 2.6 | 3.2 | | 28.2 | 4.3 |  |  |
| Osteoporosis | 0.4 | 4.4 | 13.9 | 1.2 | 0.4 | 7.3 | 0.7 | 0.2 | | 4.2 | 0.7 |  |  |
| Other anaemias | 5.4 | 14.9 | 26 | 7.6 | 17.6 | 41.3 | 19.4 | 17.2 | | 37.3 | 21.4 |  |  |
| Other haemolytic anaemias | 0.1 | 0.1 | 0.5 | 0.1 | 0.6 | 0.9 | 0.6 | 0.9 | | 2.5 | 0.7 |  |  |
| Other interstitial pulmonary diseases with fibrosis | 0 | 0.2 | 2.3 | 0 | 0.1 | 1.4 | 0 | 0.1 | | 1.7 | 0.1 |  |  |
| Other psychoactive substance misuse | 6.7 | 6.4 | 11.3 | 2.3 | 2.4 | 3.4 | 0.5 | 4.4 | | 5.7 | 1.2 |  |  |
| Painful conditions | 9 | 50.1 | 56.8 | 10 | 11.9 | 62.3 | 11.6 | 10.8 | | 51.5 | 13.6 |  |  |
| Pancreatitis | 0.8 | 1.7 | 5.5 | 0.6 | 0.7 | 3.1 | 0.4 | 0.7 | | 3 | 0.3 |  |  |
| Parkinson's disease | 0 | 0.2 | 1.7 | 0.1 | 0 | 0.6 | 0 | 0 | | 0.4 | 0 |  |  |
| Peptic ulcer disease | 1.4 | 7 | 17.1 | 1 | 1.4 | 11.3 | 0.8 | 1.9 | | 8.3 | 1.5 |  |  |
| Peripheral arterial disease | 0.2 | 0.7 | 11.6 | 0.2 | 0.1 | 3.5 | 0.1 | 0.1 | | 3.6 | 0.1 |  |  |
| Peripheral venous and lymphatic disease | 5.7 | 12.6 | 21.7 | 6.7 | 4.5 | 14.6 | 3.9 | 2.9 | | 10.2 | 2.3 |  |  |
| Personality disorder | 2.2 | 4.7 | 6.6 | 0.7 | 0.6 | 1.6 | 0.3 | 1.3 | | 3.3 | 0.2 |  |  |
| Polycystic ovarian syndrome | 2.1 | 2.7 | 0.6 | 7.8 | 3.8 | 2.8 | 13.1 | 2.5 | | 2.3 | 7.3 |  |  |
| Polycythaemia vera | 0.1 | 0.1 | 0.9 | 0 | 0 | 0.3 | 0.1 | 0 | | 0.2 | 0 |  |  |
| Polymyalgia Rheumatica | 0 | 1 | 2.7 | 0.1 | 0 | 1.3 | 0 | 0 | | 0.7 | 0.1 |  |  |
| Portal hypertension and oesophageal varices | 0.1 | 0.1 | 4.4 | 0.1 | 0.1 | 1.4 | 0 | 0.1 | | 1 | 0.1 |  |  |
| Post-traumatic stress and stress-related disorders | 16.9 | 40.9 | 21.4 | 19.5 | 13.5 | 27.1 | 12.4 | 15.8 | | 30.7 | 17.8 |  |  |
| Primary malignancy biliary tract | 0 | 0 | 0.4 | 0 | 0 | 0.1 | 0 | 0 | | 0.1 | 0 |  |  |
| Primary malignancy bladder | 0.1 | 0.2 | 3.2 | 0.2 | 0.1 | 1 | 0 | 0 | | 1 | 0.1 |  |  |
| Primary malignancy bone and articular cartilage | 0.1 | 0.1 | 0.4 | 0.1 | 0.1 | 0.3 | 0.1 | 0.1 | | 0.4 | 0 |  |  |
| Primary malignancy brain, other CNS and intracranial | 0.1 | 0.2 | 0.9 | 0.1 | 0.1 | 0.3 | 0 | 0.1 | | 0.5 | 0 |  |  |
| Primary malignancy breast | 0.9 | 4.1 | 4.1 | 2.4 | 0.5 | 2.8 | 1.1 | 0.8 | | 3.6 | 1.2 |  |  |
| Primary malignancy cervical | 1.4 | 1.8 | 1 | 1.4 | 0.2 | 0.4 | 0.2 | 0.7 | | 0.7 | 0.3 |  |  |
| Primary malignancy colorectal and anus | 0.2 | 0.6 | 3.5 | 0.3 | 0.1 | 1.1 | 0 | 0.1 | | 1.3 | 0 |  |  |
| Primary malignancy kidney and ureter | 0.1 | 0.2 | 3.2 | 0.2 | 0.1 | 0.9 | 0 | 0 | | 1.2 | 0.1 |  |  |
| Primary malignancy liver | 0 | 0 | 0.5 | 0 | 0 | 0.2 | 0 | 0 | | 0.2 | 0 |  |  |
| Primary malignancy lung and trachea | 0.1 | 0.4 | 3.5 | 0.1 | 0 | 0.6 | 0 | 0.1 | | 1.1 | 0 |  |  |
| Primary malignancy malignant melanoma | 0.5 | 0.9 | 1.7 | 0.8 | 0 | 0.1 | 0.2 | 0 | | 0.2 | 0.1 |  |  |
| Primary malignancy mesothelioma | 0 | 0 | 0.1 | 0 | NA | NA | NA | NA | | NA | NA |  |  |
| Primary malignancy oesophageal | 0 | 0 | 1 | 0 | 0 | 0.1 | 0 | 0 | | 0.2 | 0.1 |  |  |
| Primary malignancy oro-pharyngeal | 0.1 | 0.2 | 1.3 | 0.1 | 0.1 | 0.4 | 0 | 0.1 | | 0.5 | 0 |  |  |
| Primary malignancy other skin and subcutaneous tissue | 1 | 2.7 | 8.1 | 1.8 | 0.1 | 0.4 | 0.1 | 0 | | 0.5 | 0 |  |  |
| Primary malignancy others | 1 | 3.2 | 15.4 | 1.7 | 0.5 | 4.5 | 0.6 | 0.5 | | 6.2 | 0.5 |  |  |
| Primary malignancy ovarian | 0.1 | 0.4 | 0.8 | 0.3 | 0.1 | 0.5 | 0.2 | 0 | | 0.4 | 0.1 |  |  |
| Primary malignancy pancreatic | 0 | 0.1 | 0.8 | 0 | 0 | 0.2 | 0 | 0 | | 0.6 | 0 |  |  |
| Primary malignancy prostate | 0.1 | 0.1 | 3 | 0 | 0 | 0.6 | 0 | 0.1 | | 1.1 | 0.3 |  |  |
| Primary malignancy stomach | 0 | 0.1 | 1.2 | 0 | 0 | 0.4 | 0 | 0 | | 0.4 | 0.2 |  |  |
| Primary malignancy testicular | 0.2 | 0 | 0.5 | 0.1 | 0.1 | 0.1 | 0 | 0 | | 0.1 | 0 |  |  |
| Primary malignancy thyroid | 0.5 | 1.4 | 6.8 | 0.8 | 0.4 | 1.7 | 0.3 | 0.2 | | 2.2 | 0.2 |  |  |
| Primary malignancy uterine | 0 | 0.4 | 0.7 | 0.2 | 0 | 0.8 | 0.2 | 0 | | 0.6 | 0.1 |  |  |
| Psoriasis | 5 | 7.7 | 9.1 | 4.8 | 3.4 | 6.7 | 2.5 | 1.2 | | 2.3 | 0.8 |  |  |
| Psoriatic arthropathy | 0.3 | 1.3 | 1.4 | 0.3 | 0.3 | 1.3 | 0.2 | 0 | | 0.4 | 0 |  |  |
| Ptosis of eyelid | 0.3 | 0.8 | 1.4 | 0.3 | 0.3 | 1.1 | 0.1 | 0.3 | | 1.5 | 0.1 |  |  |
| Pulmonary hypertension | 0 | 0 | 2.6 | 0 | 0 | 1.3 | 0 | 0.1 | | 3.6 | 0.1 |  |  |
| Respiratory failure | 0.3 | 0.7 | 9.7 | 0.2 | 0.2 | 3.4 | 0.1 | 0.3 | | 5.4 | 0.1 |  |  |
| Retinal detachments and breaks | 0.5 | 0.6 | 3.1 | 0.4 | 0.4 | 1.6 | 0.2 | 0.4 | | 2.6 | 0.3 |  |  |
| Retinal vascular occlusions | 0.1 | 0.2 | 1.4 | 0.1 | 0.1 | 0.8 | 0.1 | 0 | | 0.9 | 0 |  |  |
| Rheumatic valve disease | 0 | 0 | 0.3 | 0 | 0 | 0.4 | 0 | 0 | | 0.6 | 0 |  |  |
| Rheumatoid Arthritis | 0.6 | 4.3 | 6.5 | 1 | 1 | 6.8 | 1.3 | 0.7 | | 5.8 | 0.8 |  |  |
| Rosacea | 3.3 | 6.7 | 4.3 | 4.2 | 2.5 | 3.7 | 2.6 | 1.2 | | 1.8 | 0.9 |  |  |
| Sarcoidosis | 0.2 | 0.5 | 1.1 | 0.2 | 0.3 | 1.4 | 0.1 | 0.5 | | 2.4 | 0.5 |  |  |
| SARS-CoV-2 | 0.8 | 0.9 | 1.2 | 0.8 | 1.4 | 2.3 | 1.3 | 0.8 | | 1.8 | 1.1 |  |  |
| Schizophrenia and non-organic psychosis | 2.4 | 2.5 | 6.5 | 0.5 | 2.3 | 4.5 | 0.8 | 5.8 | | 8.6 | 1.2 |  |  |
| Scoliosis | 1 | 2.1 | 3.3 | 0.9 | 0.4 | 1.9 | 0.5 | 0.7 | | 2.3 | 0.3 |  |  |
| Seborrheic dermatitis | 4.9 | 9.3 | 8 | 5.1 | 7.2 | 11.7 | 5.8 | 4.1 | | 7.2 | 3.8 |  |  |
| Secondary malignancy and metastasis | 0.3 | 1.2 | 8.3 | 0.8 | 0.2 | 2.4 | 0.2 | 0.3 | | 4.3 | 0.3 |  |  |
| Secondary polycythaemia | 0.1 | 0.1 | 1.1 | 0.1 | 0.1 | 0.5 | 0.1 | 0 | | 0.3 | 0 |  |  |
| Sick sinus syndrome | 0 | 0.1 | 0.7 | 0 | 0 | 0.1 | 0 | 0 | | 0.3 | 0 |  |  |
| Sickle-cell anaemia | 0 | 0 | 0 | 0 | 0.1 | 0.3 | 0.2 | 2.2 | | 5.7 | 2.1 |  |  |
| Sjogren's disease | 0 | 0.6 | 0.6 | 0.1 | 0.1 | 1.4 | 0.1 | 0.1 | | 1.3 | 0.1 |  |  |
| Sleep apnoea | 1.1 | 3.9 | 8.2 | 0.8 | 1.2 | 6.2 | 0.7 | 1.6 | | 7.4 | 1.1 |  |  |
| Somatoform and dissociative disorders | 4 | 14.8 | 7.4 | 4.6 | 5.1 | 15.9 | 5.4 | 4.7 | | 11.7 | 5.3 |  |  |
| Spina bifida | 0.2 | 0.6 | 0.7 | 0.2 | 0.1 | 0.3 | 0 | 0.1 | | 0.4 | 0.1 |  |  |
| Spinal stenosis | 0.2 | 3.2 | 4.8 | 0.3 | 0.3 | 4.1 | 0.3 | 0.2 | | 3.6 | 0.1 |  |  |
| Spondylolisthesis | 0.2 | 1.6 | 2.1 | 0.3 | 0.1 | 1.3 | 0.1 | 0.1 | | 1 | 0.1 |  |  |
| Spondylosis | 1 | 14 | 18.5 | 1.9 | 1.2 | 15.1 | 1.1 | 0.7 | | 11.1 | 0.9 |  |  |
| Subarachnoid haemorrhage | 0.3 | 0.4 | 1.9 | 0.2 | 0.2 | 0.8 | 0.1 | 0.2 | | 0.9 | 0.2 |  |  |
| Subdural haematoma | 0.2 | 0.1 | 1.7 | 0.1 | 0.1 | 0.7 | 0 | 0.2 | | 0.9 | 0.1 |  |  |
| Supraventricular tachycardia | 0.8 | 1.5 | 4.5 | 0.7 | 0.6 | 2.2 | 0.3 | 0.4 | | 2.5 | 0.4 |  |  |
| Systemic sclerosis | 0 | 0.1 | 0.3 | 0.1 | 0 | 0.4 | 0 | 0 | | 0.5 | 0 |  |  |
| Thalassaemia | 0.1 | 0.1 | 0.1 | 0.1 | 1.5 | 2.4 | 1.4 | 1.1 | | 2.4 | 1.2 |  |  |
| Thrombocytopenia primary, secondary and other | 0.7 | 0.7 | 4.7 | 0.7 | 0.8 | 2.6 | 0.6 | 1.6 | | 3.8 | 0.9 |  |  |
| Thrombophilia | 0.7 | 1.2 | 1 | 1.4 | 0.4 | 1 | 1 | 0.4 | | 1.6 | 0.8 |  |  |
| Thyroid disease | 5 | 14.8 | 16 | 8 | 9.7 | 17.8 | 12.5 | 4.1 | | 12.3 | 5.3 |  |  |
| Tinnitus | 2.6 | 7.5 | 7.5 | 3.1 | 2.4 | 8.5 | 2.4 | 1.8 | | 6.4 | 2.9 |  |  |
| Trigeminal neuralgia | 0.3 | 2.3 | 1.6 | 0.5 | 0.3 | 1.6 | 0.3 | 0.2 | | 1.4 | 0.2 |  |  |
| Tuberculosis | 0.2 | 0.6 | 3.3 | 0.2 | 3.6 | 9.6 | 3.5 | 3.5 | | 3.8 | 3.1 |  |  |
| Type 1 Diabetes | 0.8 | 0.2 | 1.4 | 0.3 | 0.3 | 0.1 | 0.1 | 0.4 | | 0.6 | 0.3 |  |  |
| Type 2 Diabetes | 1.6 | 8.3 | 25.5 | 2.1 | 5.6 | 36.3 | 6.3 | 3.9 | | 24.7 | 5.6 |  |  |
| Ulcerative colitis | 1.3 | 1.9 | 2.5 | 1 | 1.5 | 2.7 | 0.7 | 0.6 | | 1.3 | 0.2 |  |  |
| Unspecified or Rare Diabetes | 0.4 | 0.7 | 7.3 | 0.4 | 0.8 | 6.2 | 1 | 1 | | 6 | 0.9 |  |  |
| Urinary Incontinence | 2.8 | 17.2 | 15.8 | 4.8 | 3.7 | 18.1 | 3.6 | 3 | | 16 | 3.1 |  |  |
| Urolithiasis | 3.3 | 6.7 | 11 | 2.6 | 4 | 10.1 | 2.6 | 2.1 | | 6.5 | 1.7 |  |  |
| Urticaria | 5.6 | 11.7 | 6.8 | 6.2 | 7.8 | 15.6 | 7.6 | 5.3 | | 9.4 | 5.2 |  |  |
| Venous thromboembolism | 1.9 | 4.6 | 15.9 | 1.6 | 1.1 | 6.2 | 0.7 | 1.6 | | 10.9 | 1.3 |  |  |
| Visual impairment and blindness | 1.2 | 2.6 | 7.3 | 0.9 | 1.2 | 4.9 | 0.9 | 1.4 | | 7 | 0.6 |  |  |
| Vitamin B12 deficiency with and without anaemia | 1.9 | 6.7 | 8.5 | 2.3 | 5.7 | 17.4 | 5.9 | 1 | | 4.5 | 1.4 |  |  |
| Vitiligo | 0.4 | 0.7 | 0.5 | 0.4 | 1.2 | 2.2 | 1 | 0.6 | | 1.5 | 0.9 |  |  |
| ADHD: attention deficit hyperactivity disorder, CNS: central nervous system, HIV: human immunodeficiency virus, SARS-CoV-2: severe acute respiratory syndrome coronavirus 2. | | | | | | | | | | | | |  |
