# Supplementary figures and images for: Ethnic differences in early onset multimorbidity and associations with health service use, long-term prescribing, years of life lost, and mortality: A cross-sectional study using clustering in the UK Clinical Practice Research Datalink
Source: PLoS Med. 2023 Oct 27;20(10):e1004300. doi: 10.1371/journal.pmed.1004300 (PMC10610074; doi:10.1371/journal.pmed.1004300)

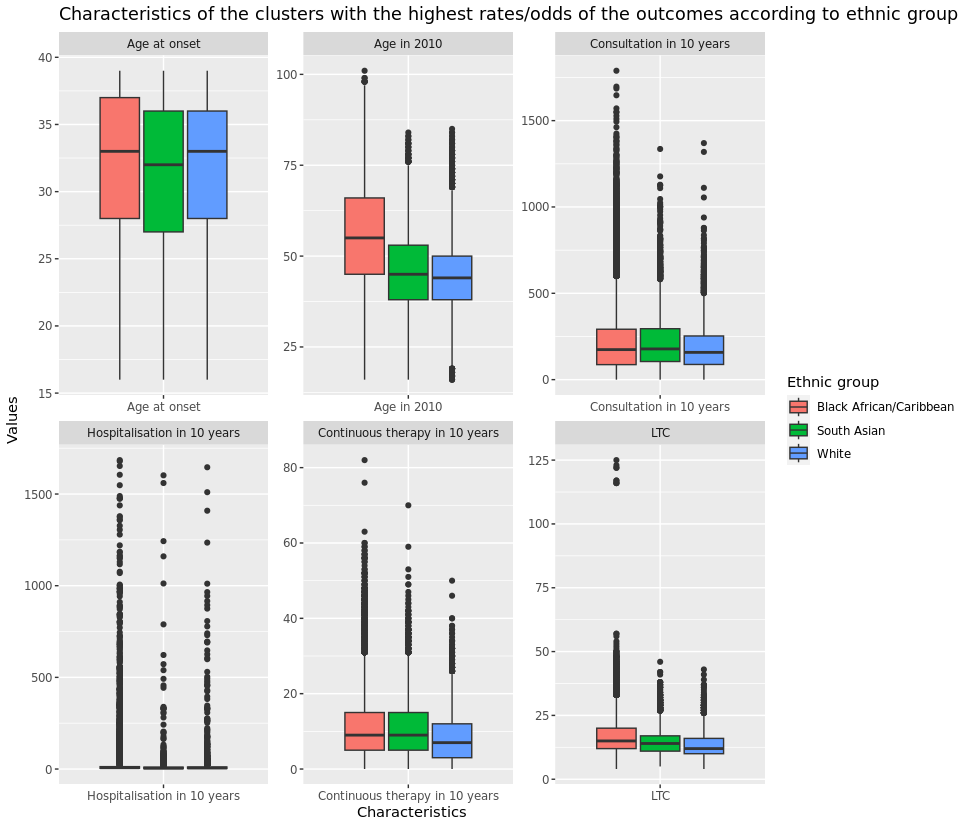

Supplement: S1 Fig — Set of boxplots showing the interquartile range, minimum and maximum values, and outliers for age at onset, age in 2010, consultation in 10 years, hospitalisation in 10 years, continuous therapy in 10 years, and LTC. (PNG) [file pmed.1004300.s007.png]

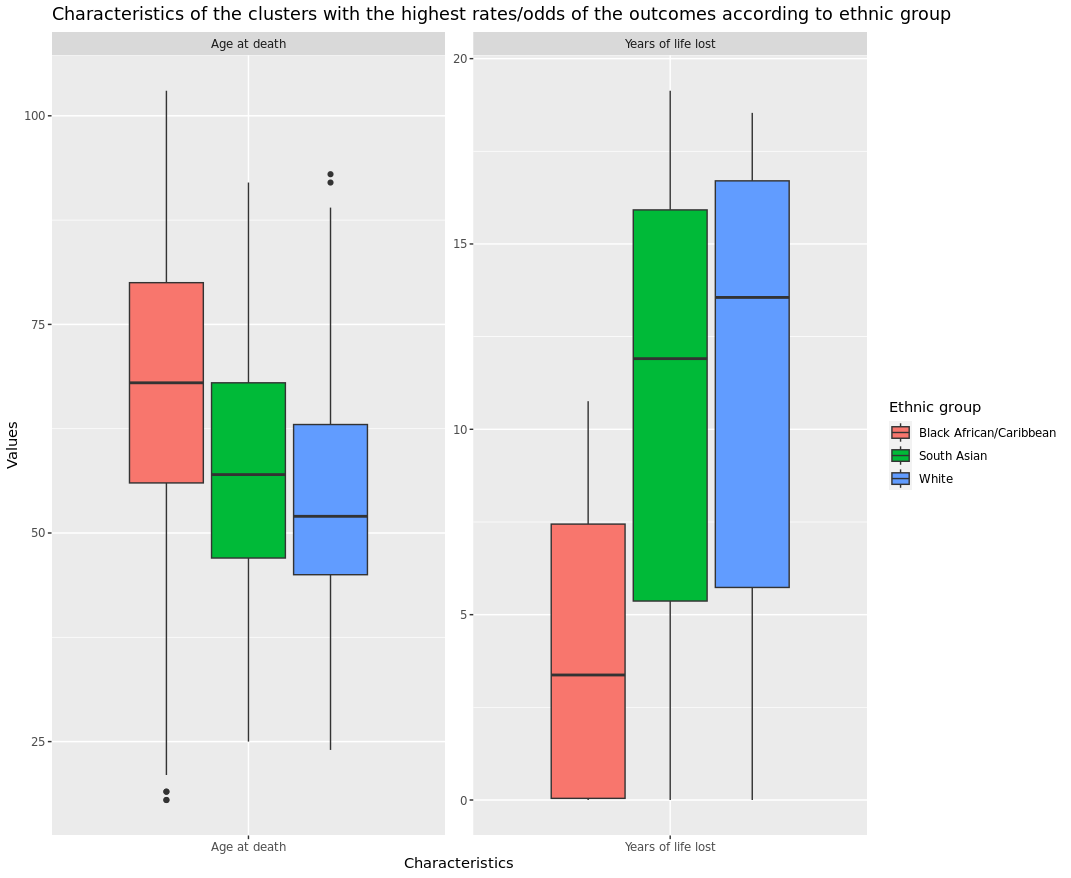

Supplement: S2 Fig — Boxplots showing the interquartile range, minimum and maximum values, and outliers for age at death and YLL. (PNG) [file pmed.1004300.s008.png]

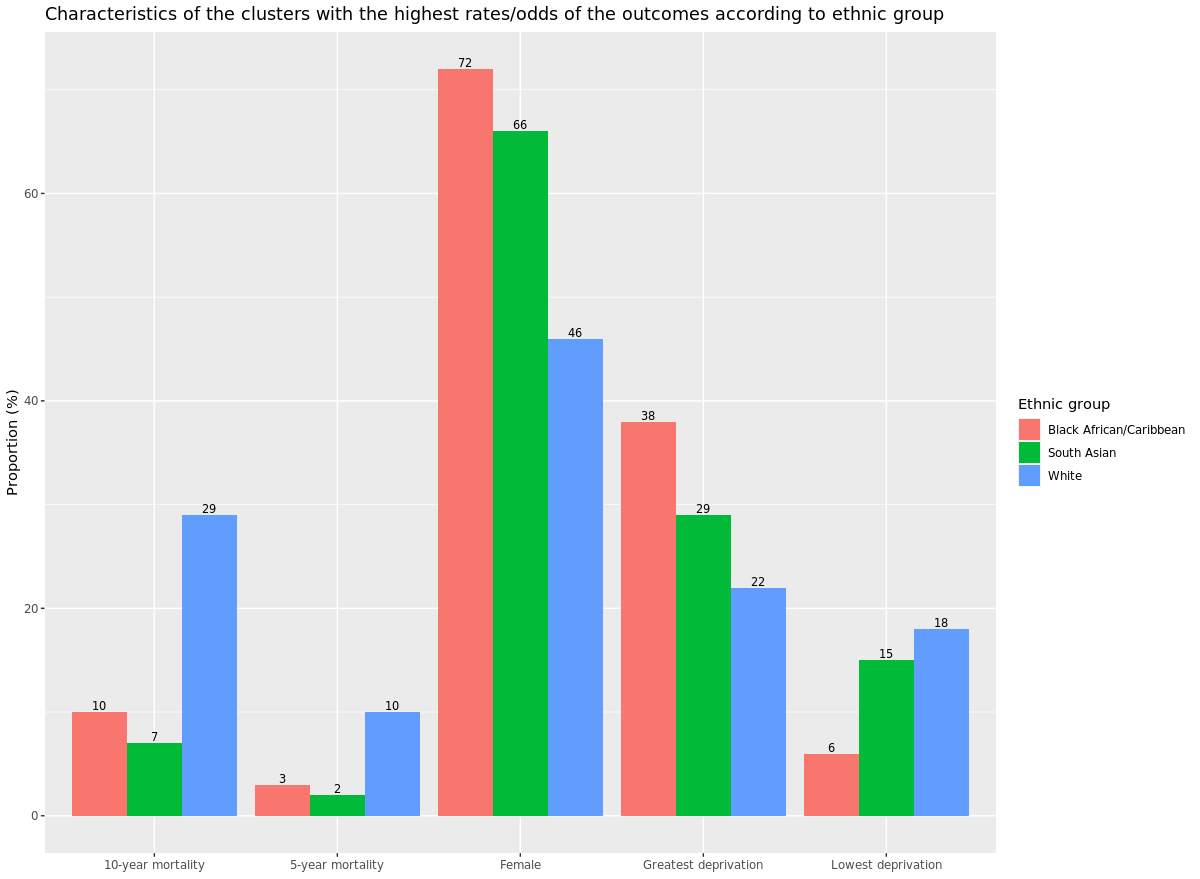

Supplement: S3 Fig — Histogram showing the proportion of people who died in the year 10 and 5, the proportion of females and people at the greatest and lowest socioeconomic deprivation levels. (PNG) [file pmed.1004300.s009.png]
