# Supplementary material for: Ethnic differences in early onset multimorbidity and associations with health service use, long-term prescribing, years of life lost, and mortality: A cross-sectional study using clustering in the UK Clinical Practice Research Datalink
Source: PLoS Med. 2023 Oct 27;20(10):e1004300. doi: 10.1371/journal.pmed.1004300 (PMC10610074; doi:10.1371/journal.pmed.1004300)
Supplement: Attachment — Submitted filename: Response letter.docx [file pmed.1004300.s010.docx]

Dear PLoS Medicine editors,

We truly appreciated your positive comments and the helpful suggestions from the reviewers. We strongly believe that thanks to all the valuable questions, comments and suggestions, we were able to enhance this revised version of our manuscript and make it clearer and more concise.

We have addressed the specific points made by each reviewer and the editors in this response letter and edited the manuscript, tables, figures and supporting information files accordingly. We also pointed out the changes we have made in the manuscript and included an excerpt of the revised text and the location where each change can be found.

You will appreciate that we accepted the challenge from one of the reviewers to make the manuscript more concise and hence managed to cut out approximately 800 words from this revised version. For this reason, a great part of the revision throughout the text was done to address the challenge of tightening up the wording and making the entire paper more concise, and therefore, it may not be specifically mentioned in the response to the reviewers.

Please, note that we have made a couple of important changes aiming to facilitate the logic of the comparisons between clusters. Firstly, we are no longer using the abbreviations ‘HRC’ (high risk cluster) and ‘LRC’ (lowest risk cluster), instead, we are referring to the clusters by either their leading conditions or their label. We believe that this is a more informative way of referring to the clusters in general and facilitating the logic of making comparisons between them. Secondly, we changed the numbering of the clusters so that cluster 1 is now always the ‘lowest impact’ (on the outcomes) cluster and cluster 3 or 4 is always the ‘highest impact’ cluster. As a consequence of this change, former clusters 1, 2 and 3 are now clusters 2, 3 and 1, respectively, for the South Asian and Black African Caribbean groups. Likewise, former clusters 1, 2, 3 and 4 are now clusters 2, 3, 4 and 1 for the White group. We have edited the cluster labels in the relevant tables, figures and throughout the manuscript.

Furthermore, we would appreciate it if the following information could be added by the end of the manuscript: Disclaimer, Data Sharing and Acknowledgements. They can be found at the end of this document and at the end of the reviewed manuscript.

Thank you very much again for the opportunity to respond to the reviewers’ comments.

We look forward to hearing from you at your earliest convenience.

Yours sincerely,

Dr Fabiola Eto

**Requests from the editors**

**Request 1:**

**“In the abstract (Methods and findings subsection), please quote aggregate demographic and ethnic details for study participants.”**

Thank you for suggesting the inclusion of this relevant information. We have included demographic and ethnic details for the study participants as suggested:

Abstract section (page 2, lines 50-51): “*The study population included 777,906 people of White ethnicity (93%), 33,915 people of South Asian ethnicity (4%) and 26,048 people of Black African/Caribbean ethnicity (3%).”*

**Request 2:
“Where feasible, please quote numbers of participants along with the corresponding percentages in the abstract.”**

Thank you for your suggestion. We have included the numbers of participants along with the corresponding percentages in the abstract as follows:

Abstract section (page 2, lines 53-55): *“We found that early onset multimorbidity was more common among South Asian (59%, 33,915), and Black (56% 26,048), groups compared to the White population (42%, 777,906).”*

Abstract section (page 3, line 58): *“The White population was predominantly male (54%, 44,150) (…)”*

Abstract section (page 3, lines 61-63): *“At the end of the study, 4% (34,922) of the White early onset multimorbidity population had died compared to 2% of the South Asian and Black early onset multimorbidity populations (535 and 570, respectively) (…)”*

**Request 3:
“Please add a new final sentence to the 'Methods and findings' subsection of your abstract, which should begin "Study limitations include ..." or similar and should quote 2-3 of the study's main limitations.”**

Thank you so much. We have added a new final sentence that mentions some of the limitations of our study as suggested:

Abstract section (page 3, lines 66-69): *“Study limitations include the exclusion of individuals with missing ethnicity information, the age of diagnosis not reflecting the actual age of onset and the exclusion of people from Mixed, Chinese and other ethnic groups due to insufficient power to investigate associations between multimorbidity and health-related outcomes in these groups.”*

**Request 4:
“After the abstract, please add a new and accessible, tripartite 'Author summary' section in non-identical prose. You may find it helpful to consult one or two recently published research papers published in PLOS Medicine to get an impression of the preferred style.”**

We have added this as requested. Please, see Author summary section (page 3, line 75-82 and page 4, lines 83-103):

**Author summary**

**Why Was This Study Done?**

•	Most studies of multimorbidity focus on older adults and only a few have investigated multimorbidity in younger populations.

•	The prevalence of multimorbidity is increasing among young adults, particularly in minority ethnic groups and individuals living in socioeconomically deprived areas.

•	There is evidence showing that individuals with socioeconomic vulnerability experience poorer health outcomes, such as lower quality healthcare provision, premature death and higher mortality rates.

•	The association between early onset multimorbidity and poor health outcomes in ethnically and socially diverse populations in England remains under-investigated.

**What Did the Researchers Do and Find?**

•	We used primary and secondary healthcare electronic health records from 837,869 individuals of White, South Asian, and Black African/Caribbean ethnicity in England with early onset of multimorbidity who were registered with an English general practice between 2010 and 2020.

•	We found that the early onset of multimorbidity was more common among minority ethnic groups (59% and 56%, in the South Asian and Black populations, respectively) than in the White population (42%) living in the UK.

•	South Asians and Black individuals with early onset multimorbidity died earlier than White individuals with early onset multimorbidity (52 and 48 years old in the median, respectively, vs. 61 years old).

•	South Asian and Black groups were more socioeconomically deprived than White groups, with a consistent deprivation gradient across all multimorbidity clusters. In White groups, the cluster of individuals with the highest rates/odds of the outcomes was more socioeconomically deprived than the cluster with the lowest rates/odds of the outcomes.

**What Do These Findings Mean?**

•	Our findings emphasise the need to identify, prevent and manage multimorbidity early in the life course.

•	Our work highlights the need to ensure that public health policies are equitable and reach those living in socioeconomic deprivation and minority ethnic groups who are disproportionately and more severely affected by early onset multimorbidity.

**Request 5:**
**“In the Methods section (main text), please state whether or not the study had a protocol or prespecified analysis plan, and if so attach the relevant document(s) as supplementary file(s), referred to in the text,”**

Thanks for this request. We have included the relevant information in the methods section. Please, see below:

Methods section (page 7, lines 163-165): *“The use of CPRD data for this study was approved by the Independent Scientific Advisory Committee for the Medicines and Healthcare products Regulatory Agency and the study followed a pre-specific analysis plan (see S1 Protocol and S1 Checklist).*

**Request 6:
“We suggest moving the participant flowchart to the main body of the paper.”**

We have moved the participant flowchart to the main body of the paper by including its caption.

**Request 7:
“Noting referee 1's comments on p values, we ask that these remain in the relevant table in accord with PLOS Medicine policy.”**

Thank you for this observation. The p-values will remain in the relevant table.

**Request 8:
“Please restructure the early part of the Discussion (main text): the first paragraph should consist predominantly of a summary of the main findings, and other elements can appear in subsequent paragraphs.”**

Thank you for this request. We have changed the order of the paragraphs accordingly. The second paragraph, which presents a summary of the main findings, is now the first paragraph of the discussion, and the former first is now the second paragraph. Please, see the changes below:

Discussion section (page 20, lines 373-376 and page 21, lines 377-388):

*“Our findings show that in a large, multimorbid UK population, approximately 40% developed multimorbidity early (aged 16-39 years). Black and South Asian populations were more likely to become multimorbid early than Whites. We built on these findings to demonstrate the impact of early onset multimorbidity using measures of healthcare utilisation (primary care consultations, hospitalisation), long-term prescription use, mortality and years of life lost. In doing so, we have demonstrated for the first time that South Asian and Black groups with early onset multimorbidity died younger and lost more years of life once they become multimorbid compared to the White group. Although it is well established that multimorbidity increases with age [4,7,35], we show that multimorbidity is highly prevalent in younger populations and disproportionately affects minority ethnic and socially deprived groups, highlighting the need for early interventions to prevent and manage multimorbidity in those populations.*

*To our knowledge, this is the first study to investigate early onset of multimorbidity and its variation by ethnicity in a large UK population-based sample. By applying a data-driven approach across multiple long-term conditions, and combining this with measures of healthcare utilisation, mortality and years of life lost, we have built new understanding of the burden and impact of early onset multimorbidity at population scale. Our findings provide important justification to improve the prevention, recognition and management of multimorbidity in young and diverse populations.”*

**Request 9:
“Noting reference 1 and others, please list an individual or group author name or names first, as for the scholarly references.”**

We have edited the relevant references as requested. Please see:

References section (page 27, lines 550-559):

*The Academy of Medical Sciences. Multimorbidity: a priority for global health research. | The Academy of Medical Sciences. [cited 10 Jun 2020]. Available: https://acmedsci.ac.uk/file-download/82222577https://acmedsci.ac.uk/policy/policy-projects/multimorbidity*

*National Institute for Health and Care Research [Internet]. Artificial Intelligence for Multiple Long-Term Conditions (AIM) - Research Specification. [cited 5 Dec 2022]. Available: https://www.nihr.ac.uk/documents/artificial-intelligence-for-multiple-long-term-conditions-aim-research-specification/24646*

*UK Research and Innovation [Internet]. Multimorbidity or multiple long-term conditions (MLTC). [cited 5 Dec 2022]. Available: https://www.ukri.org/what-we-offer/browse-our-areas-of-investment-and-support/multimorbidity-or-multiple-long-term-conditions-mltc/*

References section (page 29, lines 655-658):

*Public Health England [Internet]. Beyond the data: COVID-19: Understanding the impact on BAME communities. [cited 2 Mar 2023]. Available: https://assets.publishing.service.gov.uk/government/uploads/system/uploads/attachment_data/file/892376/COVID_stakeholder_engagement_synthesis_beyond_the_data.pdf*

**Request 10:
“Noting references 9 & 10, for example, please use the journal name abbreviations "PLoS ONE." and "PLoS Med.".”**

Thanks for noticing the need for the standardisation of these abbreviations. We have updated the references accordingly. Please, see details below.

References section (page 27, line 575-578):

*Khanolkar AR, Chaturvedi N, Kuan V, Davis D, Hughes A, Richards M, et al. Socioeconomic inequalities in prevalence and development of multimorbidity across adulthood: A longitudinal analysis of the MRC 1946 National Survey of Health and Development in the UK. PLoS Med. 2021;18: e1003775. doi:10.1371/journal.pmed.1003775*

References section (page 27, line 579-581):

*Stafford M, Knight H, Hughes J, Alarilla A, Mondor L, Pefoyo Kone A, et al. Associations between multiple long-term conditions and mortality in diverse ethnic groups. PLoS ONE. 2022;17: e0266418. doi:10.1371/journal.pone.0266418*

References section (pages 29 and lines 645-648):

*Plana-Ripoll O, Canudas-Romo V, Weye N, Laursen TM, McGrath JJ, Andersen PK. lillies: An R package for the estimation of excess Life Years Lost among patients with a given disease or condition. PLoS ONE. 2020;15: e0228073. doi:10.1371/journal.pone.0228073*

References section (page 30, line 674-676):

*Mathur R, Farmer RE, Eastwood SV, Chaturvedi N, Douglas I, Smeeth L. Ethnic disparities in initiation and intensification of diabetes treatment in adults with type 2 diabetes in the UK, 1990–2017: A cohort study. PLoS Med. 2020;17: e1003106. doi:10.1371/journal.pmed.1003106*

**Request 11:
“Please add a completed checklist for the most appropriate reporting guideline, e.g., STROBE, labelled "S1_STROBE_Checklist" or similar and referred to in the Methods section (main text). In the checklist please refer to individual items by section (e.g., 'Methods') and paragraph number, but by line or page numbers as these generally change in the event of publication.”**

Thank you very much for requesting a checklist. We have added a completed RECORD statement as a supplementary file (S1 Checklist). The RECORD statement is a “checklist of items, extended from the STROBE statement, that should be reported in observational studies using routinely collected health data.”

In addition, we have referred to the RECORD statement in the Methods section as requested:

Methods section (page 7, lines 163-165): “*The use of CPRD data for this study was approved by the Independent Scientific Advisory Committee for the Medicines and Healthcare products Regulatory Agency and the study followed a pre-specific analysis plan (see S1 Protocol and S1 Checklist).”*

**Reviewer #1**

**Comment 1:**

**“I was just after some clarity with regards to the lookback period for multimorbidity and cohort inception. It looks as though cohort inception was at the beginning of 2010 - was all available data used for the lookback period to ascertain multimorbidity? What potential is there from the CPRD to get misclassified condition information if there are different periods of lookback available for participants with different ages at cohort inception?”**

Thank you for your question. Indeed, this information should be added to the text for clarification. That is correct, all retrospective available data were used to ascertain multimorbidity. When there are different periods of lookback available for participants with different ages at cohort inception, the potential to get misclassified condition information might occur as some of the limitations are intrinsic to the use of electronic health records. We recognise that the number of LTCs identified is related both to the frequency of healthcare service use as well as the length of healthcare service use in one’s life period (e.g. the longer one has been using the healthcare service the more opportunities to have multimorbidity detected they will have). However, it is worth mentioning that we adjust the analyses of associations between clusters and the outcomes by age at 2010, which might address some of this bias.

We believe we have mentioned a similar line of thought in the “Strengths and limitations” section (page 24, lines 482-488):

*“There are some limitations intrinsic to the use of electronic health records. The health conditions selected might be subject to misclassification due to unrecorded, miscoded, and undiagnosed diseases. The age of onset might not reflect the actual age at which a given condition was diagnosed, but the date when it was entered in the patient’s medical record. Additionally, the number of LTCs identified is related to the use of the health care service, as well as the duration of data availability within their electronic health records. Individuals with data recorded for longer periods have had time and opportunity to have multimorbidity detected.”*

Following your comment, we have included the information about the use of all available data, as follows:

Methods section (page 7, lines 183-185): “*Using all data available from the individual’s primary care and secondary care health records we identified each LTC using the first relevant code ever recorded (…)*”

Methods section (page 8, lines 196-198): *“LTCs were identified using all available data in the individual’s electronic health record, spanning from as far back as 1920, through to 2020.”*

**Comment 2:**

**“I wasn't clear whether condition information was from information collected during hospitalisation episodes or diagnoses recorded on the EHR by GP/specialists. Were all EHR records used to get these diagnoses?”**

We are sorry that this wasn’t clear. All available data from the individuals’ EHR were used to get the diagnosis, both from primary (e.g. GP consultations) and secondary care (e.g. hospitalisations). We hope that that information became clearer by stating that in the methods.

Methods section (page 7, lines 183-185): “*Using all data available from the individual’s primary care and secondary care health records we identified each LTC using the first relevant code ever recorded (…)*”

**Comment 3:**

**One limitation is the interpretation of the coefficients from the multivariable adjusted analysis. The focus of this manuscript seems to be on differences between the clusters in the outcomes, but depending on the causal relationship between the other covariates the same interpretation can't be made of the coefficients from the other covariates, i.e. Table 2 fallacy.**

We appreciate your comment on this limitation. In fact, the manuscript focuses on the different associations between clusters and outcomes but also considers the direct effect of socioeconomic deprivation on the rates and odds of the outcomes assuming that the other variables in the model (clusters, sex and age) are held constant in that relationship. This assumption was not mentioned in the description of the results, but in order to address the Table 2 fallacy, by distinguishing the interpretation of the causal effects, we included this after the description of the associations between socioeconomic deprivation and outcomes. Please, see below:

Results section (page 20, line 370): *“These associations assume that the other covariables in the regression model are held constant.”*

**Comment 4:**

**Most of the manuscript is written without focusing on p-values for inference, I think this is a good approach given the sample size and would suggest the p-value indicators be taken from Table 3.**

We thank you for this observation and suggestion. However, in order to meet PLoS Medicine policy and in addition to the editor's request, the p-values must remain in Table 3.

**Comment 5:
Page 5, Paragraph 4. I would typically test a '1 class model', and it is possible that this is the best fitting number of classes (i.e. there are no latent classes). Can I confirm that the 1 class model was not a better fit than any of the models with >=2 classes?
What criteria was used to decide on the upper limit to the number of classes?
Was the criteria for selecting number of classes (at a point where there is relatively small changes in BIC etc. and an improvement in entropy relative to adjacent number of classes) decided a priori or was this decided after seeing the fit indices?**

Thank you for such relevant comments. In some of the initial analyses, we attempted to test a 1-class model for each ethnic group, but unfortunately, we could not get the 1-class model to converge for the White group due to the large population size and the fact that the job had a maximum time limit of 240 hours to run in our university’s High-Performance Computing cluster. However, after the support of the IT Research team, a few solutions were tested, and they managed to expand the original poLCA R library creating the poLCAParallel R package that allows the parallelisation of the code starting from a minimum 2-class model. After using their solution, we obtained models that successfully converged.

After testing different numbers of classes, the upper limit to the number of classes was discussed between some of the authors considering a balance between i) obtaining models that accurately defined classes (entropy values as close as possible to 1), ii) a minimum number of classes that presented small relative changes in BIC/SABIC between the k and k-1 models being tested, and finally iii) parsimony –we wanted to test enough number of classes that would allow obtaining the simplest model as possible that would preserve homogeneity within clusters and heterogeneity between clusters.

The criteria for selecting the number of classes were defined a priori in accordance with the literature on model selection criteria for latent class analysis. Those criteria were examined every time we tested different number of classes.

**Comment 6:
Page 6, Paragraph 1. For reference class, could this be better described as the lowest rate of outcome?
What criteria was used to decide the neg-bin and ZIP models for the different outcomes? Did the residuals from these models indicate an appropriate distribution (this might be hard to see with your sample size!).**

We thank you for that suggestion. Indeed, the “lowest rate of the outcomes” describes better the criteria to select the reference class. We have edited the text accordingly.

Methods section (page 10, lines 240-241): *“For each outcome of interest, the cluster with the lowest frequency of the outcomes was considered the reference group.”*

Analysing the distribution of the discrete outcomes (e.g. number of consultations, number of hospitalisations and counts of unique continuous prescriptions in a year interval) we found overdispersion in our data –the conditional variance exceeded the conditional mean (that was further tested by dividing the Poisson model’s residual deviance by the degrees of freedom). In addition, hospitalisation and counts of prescriptions presented an excess of zeros, given that several individuals did not have hospitalisation episodes or use continuous medication throughout the 10-year period analysed.

We tested and compared both Negative Binomial and Zero-Inflated Poisson models using the Vuong Non-Nested Hypothesis Test-Statistic, which is a likelihood–ratio-based test for model selection, that under its null hypothesis, both models are adequate to explain the variations of the exposure on the outcome. When the null hypothesis was rejected (p < 0.05) we understood that the Zero-Inflated Poisson model was superior to the Negative-Binomial model, and was therefore, selected to be presented in our results.

The evaluation of the residuals for the models was inconclusive due to the sample size.

**Comment 7:
Page 7, Paragraph 2. Is there an overall descriptor that could be used to distinguish within high/low clusters? This might not be possible but if there is, it does make the analysis using the LC class as an exposure easier to follow.**

Thank you for your comment. Following your and other reviewers’ suggestions, we replaced the descriptors HRC (high risk cluster) and LRC (low risk cluster) with either the clusters’ leading conditions or their labels in the text. We believe that this is a more informative way of referring to the clusters in general and facilitating the logic of making comparisons between them. Please, see an example of the replacement below:

Results section (page 14, lines 319-321): *“For the White group, the cluster with the highest rates/odds of outcomes was led by hypertension, depression and painful conditions (cluster 4) while the cluster with the lowest rates/odds of outcomes was led by female infertility, male infertility and depression (cluster 1).”*

**Comment 8:
Figure 1. It looks like the order of conditions on the y-axis is just in alphabetical order - could a meaningful order such as overall prevalence be used to order this axis to make it more meaningful?**

Thank you for your suggestion. We have experimented with that alternative, but we agreed that the alphabetic order makes it simple and more logical to follow when comparing the prevalence of a given LTC across ethnic groups. Let us explain it further. Because the LTCs prevalence varies across ethnic groups (and these differences are one aspect that makes the results interesting) we would have to choose an ethnic group to order the prevalence by, but a given LTC with the highest prevalence in ethnic group X might not be the highest prevalent one in ethnic group Z. For example, “iron deficiency anaemia” is 100% prevalent (e.g. prevalent in all clusters) in the South Asian and Black groups, but not prevalent in any clusters of the White group. The usage of an overall prevalence to sort the LTCs would not reflect the prevalence variations between clusters within and between ethnic groups, precisely what we would like to make visible with this plot.

**Comment 9:
Table 3+4. Is Cluster 1 a 'medium risk' cluster?**

We appreciate that observation. We would say that, specifically for the mortality outcomes, (former) cluster 1 (current cluster 2 after revision) could be considered a “medium risk” cluster, especially for South Asians and Blacks, and also for Whites regarding the odds of mortality in the 10th year. However, when we consider the outcomes related to healthcare service use, (former) cluster 1 presents slightly lower rates in regards to the “lower risk” clusters across ethnic groups.

**Reviewer #2**

**Comment 1:**

**“Overall, this piece is incredibly lengthy. I would challenge the authors to perhaps find areas in which they could be more concise. Importantly, for example, the conclusion should be shortened and should not contain hypotheses. Instead it should focus on summarizing the work at hand.”**

Thank you so much for your comment. The co-authors and I took your challenge and have gone through the manuscript to tighten up the wording and make the entire paper more concise. We have managed to cut out approximately 800 words. Additionally, we have rephrased the statement not to mention any hypothesis. Please, see below:

Conclusion section (page 25, lines 496-497*): “It is likely that the worse outcomes from early onset multimorbidity in minority ethnic and socioeconomically deprived groups (…)”*

**Comment 2:**

**“Acronyms: Throughout the manuscript there are several acronyms that made it hard to follow. "LTC" has varied meanings and in the US they are referred to as "chronic conditions." While this may not be the authors preferred term, I would encourage not using the LTC acronym given the use of LCA. Similarly, HRC and LRC seem like unnecessary acronyms that made it hard to follow. Finally, please note "IMD" as an acronym in the "Covariables" section.”**

Thank you for this suggestion. We appreciate the challenge of readability of unfamiliar acronyms. However, there is a difficult balance to achieve between a manuscript that is concise (with acronyms) versus a longer version without acronyms. We are conscious that different journals have a varied approaches to the use of acronyms, and we are very happy to take an editorial steer on our use of them to ensure our approach is consistent with PLoS Medicine policy.

Because we use the term LTC (long-term condition) more than 60 times over the entire text, we understand that it would be more sensible to use the acronym for this particular case and conserve a more concise text. However, we replaced the acronyms HRC (high risk cluster) and LRC (low risk cluster) with either the clusters’ leading conditions or their label in the text. Additionally, we removed the LCA acronym and instead stated the full term – latent class analysis.

Please, see an example of the replacement below:

Results section (page 14, lines 319-321): *“For the White group, the cluster with the highest rates/odds of outcomes was led by hypertension, depression and painful conditions (cluster 4) while the cluster with the lowest rates/odds of outcomes was led by female infertility, male infertility and depression (cluster 1).”*

**Comment 3:**

**“In the United States, the groups would be referred to as "race" and not "ethnicity." While I understand this social construct varies across countries, I just want to make sure this is consistent with the terminology expected by an English audience. For example, is the variable labelled as ethnicity? Similarly, terms such as "Blacks" should not be used.“**

We appreciate your comment and concern about this rather controversial terminology. The term “ethnicity” is widely disseminated in scientific studies developed in the United Kingdom and its [national institutions](https://www.ethnicity-facts-figures.service.gov.uk/style-guide/ethnic-groups), such as the [Office for National Statistics (ONS)](https://www.ons.gov.uk/peoplepopulationandcommunity/culturalidentity/ethnicity/bulletins/ethnicgroupenglandandwales/census2021) and the [National Health Service (NHS)](https://digital.nhs.uk/data-and-information/data-collections-and-data-sets/data-sets/mental-health-services-data-set/submit-data/data-quality-of-protected-characteristics-and-other-vulnerable-groups/ethnicity).

We used the terminology “ethnicity” to refer to different ethnic groups analysed in our study. The ethnic groups were re-labelled as White, Black African/Caribbean and South Asian. We followed the list of ethnic groups available in the database provided by Clinical Practice Research Datalink GOLD used in our study. The ethnic groups mentioned are self-report information obtained from the patient’s electronic health records and captured during primary care registration and/or consultation.

We have revised the wording throughout the manuscript to ensure appropriate descriptions of our populations such as “People of Black ethnicity” or “Black ethnic groups” etc.

**Comment 4:**

**“It is unclear, in the introduction, methods, and discussion, what is meant by the fact that most multimorbidity studies focus on diseases. Please elaborate what this means conceptually and methodologically.”**

Thank you for realising this inconsistency. We wanted to refer specifically to studies on *clusters* of multimorbidity instead of multimorbidity studies more generally. What we wanted to mean is that the majority of studies on *clusters of multimorbidity*, especially when choosing their analysis technique, focus on diseases (as observation in their analysis) rather than individuals to assess multimorbidity patterns. Those studies usually focus and build up their discussion around groups or clusters of diseases that commonly co-occur. On the other hand, studies that focus on individuals as the main observation in their analysis will look for groups of individuals or clusters of individuals who share similar characteristics, including the diseases they have in common and commonly co-occur. In those studies, the discussion will be mostly around the profile of individuals within and between clusters, and what are their similarities and differences in terms of the variables analysed, in our case, socioeconomic characteristics and long-term conditions.

We hopefully have made it clearer in the introduction, methods and discussion trying not to be much extensive. Please see the edits and underlined insertions below:

Introduction section (page 5, lines 127-128): *“The majority of studies mapping patterns of multimorbidity focus on diseases as the unit of analysis rather than individuals.”*

Methods section (page 8, lines 193-194): *“To focus on individuals rather than diseases as the observation in the analysis, we applied latent class analysis (…)”*

**Comment 5:**

**“I am not convinced that the statement "is more likely to reveal underlying disease relationships within clusters" is entirely accurate. I'm not sure that a data driven approach, including LCA, will truly do this from a conceptual perspective.”**

We thank you for bringing up this statement into question. Re-reading the entire statement in the text we realised that, from a conceptual perspective, not only a data-driven approach could reveal underlying disease relationships within clusters. Thus, we decided to remove this statement from the text.

**Comment 6:**

**“Could the authors comment on if there were ethnic groups that were not included? Other than those with missing.”**

The following ethnic groups were not included in the study analysis: Mixed and Chinese or other group. The reason was the small population size within each ethnic group that fulfilled our study eligibility criteria, resulting in inadequate power to derive robust clusters of multimorbidity and examine associations with health outcomes and mortality.

We have included this information in the Flow chart (Fig 1 was moved to the main body of the paper upon request from the editors) for the selection of our study population, as well as in our study limitations.

Abstract section (page 3, lines 66-69): *“Study limitations include the exclusion of individuals with missing ethnicity information, the age of diagnosis not reflecting the actual age of onset and the exclusion of people from Mixed, Chinese and other ethnic groups due to insufficient power to investigate associations between multimorbidity and health-related outcomes in these groups.”*

Strengths and limitations (page 24, lines 478-481): *“Besides, other ethnic groups were not included in the analysis, for instance, Mixed and Chinese or other group. The reason was the small population size within those ethnic group that fulfilled our study eligibility criteria, resulting in inadequate power to derive robust clusters of multimorbidity and examine associations with health outcomes and mortality.”*

**Comment 7:**

**“I have some trepidation with the use of YLL as an outcome. I think, while it is certainly interesting, there are perhaps some methodological limitations in this estimation. Given that bootstrapping is mentioned, is this calculated based on the sample and the distribution of individuals who were known to have died? Which is just 2-4%. If the authors wish to keep this in I think a more clear walk through of this estimation and discussion of the limitations is needed.”**

We appreciate your comment. The YLL was calculated using data on the entire multimorbid population within each cluster (age at onset, age at death, expected life expectancy at birth for the UK population, and live status (alive or dead)). We obtained the YLL and respective confidence intervals considering an age-weighted estimate for the whole group. The small mortality rate reflects the young population investigated, but we believe we have enough sample size to obtain the respective estimates. Even though the authors did not present any limitations to the method, we recognise the method is not free of limitations –it is computationally expensive (especially the confidence intervals) to run depending on the number of iterations specified and might have other limitations depending on the research question to be investigated.

We did not delve deeply into the details given the scope and audience of the journal, however, to whom it may concern, more details on the methods used to estimate YLL are available in the source paper cited in the Methods section (page 9, line 231).

**Comment 8:**

**“While we consider long-term prescribing perhaps a negative outcome generally, perhaps in this context it is a positive outcome? Given that the sample was limited to those with early onset multimorbidity is prescribing a sign of appropriate care? Further, given that the types of medications were not examined it's hard to say if this is truly a bad measure. Perhaps it is a marker of disease severity but is similarly a marker of appropriate treatment. I would challenge the authors to think a bit more about this in the context of this work.”**

We appreciate your thoughts and comment on this subject. In our study, the analysis of long-term prescribing was used as a proxy measure of health service utilisation, and also as a marker of having a long-term condition (that requires treatment), which does not exclude a sign of possibly appropriate care. However, a limitation of the use of prescription data in the context of primary health care is that being prescribed a medication does not necessarily guarantee that the patient made proper use of it throughout time. In the context of the presented study, the types of medications used were not relevant to inform health service utilisation; however, it would certainly be very informative should we want to investigate the associations between the types of medication prescribed and the clusters of individuals with a group of highly prevalent long-term conditions.

**Comment 9:**

**“Figure 1: Given that, based on my understand, the % being represented is out of 4 (White) or 3, I'm not sure the continuous scale makes the most sense. Would some type of discrete scale be more applicable here? While I understand what the figure is conveying, seeing as it's not truly a continuous measure the shading of the colors is a bit more challenging to follow.”**

We thank you for your comment on Figure 1. Originally, we made the figure using a discrete scale and showing the counts of clusters where each LTC appeared. However, as the ethnic groups presented a different number of clusters, we realised it would not make sense to show a discrete scale when we have different ranges by groups. To exemplify what we mean let us take a look at the figure below using a discrete scale, but in shades of black/grey. We can see that even though some LTCs (e.g. painful conditions, obesity, dermatitis, depression, constipation, etc.) appeared among the most prevalent LTCs in all clusters from the three ethnic groups, it displays a different shade in the plot according to the number of clusters in each ethnic groups – it is black for the White group, but dark grey for the South Asian and Black groups. We concluded that, because they have different ranges of clusters, this difference in shades was not accurately demonstrating the similarities and differences between ethnic groups. Therefore, we decided to use the figure that uses a continuous proportional scale, in order to make the groups comparable.

**Reviewer #4**

**Comment 1:**

**Introduction. The second last paragraph may not be necessary in this section and extra detail could be consolidated with additional methods description or moved to supplementary.**

Thank you for this suggestion, we agree with you and removed the second paragraph of the introduction from the text.

**Comment 2:**

**Methods. Could some of the detail be made more concise in the main text and the full description be moved to supplementary, for example paragraph two, and details about the process used to defined multimorbidity.**

Thank you for your suggestion. We appreciate the challenge of conciseness in a work that addresses extensive content (e.g. description and discussion on multiple outcomes in numerous groups – ethnic groups, clusters and ≥ 200 long-term conditions). However, we understand that paragraph two in the Methods section is indispensable because it describes the criteria used to select the study population. On the other hand, as suggested, we moved the details about the process used to define multimorbidity to the supporting information file.

The following paragraph was moved from Methods section to the S1 Supporting information. Selection of the Long-Term Conditions: *“Following a two-round consensus exercise composed of ten clinicians from diverse specialities, 204 LTCs were included in our operational definition of multimorbidity from a base list of 311 LTCs, derived from existing high quality studies and codelist resources studies [16,17,23–25]. The codelist revision, development and curation consisted of the following approaches – (a). Comparison between existing codelists followed by clinical curation, (b). Development of new codelists through relevant medical terms and, (c). Cross-mapping of different code systems using revised codelists as a baseline. We revised 263 codelists built on Read v2, 214 codelists built on ICD-10 (International Classification of Diseases, Tenth Revision), 13 codelists built on OPCS-4 (Classification of Interventions and Procedures version 4), and 2 codelists built on gemscript codes (prescribing codes).”*

Additionally, to be consistent with that, we also moved the following paragraph from the Results section to the S1 Supporting information. Selection of the Long-Term Conditions: *“Using the consensus exercise outlined, we extensively revised and selected codelists for 204 LTCs defined by 11,053 Read v2 codes, 2,594 ICD-10 codes, 747 OPCS-4 codes, and 3,829 gemscript codes. Detailed information on the codelist curation and development and all codelists used in our study can be found in the MULTIPLY-Initiative online repository [27].”*

**Comment 3:**

**Which ethnic groups were excluded from the study and how many people did this involve - this is in supplementary but might be useful to document in methods (with %).**

Thank you for your suggestion. We have specified the ethnic groups excluded in the flow chart for the section of our study population (Fig 1) to make it more detailed. This flowchart was moved to the main body of the paper following a request from the editors. The reason for not describing the excluded ethnic groups in the methods section is that we rather focused on the detailed description of the included ethnic groups as per the inclusion criteria. However, we did mention the exclusion as one of our study limitations. Please, see edits below:

Abstract section (page 3, lines 66-69): *“Study limitations include the exclusion of individuals with missing ethnicity information, the age of diagnosis not reflecting the actual age of onset and the exclusion of people from Mixed, Chinese and other ethnic groups due to insufficient power to investigate associations between multimorbidity and health-related outcomes in these groups.”*

Strengths and limitations (page 24, lines 478-481): *“Besides, other ethnic groups were not included in the analysis, for instance, Mixed and Chinese or other group. The reason was the small population size within those ethnic group that fulfilled our study eligibility criteria, resulting in inadequate power to derive robust clusters of multimorbidity and examine associations with health outcomes and mortality.”*

**Comment 4:**

**Could any of the conditions be double counted, could this be a limitation when looking at multimorbidity but could be a strength when looking at condition accrual. Access to the code lists publicly would be very helpful for other researchers.**

Thank you for your question. We considered prevalent cases and only the first occurrence of a long-term condition ever recorded in the patient’s electronic health record. All possible duplicates and double-counted conditions were removed in the data-cleaning process before the start of the statistical analysis. Please, see below:

Methods section (page 7, lines 183-185): *“Using all data available from the individual’s primary care and secondary care health records we identified each LTC using the first relevant code ever recorded and calculated the age at diagnosis by subtracting the year of birth from the year of diagnosis. If a condition was never recorded it was considered absent.*

The codelists are publicly available in an online repository. Please, see mention below:

Methods section (page 7, lines 181-182): “). Detailed information on the methods used to curate the codelists, and the codelists themselves, are available in the MULTIPLY-Initiative online repository.”

**Comment 5:**

**Results. Some of the results section text could be in methods/supplementary, for example paragraph 4 "After evaluating first statistics for the latent class models.." where methods are described.**

Thank you very much for your comment. Following your suggestion, the detailed information on the evaluation of the fit statistics was moved from the Methods section to the S2 Supporting information (Fit statistics for the model selection).

In the paragraph cited above, in the Results section, we only paraphrased a step of the methods to justify the number of clusters described throughout the results. However, the full description of the methods applied to evaluate the fit statistics and obtain the number of clusters is now only described in the Supporting information file.

The following paragraph was moved from the Methods section to the S2 Supporting information (Fit statistics for the model selection): *“We obtained and compared the fit statistics for each k-class model, which along with clinical judgement – on the clusters that besides being clinically meaningful also captured heterogeneous LTCs – supported the selection of the optimal number of latent classes. We obtained the bootstrapped likelihood ratio test (BLRT) which indicates if a model with k classes is statistically better than a model with k–1 class upon a provided p-value [35]. We evaluated the Bayesian Information Criterion (BIC), the Sample-size Adjusted Bayesian Information Criterion (SABIC), and the likelihood ratio test (LRT) to compare competing models. They inform how much a model is improved by an additional class. Lower BIC, SABIC and LRT values indicate a better-fit model. The entropy was assessed to verify the model quality since it indicates how accurately the model defines classes. In general, a value closer to 1 is desirable, although there is no agreed-upon cutoff criterion for entropy [36].”*

**Disclaimer**

This study is based in part on data from the Clinical Practice Research Datalink obtained under licence from the UK Medicines and Healthcare products Regulatory Agency. The data is provided by patients and collected by the NHS as part of their care and support. The interpretation and conclusions contained in this study are those of the authors alone. The ONS is the provider of the ONS Data contained within the Dataset. HES and ONS data were re-used with the permission of The Health & Social Care Information Centre. All rights reserved. The OPCS Classification of Interventions and Procedures, codes, terms and text is Crown copyright (2016) published by Health and Social Care Information Centre, also known as NHS Digital and licensed under the Open Government Licence available at www.nationalarchives.gov.uk/doc/open-government-licence/open-governmentlicence.

Htm.

**Data sharing**

The Clinical Practice Research Datalink (CPRD) does not allow the sharing of patient-level data. The structure and format of the CPRD data set is available at: https://cprd. com/sites/default/files/CPRD%20GOLD%20Full %20Data%20Specification%20v2.0_0.pdf. The list of long-term conditions and respective code lists used in this study are available at: <https://github.com/Fabiola-Eto/MULTIPLY-Initiative>.

The data that support the findings of this study are available from CPRD and access is subject to approval from an Independent Scientific Advisory Committee (ISAC). The data were used under license for the current study, and so are not publicly available.

**Acknowledgements**

The authors would like to thank Sherman Lo, research software engineer in the ITS Research, Queen Mary University of London, for assistance in expanding and improving the poLCA R library by creating the poLCAParallel R package and enabling a much faster run of our codes. The package is available at: <https://github.com/QMUL/poLCAParallel/releases/tag/v1.1.0> . This research utilised Queen Mary's Apocrita HPC facility, supported by QMUL Research-IT. <http://doi.org/10.5281/zenodo.438045>.
