# Supplementary material for: Ethnic differences in early onset multimorbidity and associations with health service use, long-term prescribing, years of life lost, and mortality: A cross-sectional study using clustering in the UK Clinical Practice Research Datalink
Source: PLoS Med. 2023 Oct 27;20(10):e1004300. doi: 10.1371/journal.pmed.1004300 (PMC10610074; doi:10.1371/journal.pmed.1004300)
Supplement: Attachment — Submitted filename: Response to editors.docx [file pmed.1004300.s011.docx]

Dear PLoS Medicine editors,

We are delighted to learn about your decision to accept our paper for publication in PLoS Medicine upon this last review.

We have addressed the specific points made by the editors and indicated the changes made in the manuscript.

Thank you very much again for the opportunity to have our work published in the journal. We look forward to reading our paper in your journal.

Yours sincerely,

Dr Fabiola Eto

**Requests from the editors**

**Request 1:**

**At line 43, for example, please use square brackets ("... conditions [LTCs] ...") within brackets.**

Thank you for your comment. We have added the square brackets as below:

Abstract section (page 2, line 43): *("... conditions [LTCs] ...")*

**Request 2:**

**At line 53, please** **substitute "identified", or similar, for "unveiled".**

Thank you. We have substituted "identified" for "unveiled" as suggested:

Abstract section (page 2, line 53*)*: *“Latent class analysis stratified by ethnicity identified 4 clusters…”*

**Request 3:**

**At line 59, please revisit "the White population was predominantly male ...". Perhaps some information about the group referred to is needed (otherwise this statement would seem inconsistent with information in table 1).**

Thank you for your suggestion. We have made some edits to make sure that the statement reads more clearly. Please, see below:

Abstract section (page 3, lines 58-62*)*: *“However, each ethnic group also presented exclusive LTCs and different sociodemographic profiles: in White groups, the cluster with the highest rates/odds of the outcomes was predominantly male (54%, 44,150) and more socioeconomically deprived than the cluster with the lowest rates/odds of the outcomes. On the other hand, South Asian and Black groups were more socioeconomically deprived than White groups, with a consistent deprivation gradient across all multimorbidity clusters.”*

**Request 4:**

**At line 64, please adapt the punctuation to****: "... respectively); however, the ...".**

Thank you for your suggestion. We have corrected the punctuation:

Abstract section (page 3, line 64): *"... respectively); however, the ...".*

**Request 5:**

**At line 123, please make that "the White group".**

We have made the edits suggested:

Introduction section (page 5, line 123): “… *compared to the White group.”*

**Request 6:**

**At line 140, please substitute a colon for the semicolon.**

Thank you. We have substituted a colon for the semicolon:

Introduction section (page 6, line 139-140): *“… the aims of this study were: firstly, to develop …”*

**Request 7:**

**At line 162 and any other instances, please make that "... 39 years".**

Thank you for suggesting that. We have added “years” where necessary:

Methods section (page 7, line 162): *“… ages of 16 and 39 years.”*

Results section (page 10, line 246): *“… people in the CPRD GOLD aged 16 years and over …”*

Results section (page 11, line 265): *“(median age = 52, 48 and 61 years, respectively)”*

**Request 8:**

**At line 211, "age in 2010"?**

We have substituted “age in 2010” for “age at 2010” at line 211 and where else to observe consistency:

Methods section, page 9, line 211

Methods section, page 10, line 235

Table 1, first column, row 7

Table 2, first column, row 6

Table 3, title and first column, row 9

Supporting information, page 29, line 668

S1 Figure, second boxplot, x-axis label

**Request 9:**

**At line 255, we suggest quoting the number for the White population with early-onset multimorbidity alongside the percentage.**

Thank you for this suggestion. We have quoted the number for the White population alongside the percentage:

Results section (page 10, line 255): *“… White population (42%, n = 777,906).”*

**Request 10:**

**At line 310, please substitute a colon for the semicolon.**

We have substituted a colon for the semicolon as below:

Results section (page 13, line 310): *“…. according to ethnicity: COPD, CHD, …”*

**Request 11:**

**At line 354, please make that "people in cluster 1".**

We have done as suggested:

Results section (page 20, line 354): *“Compared to people in cluster 1 …”*

**Request 12:**

**At line 359, "lost an average"?**

That is correct, thanks. We have corrected it:

Results section (page 20, line 359): *“Individuals in cluster 3 lost an average …”*

**Request 13:**

**At line 376, we suggest substituting "... as compared to Whites".**

Thanks for your suggestion. We have made the substitution:

Discussion section (page 20, line 376): *“… as compared to Whites.”*

**Request 14:**

**At line 415, we suggest making that "year 5" and "year 10".**

We have done as suggested, please see below:

Discussion section (page 22, line 415): *“… by the end of year 5 than by the end of year 10.”*

**Request 15:**

**At line 443, there may be some repetition in "older ages and older": please check.**

Thank you for noticing that repetition. We have removed it:

Discussion section (page 23, line 443): *“… as individuals with longer follow-up time and older ages ...”*

**Request 16:**

**At line 465, "reverse causality"?**

Thank you. We have corrected the mistyping:

Strengths and limitations section (page 24, line 465): *“… susceptible to reverse causality ...”*

**Request 17:**

**At line 489, please make that "Finally ...".**

Indeed, “finally” reads much better, thank you.

Strengths and limitations section (page 24, line 489): “*Finally, there may be ...”*

**Request 18:**

**At line 493, "minority ethnic populations"?**

Thank you. We have added “population” to the statement:

Conclusion section (page 25, line 493): “… *among minority ethnic populations ...”*

**Request 19:**

**In table 1, second column, row 7, adapt to "165,180"?**

Thank you. We have corrected it as suggested.

Table 1 (second column, row 7): *“165,180”*

**Request 20:**

**Please remove the information on data sharing from the end of the main text. In the event of publication, this will appear in the article metadata, via entries in the submission form.**

We have removed the information on data sharing from the end of the main text, thank you for suggesting that.

**Request 21:**

**Noting reference 2, please remove all iterations of "[Internet]" from the reference list.**
Thank you for suggesting that. We have removed all iterations of [Internet] from the following references: 2, 3, 31 and 37.

**Reviewer #1**

**Comment:**

**Thanks for the revised manuscript and responses to my queries. The updates to the manuscript have resolved my original questions - this is an interesting study I enjoyed reviewing. I appreciate the information on the parallel version of poLCA, I have been through the pain of LCA with a large dataset before.**

Thank you so much for your comments, questions, and relevant suggestions. We are happy that the information on the parallel version of poLCA is helpful for you, and we hope that that will be relevant for many other researchers and users facing the challenge of large datasets and computational costs as well.

**Reviewer #2**

**Comment:**

**I thank the authors for their thoughtful replies to my comments. I have nothing further.**

Thank you so much for your contribution to improving our manuscript through your comments, questions and suggestions.
